# Zinc-Catalyzed Enantioselective [3 + 3] Annulation for Synthesis of Chiral Spiro[indoline-3,4′-thiopyrano[2,3-*b*]indole] Derivatives

**DOI:** 10.3390/molecules28031056

**Published:** 2023-01-20

**Authors:** Tian-Tian Liu, Yu Chen, Guang-Jian Mei, Yuan-Zhao Hua, Shi-Kun Jia, Min-Can Wang

**Affiliations:** Green Catalysis Center, College of Chemistry, Zhengzhou University, Zhengzhou 450001, China

**Keywords:** dinuclear zinc catalyst, asymmetric catalytic reaction, [3 + 3] annulation, indoline-2-thione, isatylidene malononitrile

## Abstract

With a dinuclear zinc-ProPhenol complex as a catalyst, an efficient and novel [3 + 3] annulation of indoline-2-thiones and isatylidene malononitriles has been successfully developed via the Brønsted base and Lewis acid cooperative activation model. This practical methodology gives access to a broad range of chiral spiro[indoline-3,4′-thiopyrano[2,3-*b*]indole] derivatives in good yields with excellent levels of enantioselectivities (up to 88% yield and 99% ee).

## 1. Introduction

Sulfur-containing heterocyclic compounds are prevalent in various numerous pharmaceutically useful molecules and natural products [1,2,3]. Among these, the thiopyran fused indole skeleton is an attractive structural moiety because of its remarkable biological activities [4,5,6]. For instance, the tetrahydrothiopyrano [2,3-*b*]indole (THTPI) derivatives exhibit analgesic activities. On the other hand, the oxindole frameworks bearing a spirocyclic quaternary stereocenter at the C3 position [7,8,9,10,11] are very privileged heterocyclic motifs owing to their wide ranging biological significance and high versatility as important building blocks. They have attracted widespread attention from chemists for their intrinsic complexity as well as rigidity. As is expected, integrating these two bioactive moieties to generate a fascinating spirocyclic skeleton, spiro[indoline-3,4′-thiopyrano[2,3-*b*]indole], would be synthetically valuable and pharmaceutically desirable, and might offer a structurally diversified molecules library for drug discovery.

**Scheme 1:**
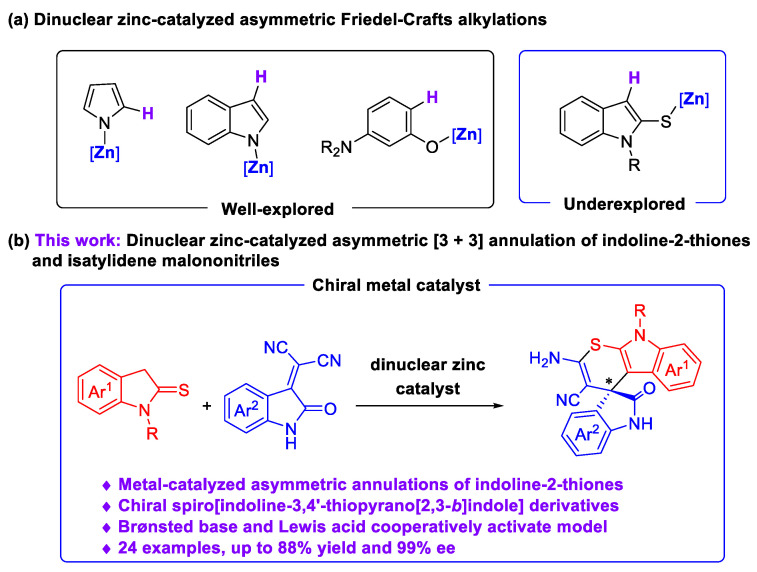
Previous reports and this work. ((**a**) Dinuclear zinc-catalyzed asymmetric Freidel-Crafts alkylation’s (**b**) This work: Dinuclear zinc-catalyzed asymmetric [3 + 3] annulation of indoline-2-thiones and isatylidene malononitriles.).

The Friedel–Crafts reaction [12,13] has emerged as a powerful chemical tool for the construction of new C–C bonds, and it has been widely used to realize asymmetric transformations of aromatics and heteroaromatics [14,15]. In the past few years, dinuclear metal-ProPhenol catalysts, which feature both Lewis acidic and Brønsted basic sites, have emerged as a powerful chemical tool for asymmetric transformations and attracted much attention in this field [16,17,18]. For instance, the Trost group [19] first reported the use of a dinuclear zinc complex in Friedel–Crafts alkylations of unprotected pyrroles with nitroalkenes via deprotonation of the amino group in 2008. Subsequently, the same strategy was successfully applied to the catalytic asymmetric Friedel–Crafts alkylations of unprotected pyrroles [20] and indoles [21,22,23] with various electrophiles. Recently, our group [24] uncovered an enantioselective Friedel–Crafts alkylation/cyclization with a dinuclear zinc complex through deprotonation of the phenolic hydroxyl group of 3-aminophenols. However, the catalytic generation of active carbon nucleophiles from thioamides via deprotonation of the sulfydryl group with the dinuclear zinc catalysts (Scheme 1a) is underexplored.

Thioamides are widely utilized as useful precursors for the synthesis of a broad range of heterocyclic compounds by exploiting their multiple nucleophilic characters [25]. The employment of thioamides as carbon pronucleophiles in enantioselective C-C bond-forming reactions is challenging in view of the competitions of the S–H functionalization or the N–H functionalization. To solve this problem, the Shibasaki group [26,27,28,29,30,31,32,33] strategically developed a soft Lewis acid/Brønsted base cooperative catalyst to generate the active carbon nucleophile from thioamides by exploiting the soft Lewis basic nature of the sulfur atom. As a kind of thioamides, indoline-2-thiones usually serve as 1,3-binucleophilic synthons for the construction of various indole-fused sulfur-containing ring structures [34,35,36]. In recent years, many asymmetric organocatalytic strategies [37,38,39,40,41,42,43] successfully applied to the indoline-2-thiones involved cascade annulations. For example, in 2014, Wang group [37] developed a highly efficient asymmetric cascade thio-Michael cyclization reaction by a DPEN-derived chiral thiourea. However, there are no reports on metal-catalyzed asymmetric cascade annulations of the indoline-2-thiones. Herein, we describe the application of Bronsted base and Lewis acid cooperative activation [44,45,46,47] to the synthesis of spiro[indoline-3,4′-thiopyrano[2,3-*b*]indole] derivatives via the Friedel–Crafts alkylation/cyclization tandem reactions of indoline-2-thiones and isatylidene malononitriles [48,49,50,51] (Scheme 1b).

## 2. Results

### 2.1. Optimization of Reaction Conditions

Initially, we investigated the reaction of 1-methylindoline-2-thione **1a** and 2-(2-oxoindolin-3-ylidene)malononitrile **2a** in the presence of 10 mol % of dinuclear zinc catalyst in situ generated from 10 mol % of ligand **L1** and 20 mol % of ZnEt_2_ in tetrahydrofuran (THF) at rt. Delightfully, the cascade [3 + 3] annulation process proceeded smoothly to furnish the desired spirocyclic product **3a** in 57% yield and 80% enantioselectivity (Table 1, entry 1). Encouraged by those promising results, we next examined a series of chiral ligands, including ProPhenol ligands (**L2**–**L5**) and AzePhenol ligands (**L7**–**L9**). The screening results indicated that CF_3_ substituted ProPhenol ligands were better promotors compared with other substitutional groups, and **L4** bearing 4-CF_3_C_6_H_4_ group was proven to be the best choice for this cascade transformation, which provided the cyclization product **3a** in 85% yield and 99% ee (Table 1, entry 4).

### 2.2. Substrate Scope

**Scheme 2:**
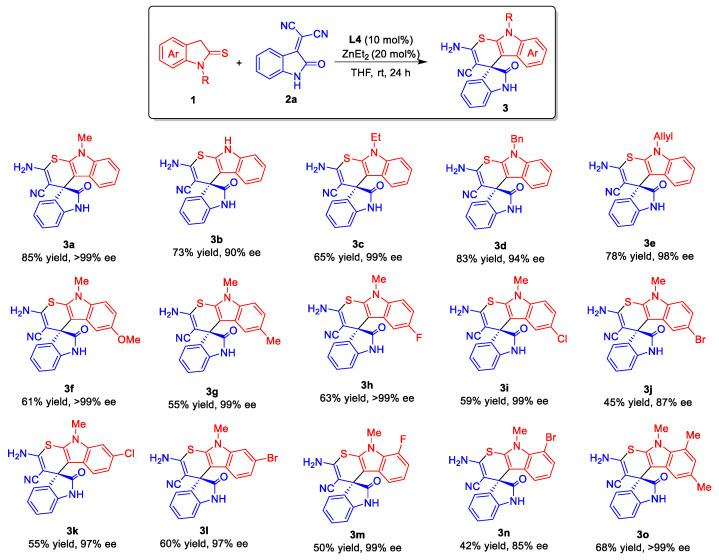
Substrate scope of indoline-2-thiones. Reaction conditions: 10 mol % of **L4**, 20 mol % of ZnEt_2_, 0.20 mmol **1** and 0.20 mmol **2a** in 2 mL THF.

With the optimized reaction conditions in hand, we first examined the substrate scope of indoline-2-thiones **1** by reacting with 2-(2-oxoindolin-3-ylidene)malononitrile **2a**, and the results are summarized in Scheme 2. Firstly, the influence of the *N*-protecting groups of indoline-2-thiones was investigated. Interestingly, when the *N*-H indoline-2-thiones was tried, the corresponding product **3b** was produced in 73% yield and 90% ee. As for the substrates of *N*-ethyl, *N*-benzyl, *N*-allyl substituted indoline-2-thiones, all the [3 + 3] annulations gave the expected products **3c**–**3e** in 65–83% yields and 94–99% ee values, respectively. Subsequently, indoline-2-thiones **1** bearing substituents (from electron donating to electron with-drawing) at the C-5 position were well tolerated for this cyclization, which furnished the corresponding products **3f**–**3j** in 45–63% yields with 87–99% ee values. Furthermore, substitutions at the 6-position (6-Cl and 6-Br) and 7-position (7-F and 7-Br) were also tolerated, giving the corresponding products **3k**–**3n** in good yields with 85–99% ee values. It was worth noting that the Br substituent at the 5 or 7-position of the indoline-2-thione had a negative effect on both the yield and enantioselectivity. Gratifyingly, 5,7-diMe substituted indoline-2-thione could also be subject to this transformation, delivering the annulation product **3o** in 68% yield and 99% ee.

**Scheme 3:**
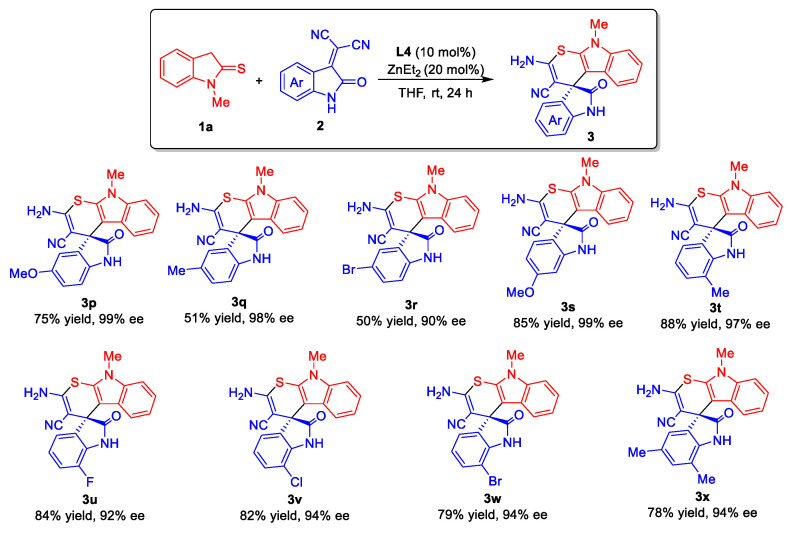
Substrate scope of isatylidene malononitriles. Reaction conditions: 10 mol % of **L4**, 20 mol % of ZnEt_2_, 0.20 mmol **1a** and 0.20 mmol **2** in 2 mL THF.

Next, we explored the substrate generality of isatylidene malononitriles in this [3 + 3] annulation by focusing our attention on the reaction of the 1-methylindoline-2-thione **1a** with various isatylidene malononitriles **2**, and the results are summarized in Scheme 3. In general, all the reactions proceeded favourably to afford the desired products in good yields. It was proven that the isatylidene malononitriles bearing electron-withdrawing or -donating groups at the 5-, 6-, and 7-positions were well tolerated for this transformation, which furnished the corresponding products **3p**–**3x** in 50–88% yields with 90–99% ee values.

### 2.3. X-ray Diffraction Analysis

**Figure 1:**
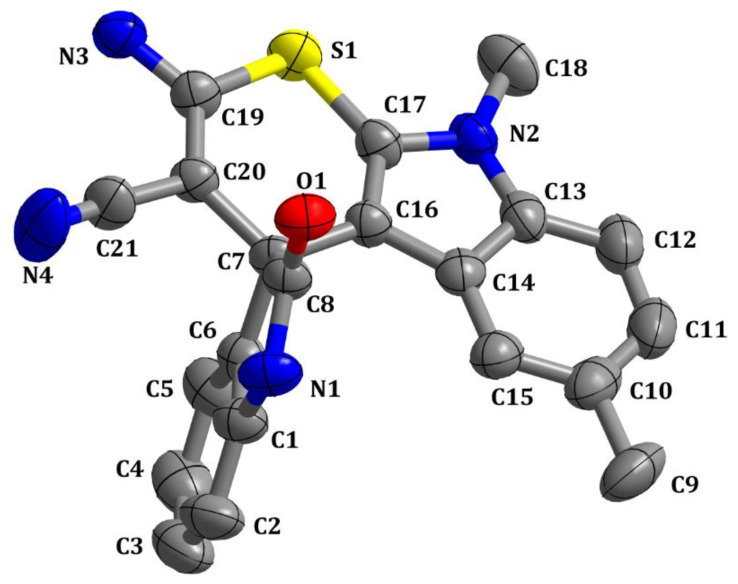
Crystal structures of **3g** (CCDC 2177975) detected by Bruker APEX-II CCD (the thermal ellipsoids are drawn at a 30% probability level).

The relative and absolute configurations of the product **3g** were unequivocally determined as *R* by single crystal X-ray diffraction analysis [52] and applied to all other products **3** (Figure 1). Single crystal X-ray diffraction is shown in Supplementary Materials Figure S54.

### 2.4. Gram-Scale Reaction and Derivation

**Scheme 4:**
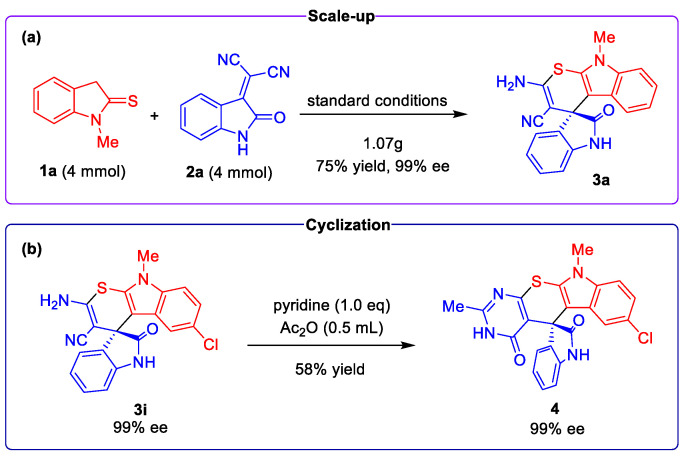
Gram-scale reaction (**a**) and derivation (**b**).

To demonstrate the potential utility of this methodology, a gram-scale synthesis of **1a** (4 mmol) and **2a** (4 mmol) was performed, and the [3 + 3] annulation reactions occurred smoothly under standard conditions, giving the spiro[indoline-3,4′-thiopyrano[2,3-*b*]indole] **3a** in 75% yield (1.07 g) and 99% ee (Scheme 4a). Further transformation of **3i** was preformed, the cyano and amino groups of **3i** underwent annulations with acetic anhydride in pyridine, affording cyclization product **4** in 58% yield and 99% ee (Scheme 4b).

### 2.5. Plausible Mechanism

**Scheme 5:**
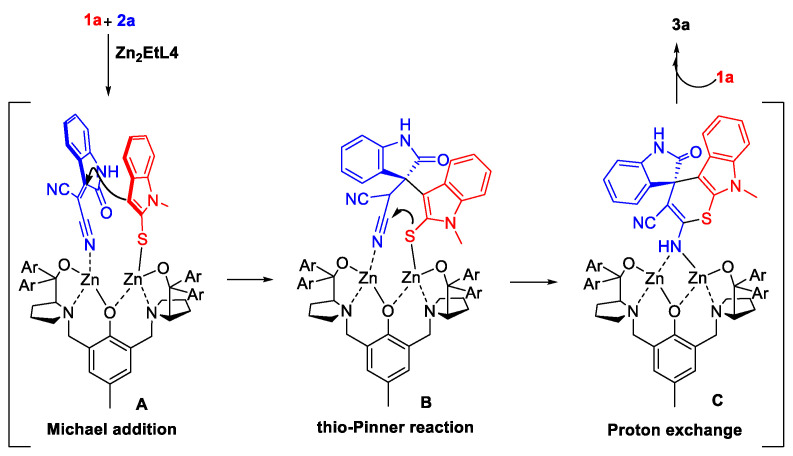
Plausible mechanism.

Based on the experimental results and previous literature reports [24,53], a plausible reaction mechanism for this Friedel–Crafts alkylation/cyclization is illustrated in Scheme 5. First, the dinuclear zinc complex Zn_2_EtL4 was prepared in situ when ligand **L4** is treated with 2 equiv of Et_2_Zn. Then, indoline-2-thione **1a** was converted into its thioenolate form via the deprotonation process by the dinuclear zinc complex, along with the release of 1 equiv of ethane. Subsequently, the coordination of alkylidene azlactone **2a** from the less hindered face to both zinc atoms led to the formation of intermediate **A**, which underwent the Michael addition reaction and proton shift to afford the complex **B**. Next, an intramolecular thio-Pinner reaction proceeded to furnish the annulation. Finally, the catalytic cycle was restarted after a proton exchange of intermediate **C** with another indoline-2-thione **1a**, followed by the release of the activated species **A** and spiro[indoline-3,4′-thiopyrano[2,3-*b*]indole] **3a**.

## 3. Conclusions

In conclusion, we disclose a novel and efficient asymmetric tandem [3 + 3] annulation of indoline-2-thiones and isatylidene malononitriles. Using the chiral metal catalyst, a series of optically pure spiro[indoline-3,4′-thiopyrano[2,3-*b*]indole] derivatives were obtained with good to excellent yields and enantioselectivities under mild conditions. To demonstrate the promising applicability of the methodology, a gram-scale experiment, and the derivatization of the product were also successfully performed. A Brønsted base and Lewis acid cooperatively activate activation mode was proposed and further investigations in the polyfunctional heterocycles are ongoing in our laboratory.

## 4. Materials and Methods

### 4.1. General Information

All reactions were carried out under an atmosphere of argon using oven-dried glassware. Super dry solvents and metal catalysts were purchased from chemical companies and used without further treatment. Flash column chromatography was performed using silica gel (300–400 mesh). ^1^H NMR,^13^C NMR, ^19^F NMR spectra were recorded in DMSO-d_6_ on a 400 MHz spectrometer; chemical shifts are reported in ppm with the solvent signals as reference, and coupling constants (*J*) are given in Hertz. NMR spectra are given in the Supplementary Materials Figures S1–S28. The peak information is described as: s = singlet, d = doublet, t = triplet, q = quartet, m= multiplet. High-resolution mass spectra (HRMS) were obtained using an Agilent LC-MSAD-Trap-XCT instrument (Shanghai, China) using electrospray ionization time-of-flight (ESI-TOF). The high performance liquid chromatography (HPLC) performed on instrument consisted of the JASCO model PU-1580 intelligent HPLC pump (Zhengzhou, China) and the JASCO model UV-1575 intelligent UV-vis detector (254 nm) (Zhengzhou, China) using Daicel Chiralpak IA (4.6 mm × 250 mm) columns (Shanghai, China).HPLC are shown in Supplementary Materials Figures S29–S53. Melting points were determined using YRT-3 melting point apparatus. Optical rotations were measured with Perkin Elmer, model 341 Polarimeter (Zhengzhou, China). The instrumentation used for the crystal measurement is Bruker APEX-II CCD (Shanghai, China).

### 4.2. Materials

Indoline-2-thiones [54] and isatylidene malononitriles [55] were synthesized according to the literature. Other reagents were obtained from commercial sources and used without further purification.

### 4.3. Procedure for the Asymmetric Synthesis of Compound ***3***

Under a nitrogen atmosphere, a solution of diethylzinc (40 μL, 1.0 M in hexane, 0.04 mmol) was added dropwise to a solution of **L4** (0.02 mmol, 19.0 mg) in THF (2 mL). After the mixture was stirred for 30 min at room temperature, 1-methylindoline-2-thione **1a** (0.2 mmol, 32.6 mg) and 2-(2-oxoindolin-3-ylidene) malononitrile **2a** (0.2 mmol, 39.1 mg) were added. The reaction mixture was stirred for 24 h at the same temperature. The reaction was quenched with a HCl solution (1 M, 2 mL), and the organic layer was extracted with CH_2_Cl_2_ (3 × 5 mL). The combined organic layer was washed with brine and dried over Na_2_SO_4_. The solvent was removed under reduced pressure by using a rotary evaporator. The residue was purified by flash chromatography with petroleum ether/ethyl acetate (2:1) to afford the desired product **3**.

(*R*)-2′-amino-9′-methyl-2-oxo-9′*H*-spiro[indoline-3,4′-thiopyrano[2,3-*b*]indole]-3′-carbonitrile (**3a**). We followed the general procedure, using **1a** (0.2 mmol, 32.6 mg), **2a** (0.2 mmol, 39.1 mg), and **L4** (0.02 mmol, 19.0 mg). Purified by flash column chromatography (petroleum ether/ethyl acetate 2:1) to afford **3a** as a yellow solid (60.9 mg, 85% yield); [α]_D_^20^ = −3.21 (c = 1.0, EA, 99% ee); m.p. = 267.0–267.9 °C; ^1^H NMR (400 MHz, DMSO-d_6_) δ 10.72 (s, 1H), 7.43 (d, *J* = 8.2 Hz, 1H), 7.32 − 7.24 (m, 1H), 7.17 (s, 2H), 7.11 − 6.98 (m, 3H), 6.97 − 6.91 (m, 1H), 6.79 (t, *J* = 7.6 Hz, 1H), 6.36 (d, *J* = 8.1 Hz, 1H), 3.70 (s, 3H); ^13^C NMR (101 MHz, DMSO-d_6_) δ 178.3, 152.8, 142.0, 137.9, 134.1, 129.5, 125.5, 125.3, 124.4, 123.0, 121.8, 120.3, 118.0, 117.2, 110.3, 110.0, 103.3, 72.4, 52.3, 30.7; HRMS (ESI): *m*/*z* [M + H]^+^ calcd for [C_20_H_15_N_4_OS]^+^: 359.0961, found: 359.0959; HPLC: Daicel Chiralpak IA, n-hexane/i-PrOH = 70/30, flow rate = 1 mL/min, λ = 254 nm, t_major_ = 12.28 min and t_minor_ = 29.95 min.

(*R*)-2′-amino-2-oxo-9′*H*-spiro[indoline-3,4′-thiopyrano[2,3-*b*]indole]-3′-carbonitrile (**3b**). We followed the general procedure, using **1b** (0.2 mmol, 30.1 mg), **2a** (0.2 mmol, 39.1 mg), and **L4** (0.02 mmol, 19.0 mg). Purified by flash column chromatography (petroleum ether/ethyl acetate 2:1) to afford **3b** as a yellow solid (50.2 mg, 73% yield); [α]_D_^20^ =−10.69 (c = 1.0, EA, 90% ee); m.p. = 271.0–271.9 °C; ^1^H NMR (400 MHz, DMSO-d_6_) δ 11.64 (s, 1H), 10.72 (s, 1H), 7.29 (m, 2H), 7.06 (s, 2H), 7.02 − 6.92 (m, 4H), 6.75 (t, *J* = 7.5 Hz, 1H), 6.37 (d, *J* = 8.0 Hz, 1H). ^13^C NMR (101 MHz, DMSO-d_6_) δ 178.4, 153.6, 142.0, 136.9, 134.2, 129.4, 125.4, 124.7, 123.02, 122.95, 121.8, 120.0, 118.1, 117.0, 111.5, 110.2, 103.7, 72.1, 51.9; HRMS (ESI): *m*/*z* [M + H]^+^ calcd for [C_19_H_13_N_4_OS]^+^: 345.0805, found: 345.0805; HPLC: Daicel Chiralpak IA, n-hexane/i-PrOH = 70/30, flow rate = 1 mL/min, λ = 254 nm, t_minor_ = 9.50 min, and t_major_ = 23.29 min.

(*R*)-2′-amino-9′-ethyl-2-oxo-9′*H*-spiro[indoline-3,4′-thiopyrano[2,3-*b*]indole]-3′-carbonitrile (**3c**). We followed the general procedure, using **1c** (0.2 mmol, 35.2 mg), **2a** (0.2 mmol, 39.1 mg), and **L4** (0.02 mmol, 19.0 mg). Purified by flash column chromatography (petroleum ether/ethyl acetate 2:1) to afford **3c** as a yellow solid (46.9 mg, 65% yield); [α]_D_^20^ = −25.56 (c = 1.0, EA, 99% ee); m.p. = 265.5–266.4 °C; ^1^H NMR (400 MHz, DMSO-d_6_) δ 10.70 (s, 1H), 7.46 (d, *J* = 8.2 Hz, 1H), 7.29 (t, *J* = 7.6 Hz, 1H), 7.16 (s, 2H), 7.08 − 6.99 (m, 3H), 6.96 (m, 1H), 6.80 (t, *J* = 7.6 Hz, 1H), 6.39 (d, *J* = 8.0 Hz, 1H), 4.17 (m, 2H), 1.30 (t, *J* = 7.1 Hz, 3H); ^13^C NMR (101 MHz, DMSO-d_6_) δ 178.2, 152.7, 142.0, 136.9, 134.1, 129.5, 125.5, 124.6, 124.2, 123.0, 121.9, 120.3, 118.0, 117.3, 110.3, 109.9, 103.5, 72.5, 52.2, 39.2, 15.4; HRMS (ESI): *m*/*z* [M + H]^+^ calcd for [C_21_H_17_N_4_OS]^+^: 373.1118, found: 373.1120; HPLC: Daicel Chiralpak IA, n-hexane/i-PrOH = 70/30, flow rate = 1 mL/min, λ = 254 nm, t_major_ = 10.44 min, and t_minor_ = 31.64 min.

(*R*)-2′-amino-9′-benzyl-2-oxo-9′*H*-spiro[indoline-3,4′-thiopyrano[2,3-*b*]indole]-3′-carbonitrile (**3d**). We followed the general procedure, using **1d** (0.2 mmol, 48.2 mg), **2a** (0.2 mmol, 39.1 mg), and **L4** (0.02 mmol, 19.0 mg). Purified by flash column chromatography (petroleum ether/ethyl acetate 2:1) to afford **3d** as a yellow solid (72.1 mg, 83% yield); [α]_D_^20^ = −4.78 (c = 1.0, EA, 94% ee); m.p. = 247.9–248.6 °C; ^1^H NMR (400 MHz, DMSO-d_6_) δ 10.77 (s, 1H), 7.47 (d, *J* = 8.3 Hz, 1H), 7.38 − 7.28 (m, 4H), 7.13 (m, 4H), 7.07 − 7.01 (m, 3H), 6.99 − 6.94 (m, 1H), 6.81 (t, *J* = 7.8 Hz, 1H), 6.42 (d, *J* = 8.0 Hz, 1H), 5.43 (s, 2H); ^13^C NMR (101 MHz, DMSO-d_6_) δ 178.2, 152.7, 142.0, 137.7, 137.4, 134.1, 129.6, 129.3, 128.1, 127.0, 125.5, 125.1, 124.7, 123.0, 122.1, 120.6, 117.9, 117.4, 110.34, 110.31, 104.1, 72.4, 52.3, 47.4; HRMS (ESI): *m*/*z* [M + H]^+^ calcd for [C_26_H_19_N_4_OS]^+^: 435.1274, found: 435.1275; HPLC: Daicel Chiralpak IA, n-hexane/i-PrOH = 70/30, flow rate = 1 mL/min, λ = 254 nm, t_major_ = 11.92 min, and t_minor_ = 39.08 min.

(*R*)-9′-allyl-2′-amino-2-oxo-9′*H*-spiro[indoline-3,4′-thiopyrano[2,3-*b*]indole]-3′-carbonitrile (**3e**). We followed the general procedure, using **1e** (0.2 mmol, 35.5 mg), **2a** (0.2 mmol, 39.1 mg), and **L4** (0.02 mmol, 19.0 mg). Purified by flash column chromatography (petroleum ether/ethyl acetate 2:1) to afford **3e** as a yellow solid (59.9 mg, 78% yield); [α]_D_^20^ = −47.31 (c = 1.0, EA, 98% ee); m.p. = 292.1–292.9 °C; ^1^H NMR (400 MHz, DMSO-d_6_) δ 10.74 (s, 1H), 7.42 (d, *J* = 8.3 Hz, 1H), 7.28 (m, 1H), 7.13 (s, 2H), 7.10 − 6.98 (m, 3H), 6.94 (t, *J* = 7.5 Hz, 1H), 6.80 (t, *J* = 7.6 Hz, 1H), 6.39 (d, *J* = 8.0 Hz, 1H), 6.26 − 5.46 (m, 1H), 5.33 − 4.99 (m, 1H), 5.00 − 4.84 (m, 1H), 4.79 (d, *J* = 4.4 Hz, 2H); ^13^C NMR (101 MHz, DMSO-d_6_) δ 176.3, 150.9, 140.1, 135.5, 132.2, 131.5, 127.6, 123.5, 123.0, 122.6, 121.1, 120.0, 118.5, 116.0, 115.6, 115.4, 108.4, 108.3, 102.0, 70.5, 50.3, 44.5; HRMS (ESI): *m*/*z* [M + H]^+^ calcd for [C_22_H_17_N_4_OS]^+^: 385.1118, found: 385.1117; HPLC: Daicel Chiralpak IA, n-hexane/i-PrOH = 70/30, flow rate = 1 mL/min, λ = 254 nm, t_major_ = 10.18 min, and t_minor_ = 31.62 min.

(*R*)-2′-amino-6′-methoxy-9′-methyl-2-oxo-9′*H*-spiro[indoline-3,4′-thiopyrano[2,3-*b*]indole]-3′-carbonitrile (**3f**). We followed the general procedure, using **1f** (0.2 mmol, 39.3 mg), **2a** (0.2 mmol, 39.1 mg), and **L4** (0.02 mmol, 19.1 mg). Purified by flash column chromatography (petroleum ether/ethyl acetate 2:1) to afford **3f** as a yellow solid (47.3 mg, 61% yield); [α]_D_^20^ = + 1.92 (c = 1.0, EA, 99% ee); m.p. = 278.1–278.9 °C; ^1^H NMR (400 MHz, DMSO-d_6_) δ 10.72 (s, 1H), 7.37 − 7.27 (m, 2H), 7.18 (s, 2H), 7.06 − 6.94 (m, 3H), 6.74 − 6.65 (m, 1H), 5.76 (d, *J* = 2.4 Hz, 1H), 3.66 (s, 3H), 3.43 (s, 3H); ^13^C NMR (101 MHz, DMSO-d_6_) δ 178.2, 154.0, 153.1, 142.2, 133.9, 133.2, 129.6, 125.63, 125.55, 124.8, 123.0, 118.1, 110.63, 110.58, 110.1, 102.8, 100.1, 72.1, 55.4, 52.2, 30.8; HRMS (ESI): *m*/*z* [M + H]^+^ calcd for [C_21_H_17_N_4_O_2_S]^+^: 389.1067, found: 389.1075; HPLC: Daicel Chiralpak IA, n-hexane/i-PrOH = 70/30, flow rate = 1 mL/min, λ = 254 nm, t_major_ = 14.13 min, and t_minor_ = 32.41 min.

(*R*)-2′-amino-6′,9′-dimethyl-2-oxo-9′*H*-spiro[indoline-3,4′-thiopyrano[2,3-*b*]indole]-3′-carbonitrile (**3g**). We followed the general procedure, using **1g** (0.2 mmol, 35.6 mg), **2a** (0.2 mmol, 39.1 mg), and **L4** (0.02 mmol, 19.0 mg). Purified by flash column chromatography (petroleum ether/ethyl acetate 2:1) to afford **3g** as a yellow solid (41 mg, 55% yield); [α]_D_^20^ = −9.26 (c = 1.0, EA, 99% ee); m.p. = 283.6–284.4 °C; ^1^H NMR (400 MHz, DMSO-d_6_) δ 10.69 (s, 1H), 7.39 − 7.24 (m, 2H), 7.15 (s, 2H), 7.09 − 6.98 (m, 2H), 6.95 (m, 1H), 6.88 (d, *J* = 10.1 Hz, 1H), 6.11 (s, 1H), 3.67 (s, 3H), 2.11 (s, 3H); ^13^C NMR (101 MHz, DMSO-d_6_) δ 178.3, 152.8, 142.0, 136.4, 134.2, 129.5, 128.7, 125.4, 125.0, 124.6, 123.2, 123.0, 118.0, 117.0, 110.2, 109.7, 102.8, 72.4, 52.3, 30.7, 21.7; HRMS (ESI): *m*/*z* [M + Na]^+^ calcd for [C_21_H_16_N_4_OSNa]^+^: 395.0937, found: 395.0944; HPLC: Daicel Chiralpak IA, n-hexane/i-PrOH = 70/30, flow rate = 1 mL/min, λ = 254 nm, t_major_ = 11.09 min, and t_minor_ = 23.70 min.

(*R*)-2′-amino-6′-fluoro-9′-methyl-2-oxo-9′*H*-spiro[indoline-3,4′-thiopyrano[2,3-*b*]indole]-3′-carbonitrile (**3h**). We followed the general procedure, using **1h** (0.2 mmol, 36.2 mg), **2a** (0.2 mmol, 39.1 mg), and **L4** (0.02 mmol, 19.0 mg). Purified by flash column chromatography (petroleum ether/ethyl acetate 2:1) to afford **3h** as a yellow solid (47.4 mg, 63% yield); [α]_D_^20^ = −2.59 (c = 1.0, EA, 99% ee); m.p. = 296.8–297.4 °C; ^1^H NMR (400 MHz, DMSO-d_6_) δ 10.76 (s, 1H), 7.47 (m, 1H), 7.32 (t, *J* = 8.3 Hz, 1H), 7.24 (s, 2H), 7.12 − 7.01 (m, 2H), 7.01 − 6.89 (m, 2H), 6.14 − 5.72 (m, 1H), 3.71 (s, 3H); ^13^C NMR (101 MHz, DMSO-d_6_) δ 178.0, 157.5 (d, *J* = 233.4 Hz), 152.8, 142.0, 134.6, 133.5, 129.8, 127.5, 125.6, 124.4 (d, *J* = 10.4 Hz), 123.1, 117.9, 111.4 (d, *J* = 10.1 Hz), 110.3, 109.7 (d, *J* = 26.0 Hz), 103.3 (d, *J* = 4.4 Hz), 102.0 (d, *J* = 24.6 Hz), 72.0, 52.1, 31.0; ^19^F NMR (376 MHz, DMSO-d_6_) δ -124.46; HRMS (ESI): *m*/*z* [M + H]^+^ calcd for [C_20_H_14_FN_4_OS]^+^: 377.0867, found: 377.0868; HPLC: Daicel Chiralpak IA, n-hexane/i-PrOH = 70/30, flow rate = 1 mL/min, λ = 254 nm, t_major_ = 11.12 min, and t_minor_ = 34.71 min.

(*R*)-2′-amino-6′-chloro-9′-methyl-2-oxo-9′*H*-spiro[indoline-3,4′-thiopyrano[2,3-*b*]indole]-3′-carbonitrile (**3i**). We followed the general procedure, using **1i** (0.2 mmol, 39.6 mg), **2a** (0.2 mmol, 39.1 mg), and **L4** (0.02 mmol, 19.0 mg). Purified by flash column chromatography (petroleum ether/ethyl acetate 2:1) to afford **3i** as a yellow solid (46.4 mg, 59% yield); [α]_D_^20^ = −6.15 (c = 1.0, EA, 99% ee); m.p. = 289.8–290.7 °C; ^1^H NMR (400 MHz, DMSO-d_6_) δ 10.80 (s, 1H), 7.49 (d, *J* = 8.8 Hz, 1H), 7.33 (t, *J* = 8.3 Hz, 1H), 7.25 (s, 2H), 7.20 − 7.01 (m, 3H), 6.98 (t, *J* = 7.7 Hz, 1H), 6.25 (d, *J* = 2.0 Hz, 1H), 3.71 (s, 3H): ^13^C NMR (101 MHz, DMSO-d_6_) δ 178.0, 152.7, 141.9, 136.4, 133.6, 129.8, 127.6, 125.5, 125.2, 124.9, 123.2, 121.6, 117.8, 116.2, 111.8, 110.3, 103.1, 72.1, 52.0, 31.0; HRMS (ESI): *m*/*z* [M + H]^+^ calcd for [C_20_H_14_ClN_4_OS]^+^: 393.0571, found: 393.0569; HPLC: Daicel Chiralpak IA, n-hexane/i-PrOH = 70/30, flow rate = 1 mL/min, λ = 254 nm, t_major_ = 11.54 min, and t_minor_ = 36.99 min.

(*R*)-2′-amino-6′-bromo-9′-methyl-2-oxo-9′*H*-spiro[indoline-3,4′-thiopyrano[2,3-*b*]indole]-3′-carbonitrile (**3j**). We followed the general procedure, using **1j** (0.2 mmol, 48.7 mg), **2a** (0.2 mmol, 39.1 mg), and **L4** (0.02 mmol, 19.0 mg). Purified by flash column chromatography (petroleum ether/ethyl acetate 2:1) to afford **3j** as a yellow solid (39.4 mg, 45% yield); [α]_D_^20^ = −1.30 (c = 1.0, EA, 87% ee); m.p. = 295.6–296.3 °C; ^1^H NMR (400 MHz, DMSO-d_6_) δ 10.80 (s, 1H), 7.44 (d, *J* = 8.8 Hz, 1H), 7.35 − 7.29 (m, 1H), 7.25 (s, 2H), 7.20 − 7.16 (m, 1H), 7.15 − 7.01 (m, 2H), 7.01 − 6.96 (m, 1H), 6.41 (d, *J* = 1.9 Hz, 1H), 3.71 (s, 3H); ^13^C NMR (101 MHz, DMSO-d_6_) δ 178.0, 152.7, 141.9, 136.7, 133.6, 129.8, 127.5, 125.9, 125.5, 124.2, 123.2, 119.3, 117.8, 112.9, 112.2, 110.3, 103.0, 72.05, 52.0, 31.0; HRMS (ESI): *m*/*z* [M + Na]^+^ calcd for [C_20_H_13_BrN_4_OSNa]^+^: 458.9886, found: 458.9889; HPLC: Daicel Chiralpak IA, n-hexane/i-PrOH = 70/30, flow rate = 1 mL/min, λ = 254 nm, t_major_ = 11.73 min, and t_minor_ = 38.58 min.

(*R*)-2′-amino-7′-chloro-9′-methyl-2-oxo-9′*H*-spiro[indoline-3,4′-thiopyrano[2,3-*b*]indole]-3′-carbonitrile (**3k**). We followed the general procedure, using **1k** (0.2 mmol, 39.3 mg), **2a** (0.2 mmol, 39.1 mg), and **L4** (0.02 mmol, 19.0 mg). Purified by flash column chromatography (petroleum ether/ethyl acetate 2:1) to afford **3k** as a yellow solid (43.2 mg, 55% yield); [α]_D_^20^ = −6.54 (c = 1.0, EA, 97% ee); m.p. = 274.5–275.3 °C; ^1^H NMR (400 MHz, DMSO-d_6_) δ 10.77 (s, 1H), 7.62 (d, *J* = 1.9 Hz, 1H), 7.30 (m, 1H), 7.22 (s, 2H), 7.07 − 6.99 (m, 2H), 6.99 − 6.93 (m, 1H), 6.89 − 6.80 (m, 1H), 6.30 (d, *J* = 8.6 Hz, 1H), 3.71 (s, 3H); ^13^C NMR (101 MHz, DMSO-d_6_) δ 178.1, 152.6, 141.9, 138.3, 133.8, 129.7, 126.8, 126.7, 125.5, 123.1, 120.6, 118.2, 117.8, 110.4, 110.2, 103.6, 72.2, 52.1, 30.9; HRMS (ESI): *m*/*z* [M + H]^+^ calcd for [C_20_H_14_ClN_4_OS]^+^: 393.0571, found: 393.0578; HPLC: Daicel Chiralpak IA, n-hexane/i-PrOH = 70/30, flow rate = 1 mL/min, λ = 254 nm, t_major_ = 12.03 min, and t_minor_ = 28.12 min.

(*R*)-2′-amino-7′-bromo-9′-methyl-2-oxo-9′*H*-spiro[indoline-3,4′-thiopyrano[2,3-*b*]indole]-3′-carbonitrile (**3l**). We followed the general procedure, using **1l** (0.2 mmol, 48.6 mg), **2a** (0.2 mmol, 39.1 mg), and **L4** (0.02 mmol, 19.0 mg). Purified by flash column chromatography (petroleum ether/ethyl acetate 2:1) to afford **3l** as a yellow solid (52.5 mg, 60% yield); [α]_D_^20^ = −4.35 (c = 1.0, EA, 97% ee); m.p. = 297.7–298.3 °C; ^1^H NMR (400 MHz, DMSO-d_6_) δ 10.77 (s, 1H), 7.75 (s, 1H), 7.30 (t, *J* = 7.6 Hz, 1H), 7.22 (s, 2H), 7.10 − 6.77 (m, 4H), 6.25 (d, *J* = 8.6 Hz, 1H), 3.71 (s, 3H); ^13^C NMR (101 MHz, DMSO-d_6_) δ 178.0, 152.6, 141.9, 138.7, 133.8, 129.7, 126.7, 125.4, 123.3, 123.2, 123.1, 118.6, 117.8, 114.8, 113.0, 110.4, 103.6, 72.2, 52.1, 30.9; HRMS (ESI): *m*/*z* [M + H]^+^ calcd for [C_20_H_14_BrN_4_OS]^+^: 437.0066, found: 437.0067; HPLC: Daicel Chiralpak IA, n-hexane/i-PrOH = 70/30, flow rate = 1 mL/min, λ = 254 nm, t_major_ = 13.08 min, and t_minor_ = 30.39 min.

(*R*)-2′-amino-8′-fluoro-9′-methyl-2-oxo-9′*H*-spiro[indoline-3,4′-thiopyrano[2,3-*b*]indole]-3′-carbonitrile (**3m**). We followed the general procedure, using **1m** (0.2 mmol, 36.4 mg), **2a** (0.2 mmol, 39.1 mg), and **L4** (0.02 mmol, 19.1 mg). Purified by flash column chromatography (petroleum ether/ethyl acetate 2:1) to afford **3m** as a yellow solid (37.6 mg, 50% yield); [α]_D_^20^ = −8.52 (c = 1.0, EA, 99% ee); m.p. = 283.4–284.1 °C; ^1^H NMR (400 MHz, DMSO-d_6_) δ 10.78 (s, 1H), 7.33 − 7.27 (m, 1H), 7.23 (s, 2H), 7.01 (m, 2H), 6.96 (t, *J* = 7.5 Hz, 1H), 6.91 − 6.82 (m, 1H), 6.80 − 6.69 (m, 1H), 6.16 (d, *J* = 7.9 Hz, 1H), 3.87 (s, 3H); ^13^C NMR (101 MHz, DMSO-d_6_) δ 178.0, 152.4, 149.4 (d, *J* = 242.9 Hz), 142.0, 133.8, 129.7, 128.3 (d, *J* = 5.1 Hz), 127.4, 125.5, 125.3 (d, *J* = 9.4 Hz), 123.1, 121.0 (d, *J* = 6.6 Hz), 117.8, 113.5, 110.4, 107.8 (d, *J* = 17.7 Hz), 104.4, 72.3, 52.3, 33.5 (d, *J* = 6.2 Hz); ^19^F NMR (376 MHz, DMSO-d_6_) δ -135.58; HRMS (ESI): *m*/*z* [M + H]^+^ calcd for [C_20_H_14_FN_4_OS]^+^: 377.0867, found: 377.0872; HPLC: Daicel Chiralpak IA, n-hexane/i-PrOH = 70/30, flow rate = 1 mL/min, λ = 254 nm, t_major_ = 10.05 min, and t_minor_ = 28.76 min.

(*R*)-2′-amino-8′-bromo-9′-methyl-2-oxo-9′*H*-spiro[indoline-3,4′-thiopyrano[2,3-*b*]indole]-3′-carbonitrile (**3n**). We followed the general procedure, using **1n** (0.2 mmol, 48.5 mg), **2a** (0.2 mmol, 39.1 mg), and **L4** (0.02 mmol, 19.1 mg). Purified by flash column chromatography (petroleum ether/ethyl acetate 2:1) to afford **3n** as a yellow solid (36.6 mg, 42% yield); [α]_D_^20^ = −2.61 (c = 1.0, EA, 85% ee); m.p. = 287.3–288.1 °C; ^1^H NMR (400 MHz, DMSO-d_6_) δ 10.79 (s, 1H), 7.33 − 7.25 (m, 1H), 7.26 − 7.04 (m, 3H), 7.16 − 6.98 (m, 2H), 6.98 − 6.91 (m, 1H), 6.71 (t, *J* = 7.8 Hz, 1H), 6.56 − 6.18 (m, 1H), 4.03 (s, 3H); ^13^C NMR (101 MHz, DMSO-d_6_) δ 177.9, 152.2, 141.9, 134.1, 133.7, 129.7, 128.6, 127.7, 127.1, 125.4, 123.1, 121.9, 117.7, 116.8, 110.4, 103.8, 103.2, 72.5, 52.1, 34.1; HRMS (ESI): *m*/*z* [M + H]^+^ calcd for [C_20_H_14_BrN_4_OS]^+^: 437.0066, found: 437.0071; HPLC: Daicel Chiralpak IA, n-hexane/i-PrOH = 70/30, flow rate = 1 mL/min, λ = 254 nm, t_major_ = 12.63 min, and t_minor_ = 38.47 min.

(*R*)-2′-amino-6′,8′,9′-trimethyl-2-oxo-9′*H*-spiro[indoline-3,4′-thiopyrano[2,3-*b*]indole]-3′-carbonitrile (**3o**). We followed the general procedure, using **1o** (0.2 mmol, 38.5 mg), **2a** (0.2 mmol, 39.1 mg), and **L4** (0.02 mmol, 19.1 mg). Purified by flash column chromatography (petroleum ether/ethyl acetate 2:1) to afford **3o** as a yellow solid (52.5 mg, 68% yield); [α]_D_^20^ = −20.38 (c = 1.0, EA, 99% ee); m.p. = 277.6–278.3 °C; ^1^H NMR (400 MHz, DMSO-d_6_) δ 10.70 (s, 1H), 7.43 − 7.20 (m, 1H), 7.10 (s, 2H), 7.07 − 6.84 (m, 3H), 6.57 (s, 1H), 6.01 (s, 1H), 3.87 (s, 3H), 2.61 (s, 3H), 2.02 (s, 3H); ^13^C NMR (101 MHz, DMSO-d_6_) δ 178.3, 152.6, 141.9, 135.2, 134.3, 129.4, 128.7, 126.3, 125.8, 125.6, 125.3, 122.9, 121.1, 118.0, 115.1, 110.1, 102.8, 72.7, 52.4, 34.0, 21.4, 19.8; HRMS (ESI): *m*/*z* [M + H]^+^ calcd for [C_22_H_19_N_4_OS]^+^: 387.1274, found: 387.1280; HPLC: Daicel Chiralpak IA, n-hexane/i-PrOH = 70/30, flow rate = 1 mL/min, λ = 254 nm, t_major_ = 12.83 min, and t_minor_ = 41.52 min.

(*R*)-2′-amino-5-methoxy-9′-methyl-2-oxo-9′*H*-spiro[indoline-3,4′-thiopyrano[2,3-*b*]indole]-3′-carbonitrile (**3p**). We followed the general procedure, using **1a** (0.2 mmol, 32.6 mg), **2b** (0.2 mmol, 45.3 mg), and **L4** (0.02 mmol, 19.0 mg). Purified by flash column chromatography (petroleum ether/ethyl acetate 2:1) to afford **3p** as a yellow solid (58.2 mg, 75% yield); [α]_D_^20^ = +2.31 (c = 1.0, EA, 99% ee); m.p. = 263.5–264.4 °C; ^1^H NMR (400 MHz, DMSO-d_6_) δ 10.56 (s, 1H), 7.44 (d, *J* = 8.3 Hz, 1H), 7.18 (s, 2H), 7.06 (t, *J* = 7.7 Hz, 1H), 6.94 (d, *J* = 8.4 Hz, 1H), 6.89 − 6.79 (m, 2H), 6.60 (d, *J* = 2.5 Hz, 1H), 6.41 (d, *J* = 8.0 Hz, 1H), 3.71 (s, 3H), 3.61 (s, 3H); ^13^C NMR (101 MHz, DMSO-d_6_) δ 178.1, 155.9, 152.8, 137.9, 135.4, 135.3, 125.3, 124.4, 121.8, 120.3, 118.0, 117.2, 114.4, 111.8, 110.8, 110.0, 103.2, 72.4, 55.8, 52.8, 30.7; HRMS (ESI): *m*/*z* [M + H]^+^ calcd for [C_21_H_17_N_4_O_2_S]^+^: 389.1067, found: 389.1073; HPLC: Daicel Chiralpak IA, n-hexane/i-PrOH = 70/30, flow rate = 1 mL/min, λ = 254 nm, t_major_ = 14.28 min, and t_minor_ = 38.69 min.

(*R*)-2′-amino-5,9′-dimethyl-2-oxo-9′*H*-spiro[indoline-3,4′-thiopyrano[2,3-*b*]indole]-3′-carbonitrile (**3q**). We followed the general procedure, using **1a** (0.2 mmol, 32.6 mg), **2c** (0.2 mmol, 42.2 mg), and **L4** (0.02 mmol, 19.0 mg). Purified by flash column chromatography (petroleum ether/ethyl acetate 2:1) to afford **3q** as a yellow solid (37.9 mg, 51% yield); [α]_D_^20^ = +5.56 (c = 1.0, EA, 98% ee); m.p. = 260.8–261.4 °C; ^1^H NMR (400 MHz, DMSO-d_6_) δ 10.64 (s, 1H), 7.43 (d, *J* = 8.3 Hz, 1H), 7.15 (s, 2H), 7.10 − 7.01 (m, 2H), 6.89 (d, *J* = 7.8 Hz, 1H), 6.86 − 6.72 (m, 2H), 6.44 (d, *J* = 8.0 Hz, 1H), 3.70 (s, 3H), 2.16 (s, 3H); ^13^C NMR (101 MHz, DMSO-d_6_) δ 178.2, 152.5, 139.4, 137.9, 134.4, 131.8, 129.8, 125.8, 125.1, 124.4, 121.8, 120.3, 118.0, 117.2, 110.0, 109.9, 103.3, 72.6, 52.4, 30.7, 21.0; HRMS (ESI): *m*/*z* [M + Na]^+^ calcd for [C_21_H_16_N_4_OSNa]^+^: 395.0937, found: 395.0940; HPLC: Daicel Chiralpak IA, n-hexane/i-PrOH = 80/20, flow rate = 1 mL/min, λ = 254 nm, t_major_ = 21.84 min, and t_minor_ = 62.98 min.

(*R*)-2′-amino-5-bromo-9′-methyl-2-oxo-9′*H*-spiro[indoline-3,4′-thiopyrano[2,3-*b*]indole]-3′-carbonitrile (**3r**). We followed the general procedure, using **1a** (0.2 mmol, 32.6 mg), **2d** (0.2 mmol, 55.3 mg), and **L4** (0.02 mmol, 19.0 mg). Purified by flash column chromatography (petroleum ether/ethyl acetate 2:1) to afford **3r** as a yellow solid (43.7 mg, 50% yield); [α]_D_^20^ = +0.87 (c = 1.0, EA, 90% ee); m.p. = 260.8–261.4 °C; ^1^H NMR (400 MHz, DMSO-d_6_) δ 10.92 (s, 1H), 7.52 − 7.43 (m, 2H), 7.27 (s, 2H), 7.15 (d, *J* = 2.1 Hz, 1H), 7.11 − 7.05 (m, 1H), 6.99 (d, *J* = 8.3 Hz, 1H), 6.88 − 6.82 (m, 1H), 6.43 (d, *J* = 8.0 Hz, 1H), 3.71 (s, 3H); ^13^C NMR (101 MHz, DMSO-d_6_) δ 177.9, 153.1, 141.3, 137.9, 136.6, 132.4, 128.0, 125.6, 124.2, 122.0, 120.5, 117.9, 116.8, 114.6, 112.4, 110.1, 102.4, 71.5, 52.5, 30.7; HRMS (ESI): *m*/*z* [M + H]^+^ calcd for [C_20_H_14_BrN_4_OS]^+^: 437.0066, found: 437.0072; HPLC: Daicel Chiralpak IA, n-hexane/i-PrOH = 70/30, flow rate = 1 mL/min, λ = 254 nm, t_major_ = 10.39 min, and t_minor_ = 22.66 min.

(*R*)-2′-amino-6-methoxy-9′-methyl-2-oxo-9′*H*-spiro[indoline-3,4′-thiopyrano[2,3-*b*]indole]-3′-carbonitrile (**3s**). We followed the general procedure, using **1a** (0.2 mmol, 32.6 mg), **2e** (0.2 mmol, 45.2 mg), and **L4** (0.02 mmol, 19.0 mg). Purified by flash column chromatography (petroleum ether/ethyl acetate 2:1) to afford **3s** as a yellow solid (66 mg, 85% yield); [α]_D_^20^ = +3.08 (c = 1.0, EA, 99% ee); m.p. = 243.5–244.3 °C; ^1^H NMR (400 MHz, DMSO-d_6_) δ 10.67 (s, 1H), 7.43 (d, *J* = 8.3 Hz, 1H), 7.12 (s, 2H), 7.08 − 7.01 (m, 1H), 6.91 (d, *J* = 8.2 Hz, 1H), 6.87 − 6.76 (m, 1H), 6.55 (d, *J* = 2.3 Hz, 1H), 6.52 − 6.44 (m, 1H), 6.39 (d, *J* = 8.0 Hz, 1H), 3.77 (s, 3H), 3.69 (s, 3H); ^13^C NMR (101 MHz, DMSO-d_6_) δ 178.8, 160.5, 152.5, 143.2, 137.9, 126.2, 126.0, 125.2, 124.5, 121.8, 120.3, 118.0, 117.3, 109.9, 107.8, 103.6, 96.9, 72.8, 55.7, 51.8, 30.7; HRMS (ESI): *m*/*z* [M + H]^+^ calcd for [C_21_H_17_N_4_O_2_S]^+^: 389.1067, found: 389.1067; HPLC: Daicel Chiralpak IA, n-hexane/i-PrOH = 70/30, flow rate = 1 mL/min, λ = 254 nm, t_major_ = 14.13 min, and t_minor_ = 36.74 min.

(*R*)-2′-amino-7,9′-dimethyl-2-oxo-9′*H*-spiro[indoline-3,4′-thiopyrano[2,3-*b*]indole]-3′-carbonitrile (**3t**). We followed the general procedure, using **1a** (0.2 mmol, 32.6 mg), **2f** (0.2 mmol, 42.1 mg), and **L4** (0.02 mmol, 19.0 mg). Purified by flash column chromatography (petroleum ether/ethyl acetate 2:1) to afford **3t** as a yellow solid (65.5 mg, 88% yield); [α]_D_^20^ = +3.33 (c = 1.0, EA, 97% ee); m.p. = 268.4–269.1 °C; ^1^H NMR (400 MHz, DMSO-d_6_) δ 10.78 (s, 1H), 7.43 (d, *J* = 8.3 Hz, 1H), 7.36 − 6.89 (m, 4H), 6.99 − 6.60 (m, 3H), 6.36 (d, *J* = 8.0 Hz, 1H), 3.71 (s, 3H), 2.34 (s, 3H); ^13^C NMR (101 MHz, DMSO-d_6_) δ 178.7, 152.7, 140.6, 137.9, 133.8, 130.7, 125.2, 124.4, 122.9, 122.8, 121.8, 120.3, 119.5, 118.0, 117.3, 109.9, 103.5, 72.6, 52.5, 30.7, 17.0; HRMS (ESI): *m*/*z* [M + H]^+^ calcd for [C_21_H_17_N_4_OS]^+^: 373.1118, found: 373.1121; HPLC: Daicel Chiralpak IA, n-hexane/i-PrOH = 70/30, flow rate = 1 mL/min, λ = 254 nm, t_major_ = 11.65 min, and t_minor_ = 22.90 min.

(*R*)-2′-amino-7-fluoro-9′-methyl-2-oxo-9′*H*-spiro[indoline-3,4′-thiopyrano[2,3-*b*]indole]-3′-carbonitrile (**3u**). We followed the general procedure, using **1a** (0.2 mmol, 32.6 mg), **2g** (0.2 mmol, 43.3 mg), and **L4** (0.02 mmol, 19.0 mg). Purified by flash column chromatography (petroleum ether/ethyl acetate 2:1) to afford **3u** as a yellow solid (63.2 mg, 84% yield); [α]_D_^20^ = +6.30 (c = 1.0, EA, 92% ee); m.p. = 268.4–269.1 °C; ^1^H NMR (400 MHz, DMSO-d_6_) δ 11.31 (s, 1H), 7.45 (d, *J* = 8.3 Hz, 1H), 7.32 − 7.16 (m, 3H), 7.11 − 7.04 (m, 1H), 7.02 − 6.94 (m, 1H), 6.92 − 6.81 (m, 2H), 6.40 (d, *J* = 8.0 Hz, 1H), 3.71 (s, 3H); ^13^C NMR (101 MHz, DMSO-d_6_) δ 178.1, 153.0, 146.8 (d, *J* = 243.2 Hz), 137.9, 136.9 (d, *J* = 3.2 Hz), 129.0 (d, *J* = 12.5 Hz), 125.4, 124.2, 124.0 (d, *J* = 5.7 Hz), 122.0, 121.5, 120.5, 117.9, 116.9, 116.5 (d, *J* = 17.0 Hz), 110.1, 102.6, 71.8, 52.6 (d, *J* = 2.8 Hz), 30.7; ^19^F NMR (376 MHz, DMSO-d_6_) δ -132.63; HRMS (ESI): *m*/*z* [M + Na]^+^ calcd for [C_20_H_13_FN_4_OSNa]^+^: 399.0686, found: 399.0684; HPLC: Daicel Chiralpak IA, n-hexane/i-PrOH = 70/30, flow rate = 1 mL/min, λ = 254 nm, t_major_ = 11.72 min, and t_minor_ = 38.81 min.

(*R*)-2′-amino-7-chloro-9′-methyl-2-oxo-9′*H*-spiro[indoline-3,4′-thiopyrano[2,3-*b*]indole]-3′-carbonitrile (**3v**). We followed the general procedure, using **1a** (0.2 mmol, 32.6 mg), **2h** (0.2 mmol, 46.1 mg), and **L4** (0.02 mmol, 19.0 mg). Purified by flash column chromatography (petroleum ether/ethyl acetate 2:1) to afford **3v** as a yellow solid (64.3 mg, 82% yield); [α]_D_^20^ = +8.85 (c = 1.0, EA, 94% ee); m.p. = 265.3–265.9 °C; ^1^H NMR (400 MHz, DMSO-d_6_) δ 11.23 (s, 1H), 7.46 (d, *J* = 8.3 Hz, 1H), 7.41 − 7.33 (m, 1H), 7.29 (s, 2H), 7.13 − 7.03 (m, 1H), 7.05 − 6.91 (m, 2H), 6.93 − 6.80 (m, 1H), 6.38 (d, *J* = 7.9 Hz, 1H), 3.71 (s, 3H); ^13^C NMR (101 MHz, DMSO-d_6_) δ 178.3, 153.1, 139.7, 137.9, 135.9, 129.5, 125.5, 124.4, 124.20, 124.16, 122.0, 120.5, 117.9, 116.8, 114.6, 110.1, 102.6, 71.7, 53.2, 30.7; HRMS (ESI): *m*/*z* [M + H]^+^ calcd for [C_20_H_14_ClN_4_OS]^+^: 393.0571, found: 393.0569; HPLC: Daicel Chiralpak IA, n-hexane/i-PrOH = 70/30, flow rate = 1 mL/min, λ = 254 nm, t_major_ = 13.00 min, and t_minor_ = 37.30 min.

(*R*)-2′-amino-7-bromo-9′-methyl-2-oxo-9′*H*-spiro[indoline-3,4′-thiopyrano[2,3-*b*]indole]-3′-carbonitrile (**3w**). Followed the general procedure, using **1a** (0.2 mmol, 32.6 mg), **2i** (0.2 mmol, 55.2 mg), and **L4** (0.02 mmol, 19.0 mg). Purified by flash column chromatography (petroleum ether/ethyl acetate 2:1) to afford **3w** as a yellow solid (69 mg, 79% yield); [α]_D_^20^ = +3.91 (c = 1.0, EA, 94% ee); m.p. = 264.9–265.7 °C; ^1^H NMR (400 MHz, DMSO-d_6_) δ 11.09 (s, 1H), 7.63 − 7.34 (m, 2H), 7.28 (s, 2H), 7.20 − 6.96 (m, 2H), 7.00 − 6.72 (m, 2H), 6.37 (d, *J* = 8.0 Hz, 1H), 3.72 (s, 3H); ^13^C NMR (101 MHz, DMSO-d_6_) δ 178.2, 153.1, 141.4, 137.9, 135.8, 132.4, 125.4, 124.8, 124.6, 124.2, 122.0, 120.5, 117.9, 116.8, 110.1, 102.7, 102.6, 71.7, 53.4, 30.8; HRMS (ESI): *m*/*z* [M + H]^+^ calcd for [C_20_H_14_BrN_4_OS]^+^: 437.0066, found: 437.0076; HPLC: Daicel Chiralpak IA, n-hexane/i-PrOH = 70/30, flow rate = 1 mL/min, λ = 254 nm, t_major_ = 15.01 min, and t_minor_ = 39.45 min.

(*R*)-2′-amino-5,7,9′-trimethyl-2-oxo-9′*H*-spiro[indoline-3,4′-thiopyrano[2,3-*b*]indole]-3′-carbonitrile (**3x**). Followed the general procedure, using **1a** (0.2 mmol, 32.6 mg), **2j** (0.2 mmol, 45.2 mg), and **L4** (0.02 mmol, 19.0 mg). Purified by flash column chromatography (petroleum ether/ethyl acetate 2:1) to afford **3x** as a yellow solid (60.2 mg, 78% yield); [α]_D_^20^ = +6.54 (c = 1.0, EA, 94% ee); m.p. = 251.8–252.5 °C; ^1^H NMR (400 MHz, DMSO-d_6_) δ 10.70 (s, 1H), 7.43 (d, *J* = 8.3 Hz, 1H), 7.33 − 6.95 (m, 3H), 6.90 (d, *J* = 1.8 Hz, 1H), 6.82 (t, *J* = 7.4 Hz, 1H), 6.65 (d, *J* = 1.7 Hz, 1H), 6.45 (d, *J* = 8.0 Hz, 1H), 3.70 (s, 3H), 2.31 (s, 3H), 2.13 (s, 3H); ^13^C NMR (101 MHz, DMSO-d_6_) δ 178.7, 152.4, 138.0, 137.9, 134.1, 131.7, 131.2, 125.0, 124.4, 123.1, 121.8, 120.3, 119.2, 118.1, 117.3, 109.9, 103.6, 72.8, 52.6, 30.7, 20.9, 16.9; HRMS (ESI): *m*/*z* [M + H]^+^ calcd for [C_22_H_19_BrN_4_OS]^+^: 387.1274, found: 387.1274; HPLC: Daicel Chiralpak IA, n-hexane/i-PrOH = 70/30, flow rate = 1 mL/min, λ = 254 nm, t_major_ = 10.11 min, and t_minor_ = 20.57 min.

### 4.4. Procedure for the Scaled-Up Synthesis of Compound ***3a***

Under a nitrogen atmosphere, a solution of diethylzinc (800 μL, 1.0 M in hexane, 0.8 mmol) was added dropwise to a solution of **L4** (0.4 mmol, 0.364 g) in THF (10 mL). After the mixture was stirred for 30 min at room temperature. Then, 1-methylindoline-2-thione **1a** (4.0 mmol, 0.652 g) and 2-(2-oxoindolin-3-ylidene)malononitrile **2a** (4.0 mmol, 0.78 g) were added. The reaction mixture was stirred for 24 h at the same temperature. The reaction was quenched with NH_4_Cl solution (10 mL), and the organic layer was extracted with CH_2_Cl_2_ (3 × 10 mL). The combined organic layer was washed with brine and dried over Na_2_SO_4_. The solvent was removed under reduced pressure by using a rotary evaporator. The residue was purified by flash chromatography with petroleum ether/ethyl acetate (2:1) to afford the desired product **3a** (1.07g) as a yellow solid.

### 4.5. Procedure for the Synthesis of Compound ***4***

Compound **3i** (0.1 mmol, 39.2 mg) and pyridine (0.1 mmol) were added to Ac_2_O (0.5 mL), and the mixture was stirred at 25 °C for 8 h. The reaction was quenched with water, and extracted with CH_2_Cl_2_ (3 × 5 mL). The organic layers were dried over Na_2_SO_4_ and concentrated. Then the solvent was removed under reduced pressure. The residue was purified by flash chromatography on silica gel (petroleum ether/ethyl acetate = 5:1) to provide product **4** as a yellow solid.

7′-chloro-2′,10′-dimethyl-10′*H*-spiro[indoline-3,5′-pyrimido[5′,4′:5,6]thiopyrano[2,3-*b*]indole]-2,4′(3′H)-dione (**4**). Purified by flash column chromatography (petroleum ether/ethyl acetate 5:1) to afford **4** as a white solid (25.2 mg, 58% yield); [α]_D_^20^ = +1.74 (c = 1.0, EA, 99% ee); m.p. = 278.8–279.6 °C; ^1^H NMR (400 MHz, DMSO-d_6_) δ 8.34 (d, J = 8.2 Hz, 1H), 7.64 − 7.52 (m, 4H), 7.37 − 7.27 (m, 2H), 7.19 − 7.10 (m, 1H), 5.97 (d, J = 2.0 Hz, 1H), 3.80 (s, 3H), 2.62 (s, 3H); ^13^C NMR (101 MHz, DMSO-d_6_) δ 177.5, 171.1, 154.0, 139.8, 136.5, 131.5, 130.2, 128.4, 126.7, 125.9, 125.2, 125.1, 121.9, 117.5, 116.4, 116.1, 112.0, 102.6, 71.6, 52.4, 31.2, 26.7; HRMS (ESI): m/z [M + H]^+^ calcd for [C_22_H_16_ClN_4_O_2_S]^+^: 435.0677, found: 435.0684; HPLC: Daicel Chiralpak IA, n-hexane/i-PrOH = 70/30, flow rate = 1 mL/min, λ = 254 nm, t_major_ = 7.31 min, and t_minor_ = 10.17 min.

## Figures and Tables

**Table 1:** Condition Optimization ^a^.

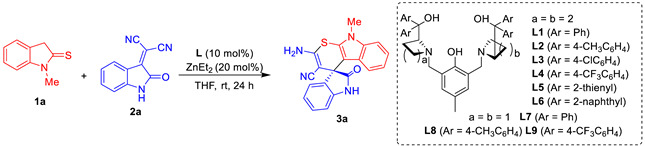			
Entry	Ligand	Yield ^b^	Ee ^c^ (%)
1	**L1**	57	80
2	**L2**	41	63
3	**L3**	78	99
4	**L4**	85	99
5	**L5**	43	34
6	**L6**	57	68
7	**L7**	36	23
8	**L8**	40	37
9	**L9**	72	97

^a^ Unless otherwise noted, all reactions were conducted with 10 mol % of ligand, 20 mol % of ZnEt_2_, 0.10 mmol **1a,** and 0.10 mmol **2a** in 2 mL THF. ^b^ Isolated yields. ^c^ The enantiomeric excess (ee) value was determined by HPLC analysis.

## Data Availability

Not applicable.

## Supplementary Materials

The following supporting information can be downloaded at: https://www.mdpi.com/article/10.3390/molecules28031056/s1, Figures S1–S28: NMR Spectra; Figures S29–S53: HPLC; Figure S54: Single-crystal X-ray diffraction.

## Sample Availability

Samples of the compounds **3** are available from the authors.
