# Supplementary material for: Zinc-Catalyzed Enantioselective [3 + 3] Annulation for Synthesis of Chiral Spiro[indoline-3,4′-thiopyrano[2,3-b]indole] Derivatives
Source: Molecules. 2023 Jan 20;28(3):1056. doi: 10.3390/molecules28031056 (PMC9920981; doi:10.3390/molecules28031056)
Supplement: Supplementary file 1 [file molecules-28-01056-s001.zip › molecules-2132574-supplementary.pdf]

Supporting Information

Zinc-catalyzed enantioselective [3 + 3] annulation for synthesis of chiral spiro[indoline-3,4'-thiopyrano[2,3-*b*]indole] derivatives

Tian-Tian Liu, Yu Chen, Guang-Jian Mei, Yuan-Zhao Hua,* Shi-Kun Jia* and Min-Can Wang*

College of Chemistry and Institute of Green Catalysis, Zhengzhou University, Zhengzhou City, Henan 450000, P. R. China

Email: hyzh@zzu.edu.cn, jiashikun@zzu.edu.cn, wangmincan@zzu.edu.cn

Table of Contents

1. NMR Spectra **S2**

2. HPLC **S29**

3. Single-crystal X-ray diffraction **S54**

NMR Spectra of compounds

¹H NMR (400 MHz, DMSO-d$_6$)

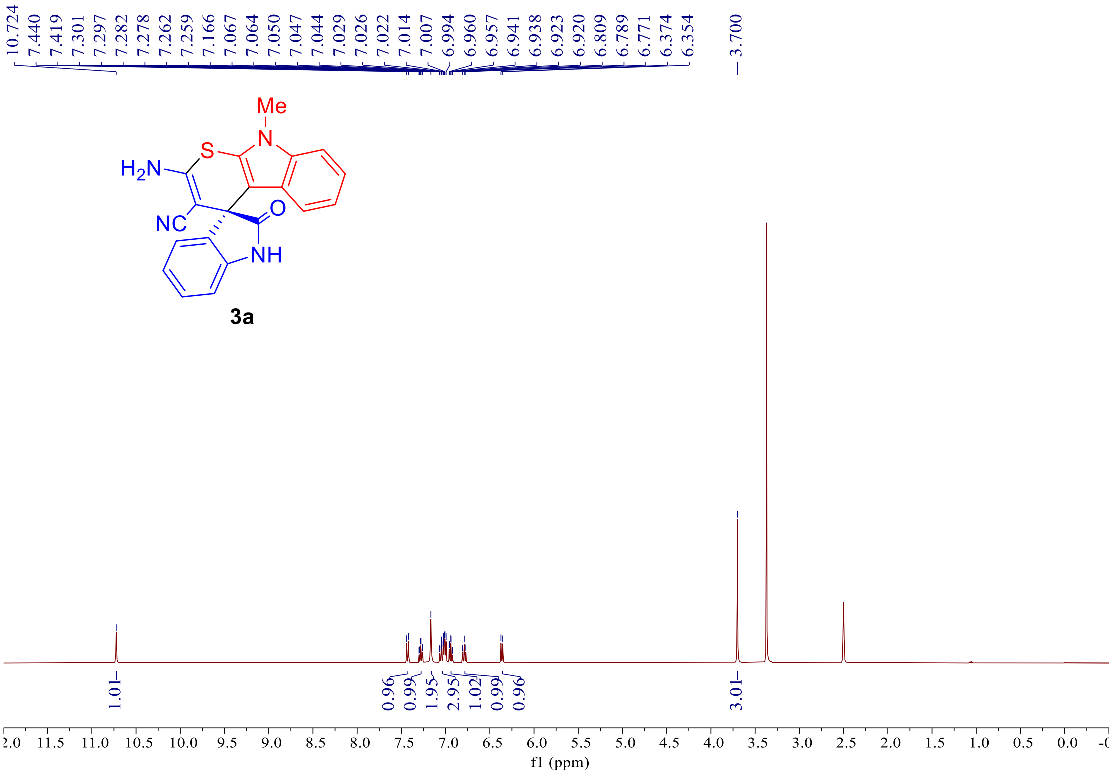

¹³C NMR (101 MHz, DMSO-d$_6$)

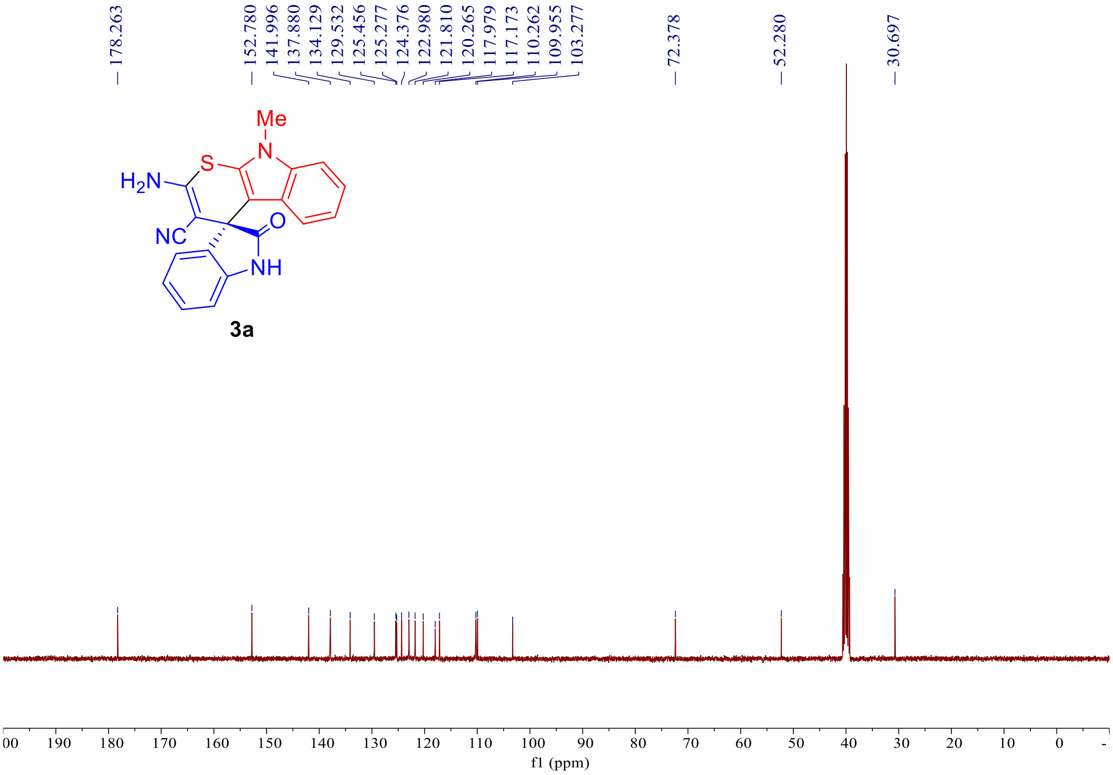

^{1}H NMR (400 MHz, DMSO-d_6)

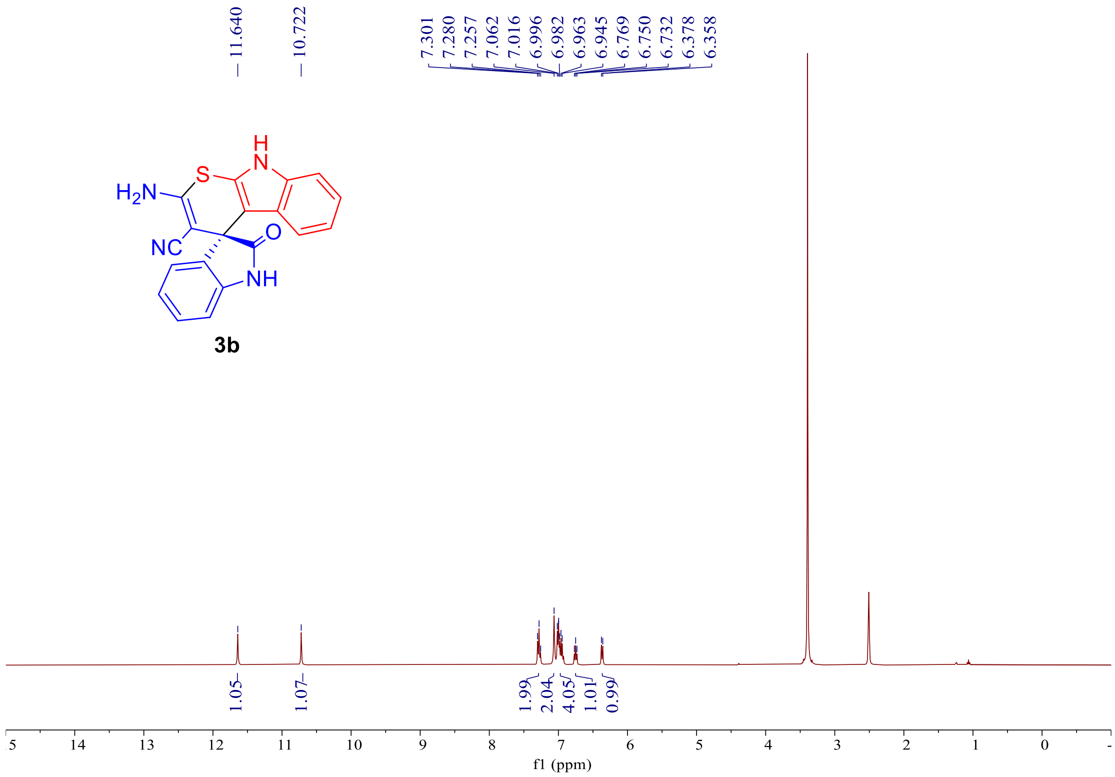

^{13}C NMR (101 MHz, DMSO-d_6)

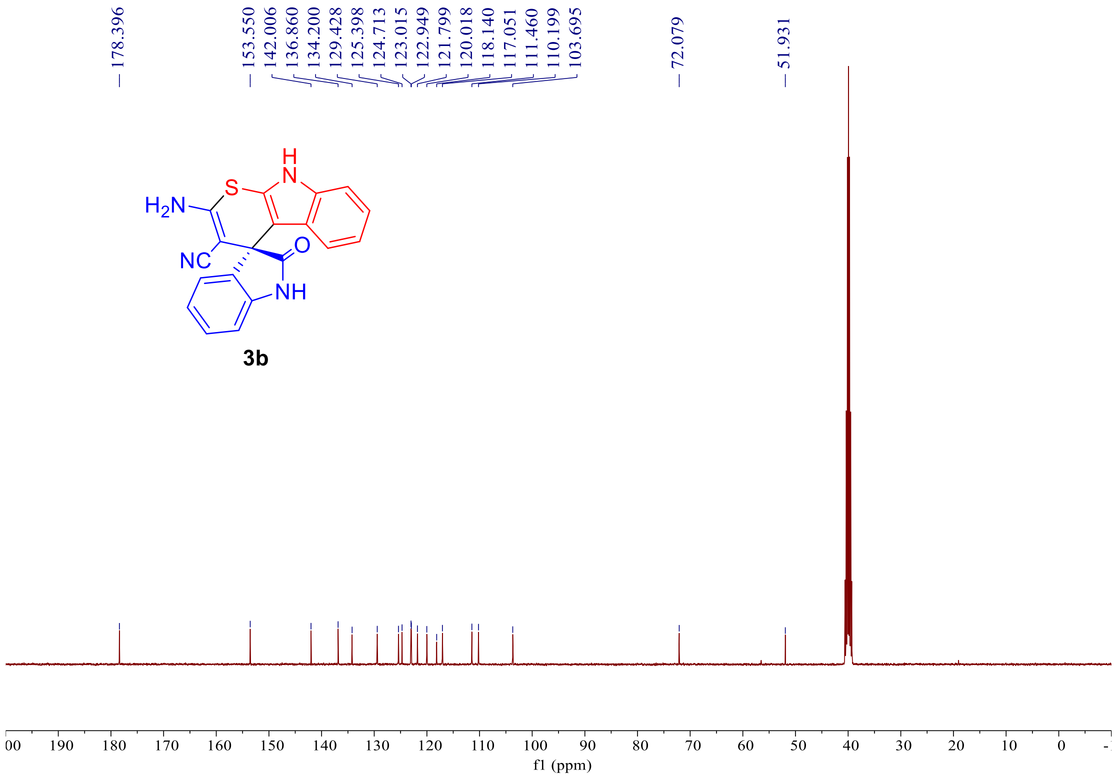

^{1}H NMR (400 MHz, DMSO-d$_6$)

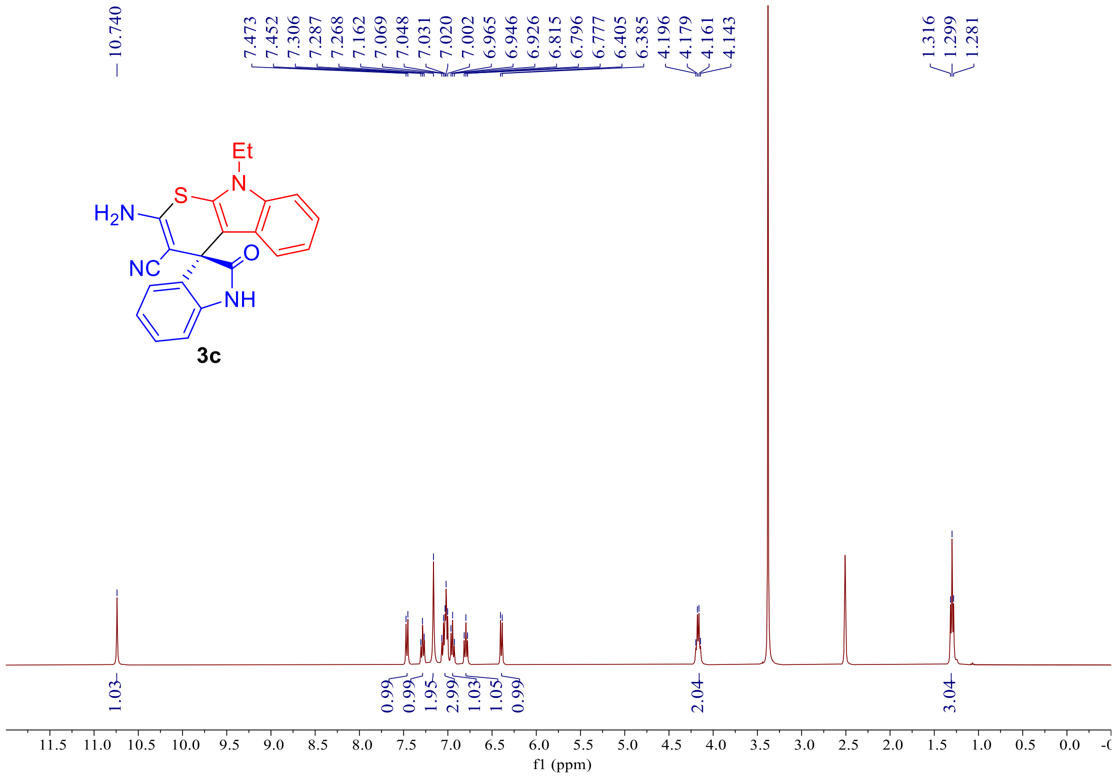

^{13}C NMR (101 MHz, DMSO-d$_6$)

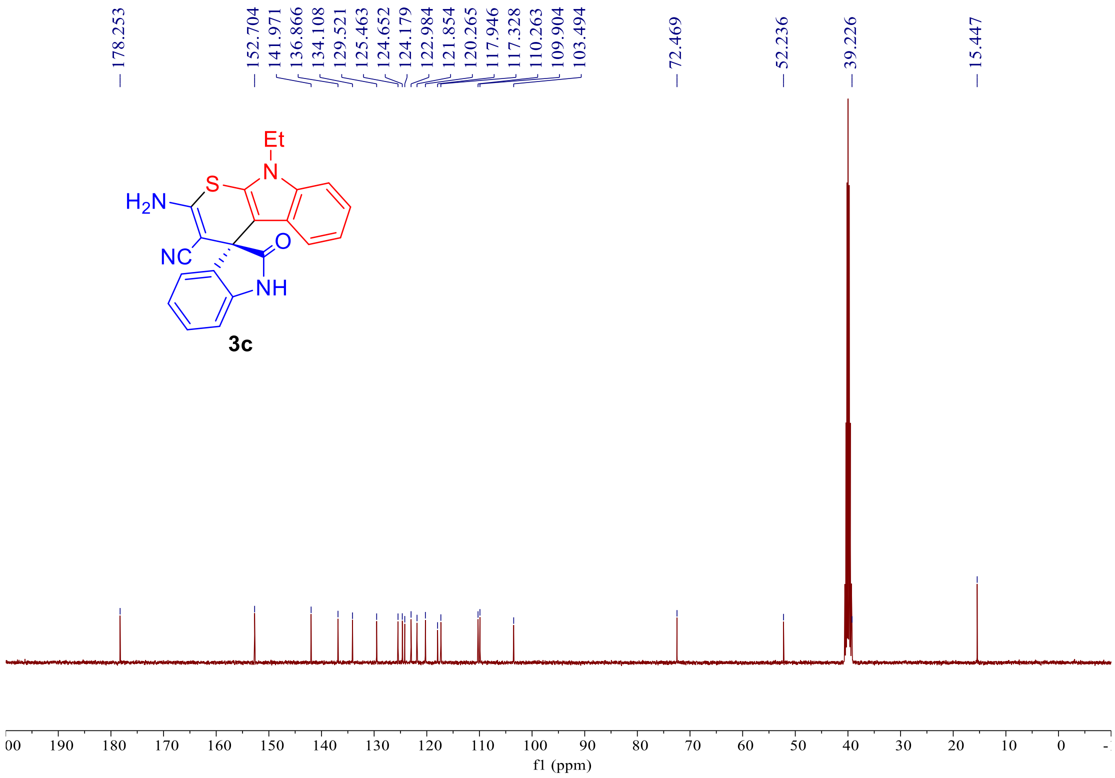

^{1}H NMR (400 MHz, DMSO-d_6)

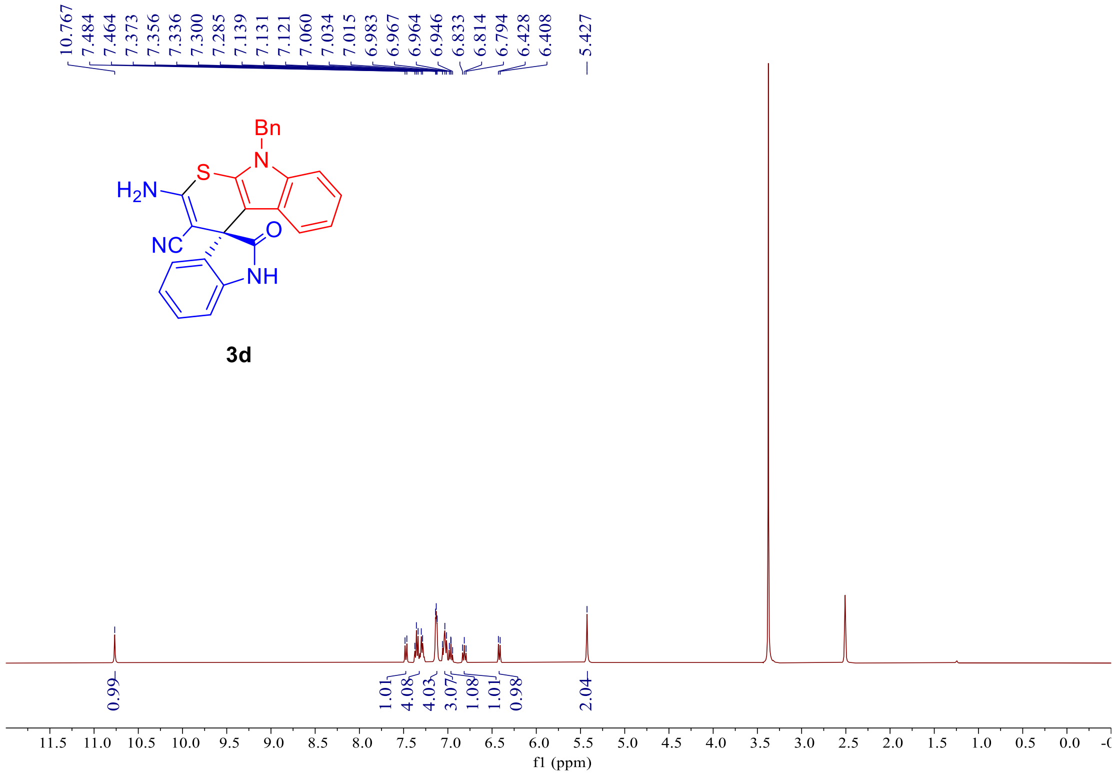

^{13}C NMR (101 MHz, DMSO-d_6)

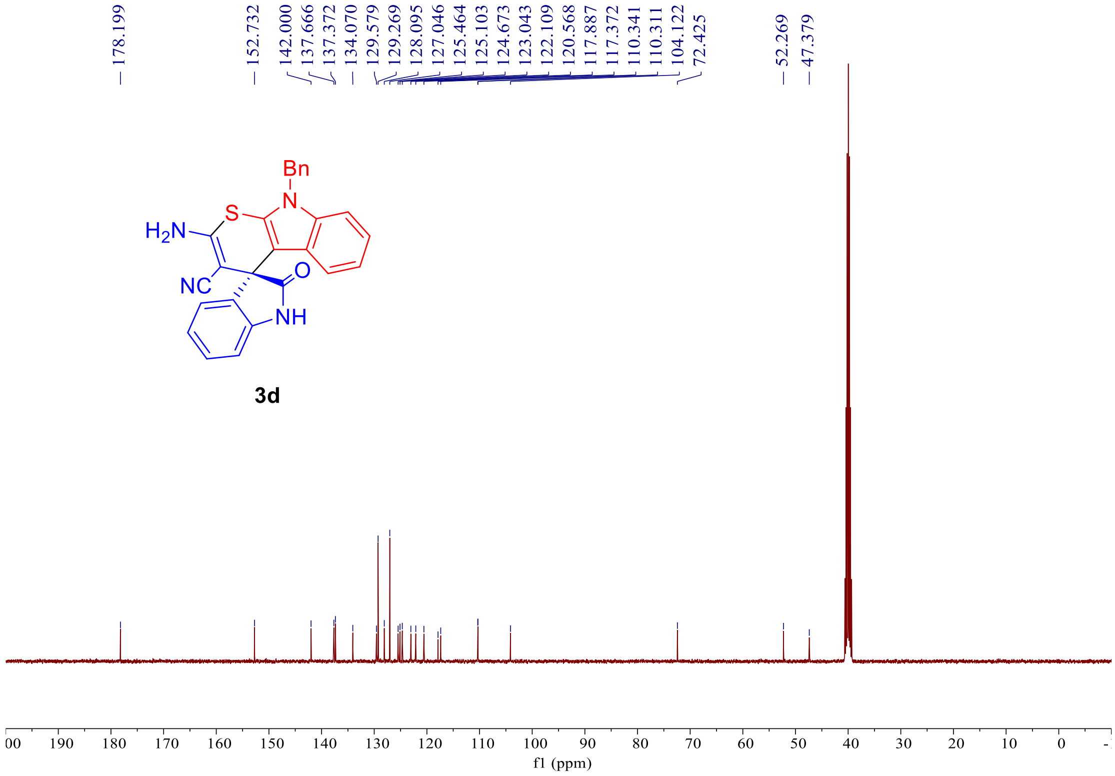

^{1}**H NMR** (400 MHz, DMSO-d$_6$)

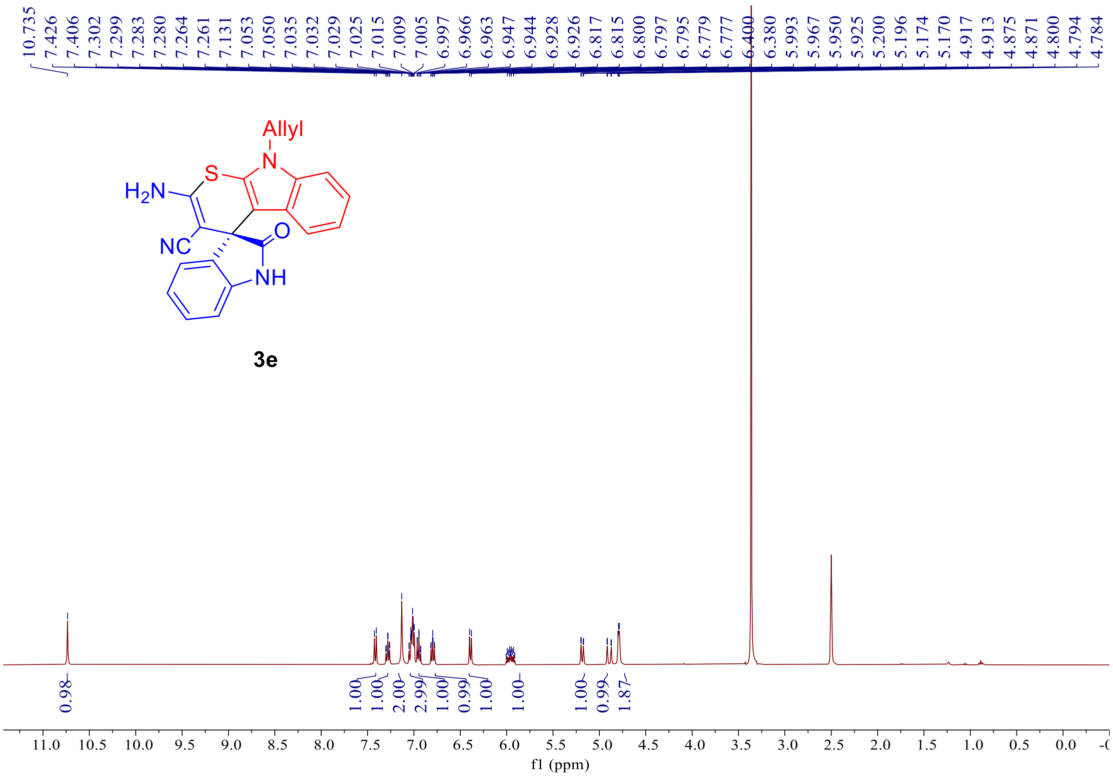

^{13}**C NMR** (101 MHz, DMSO-d$_6$)

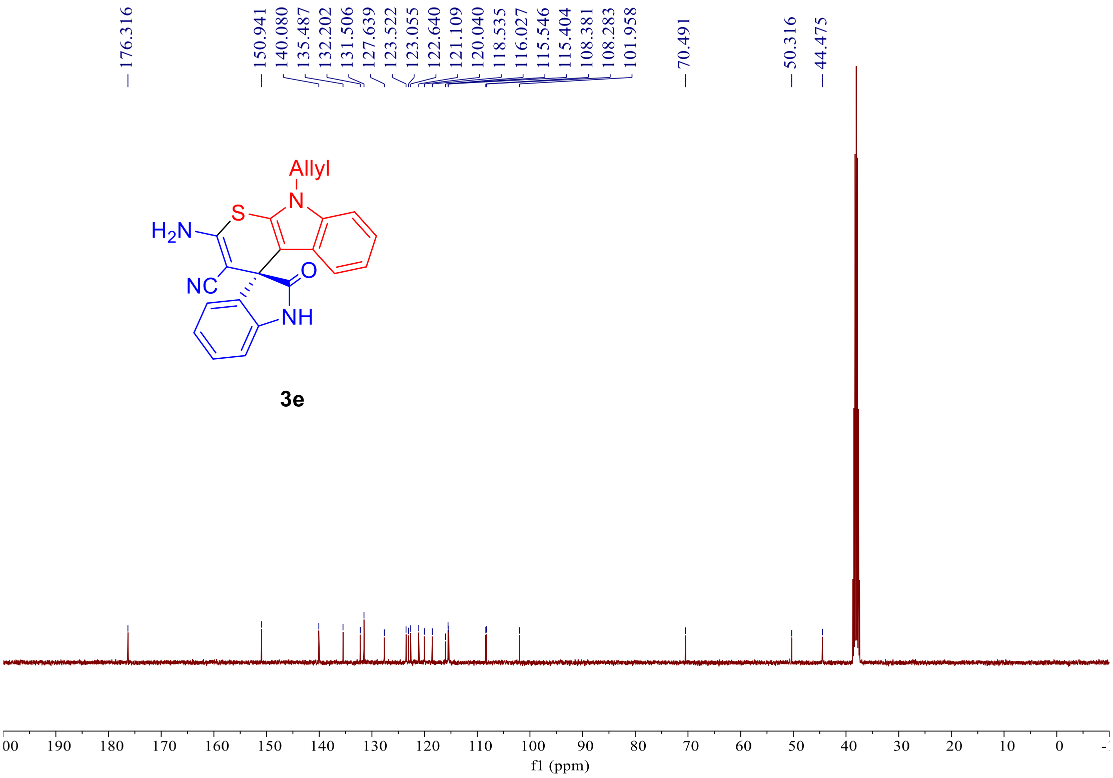

^{1}H NMR (400 MHz, DMSO-d$_6$)

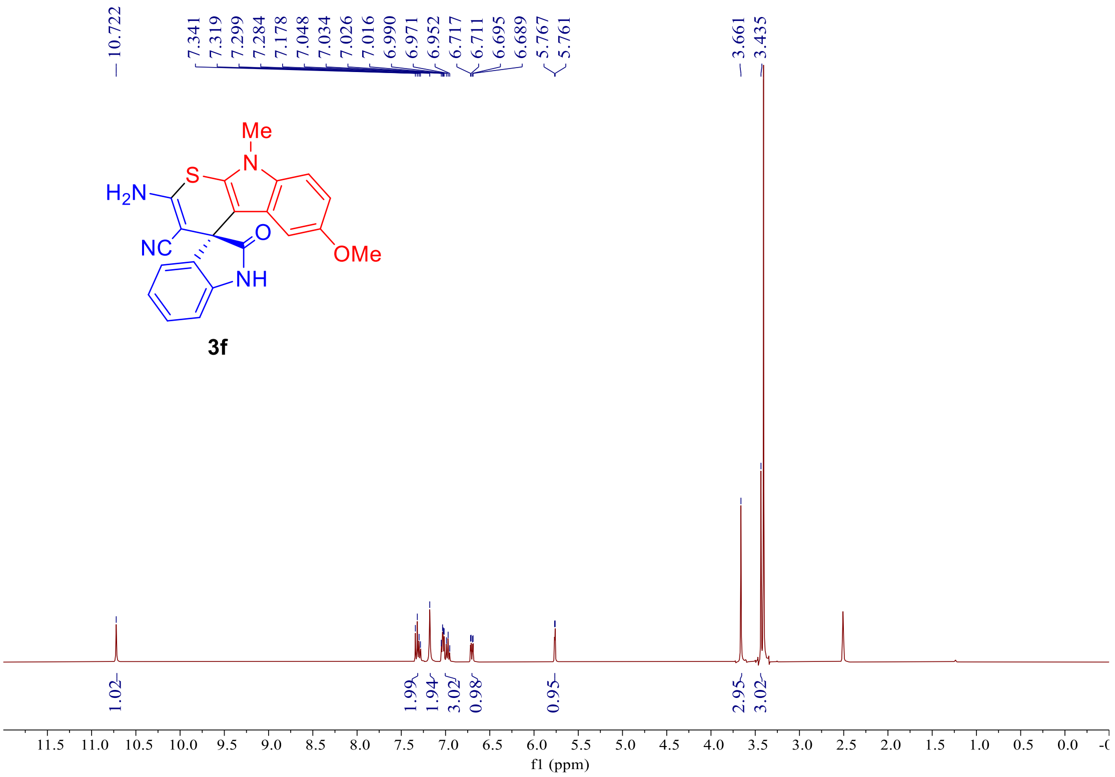

^{13}C NMR (101 MHz, DMSO-d$_6$)

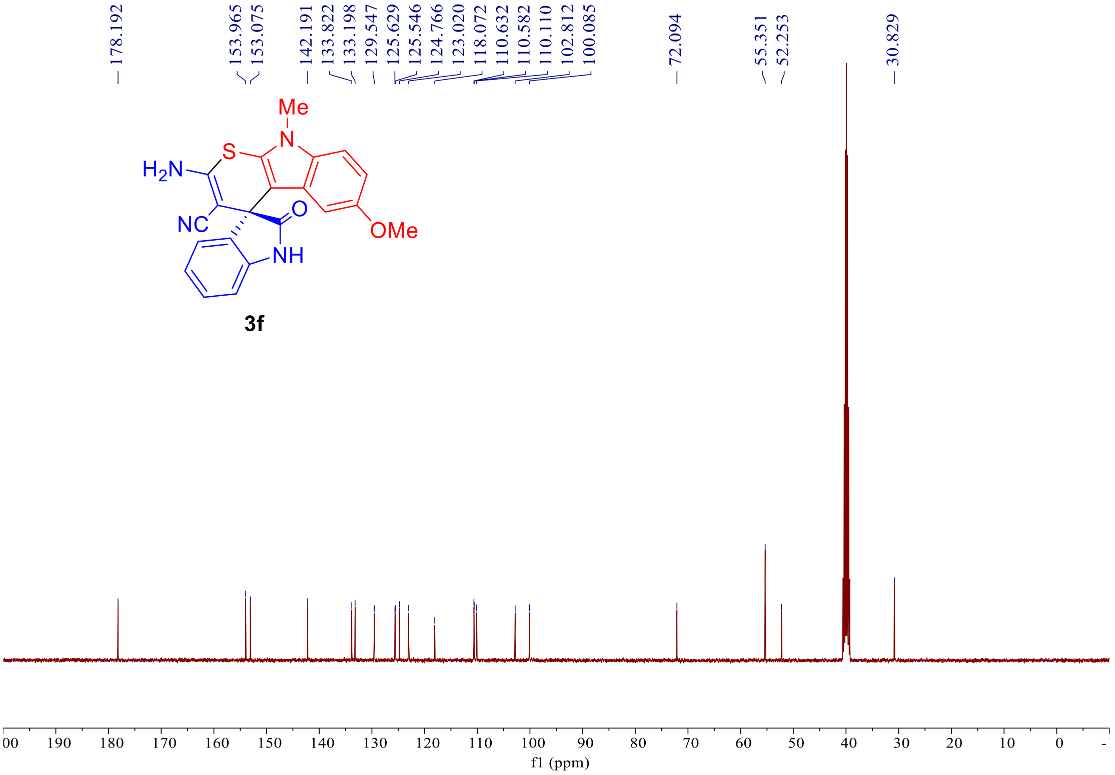

^{1}H NMR (400 MHz, DMSO-d$_6$)

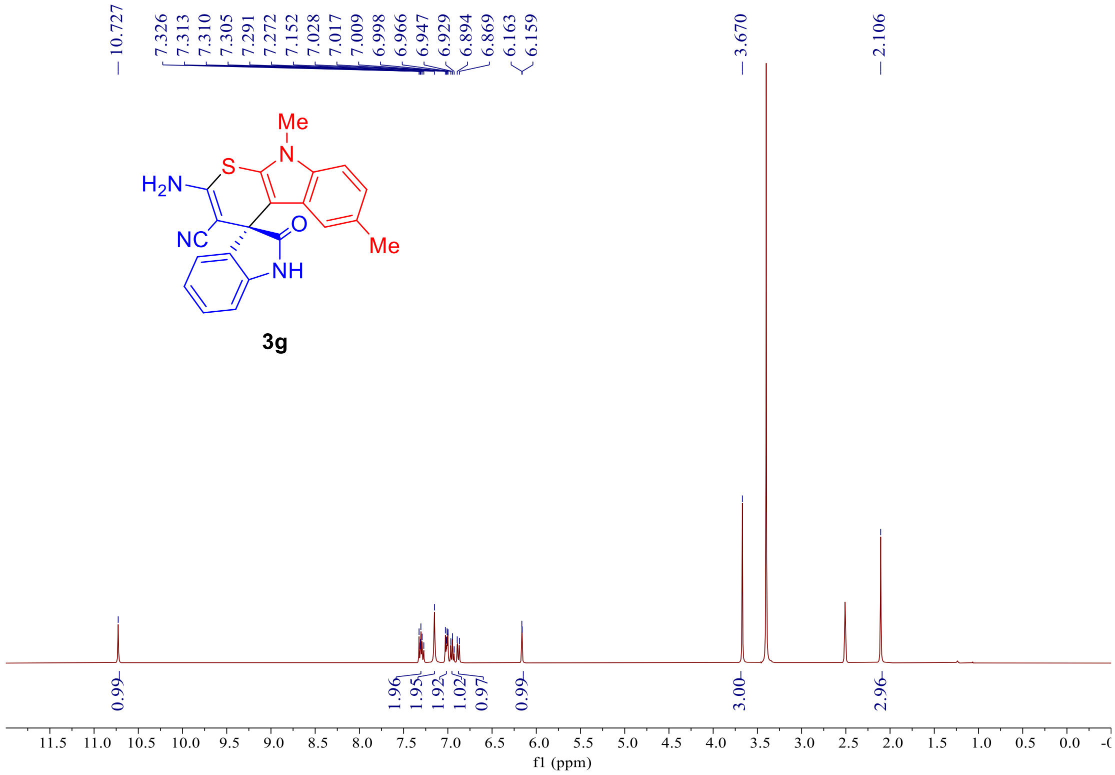

^{13}C NMR (101 MHz, DMSO-d$_6$)

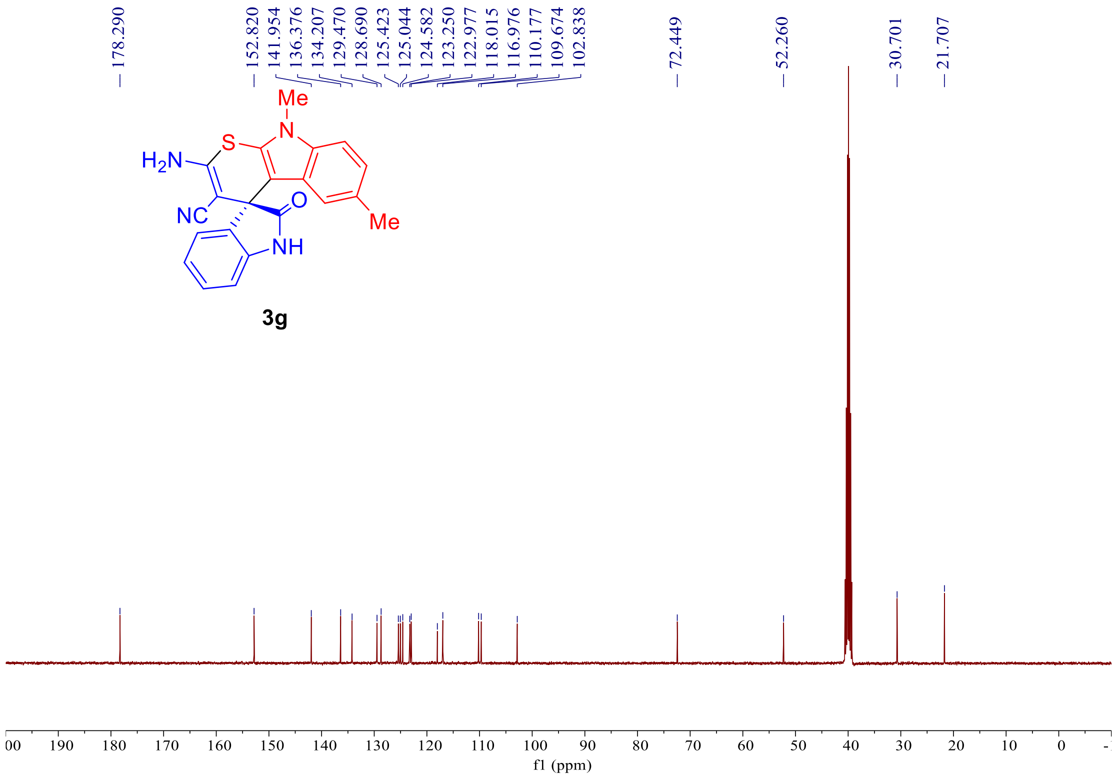

^{1}H NMR (400 MHz, DMSO-d$_6$)

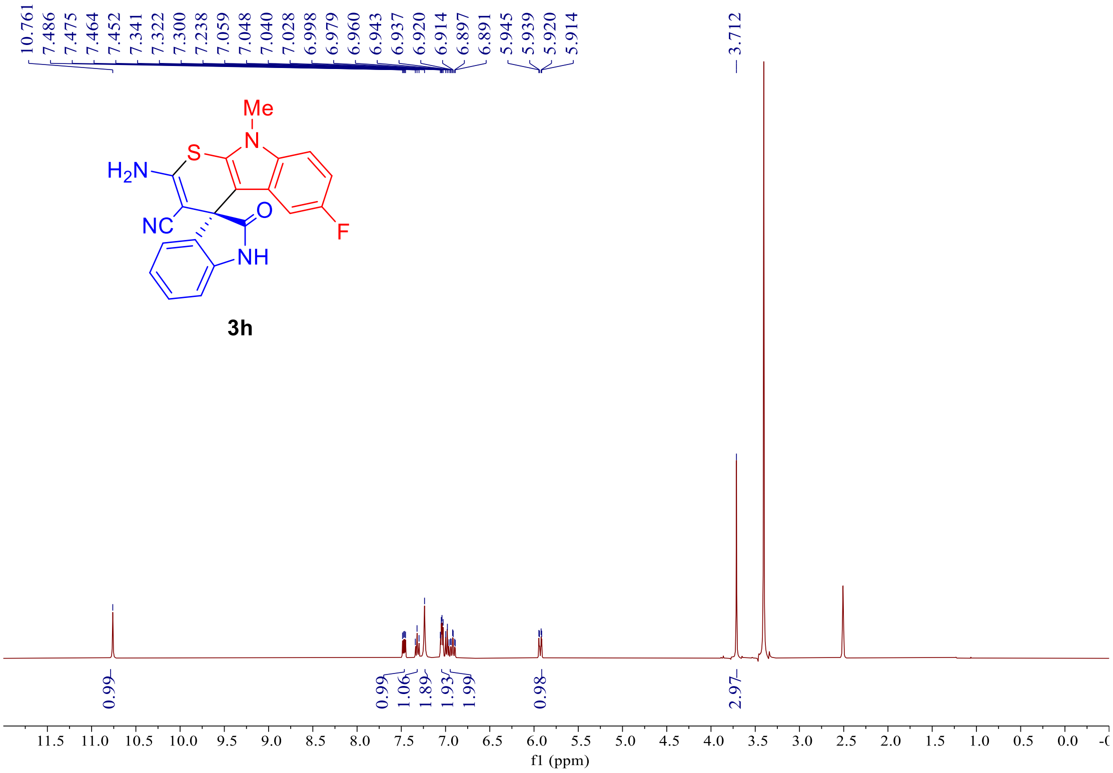

^{13}C NMR (101 MHz, DMSO-d$_6$)

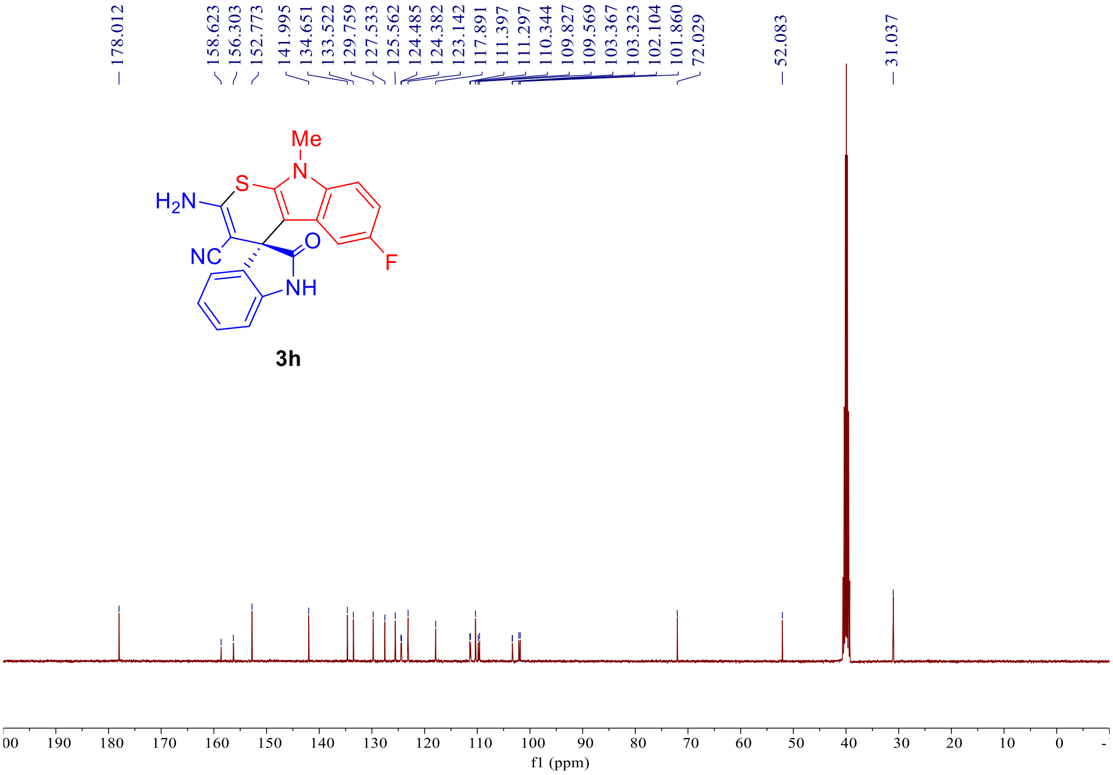

^{19}F NMR (376 MHz, DMSO-d$_6$)

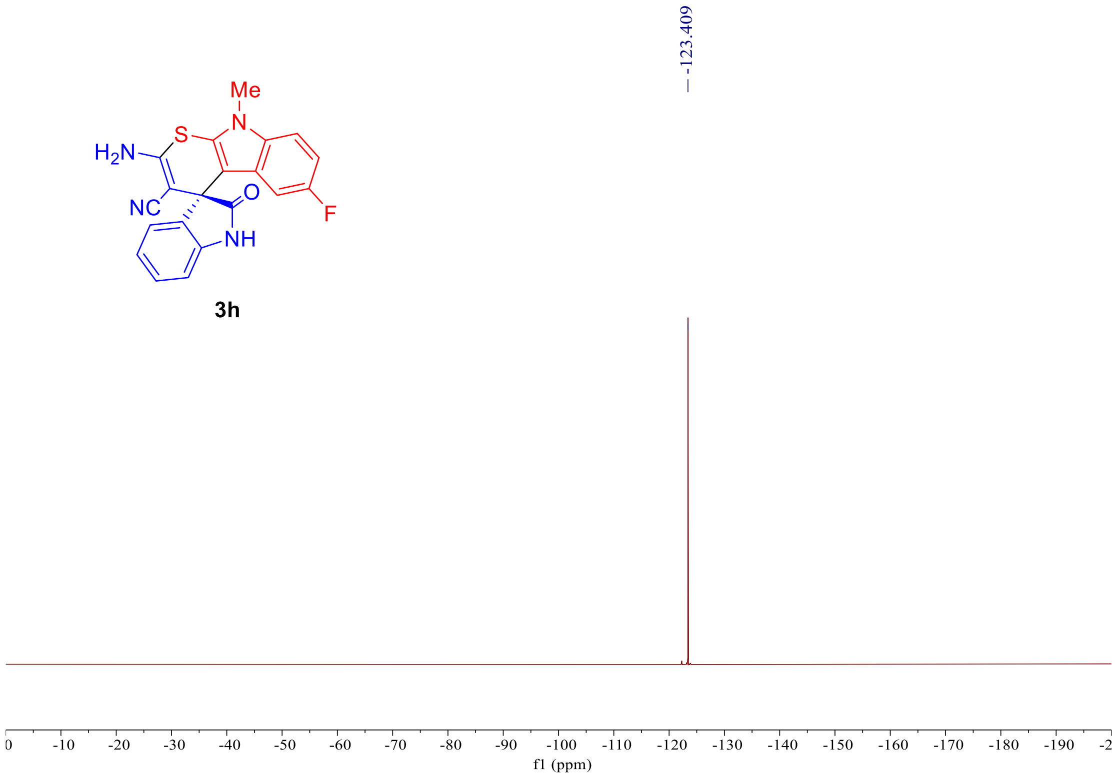

^{1}H NMR (400 MHz, DMSO-d$_6$)

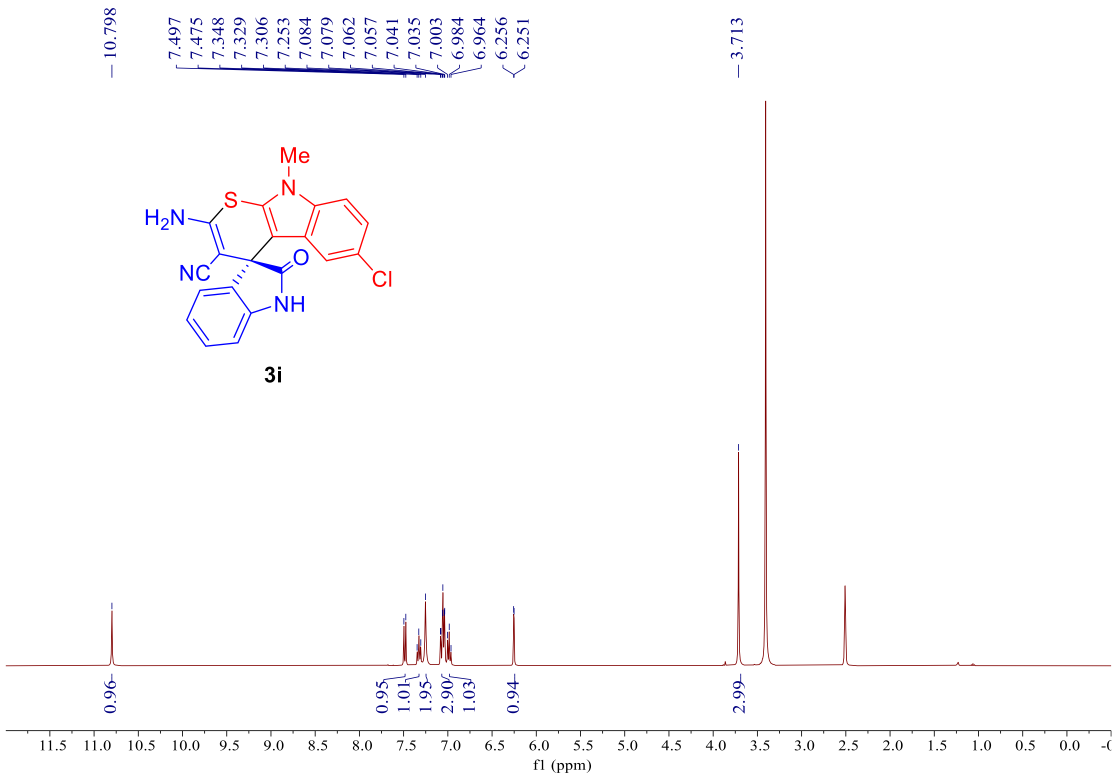

^{13}C NMR (101 MHz, DMSO-d$_6$)

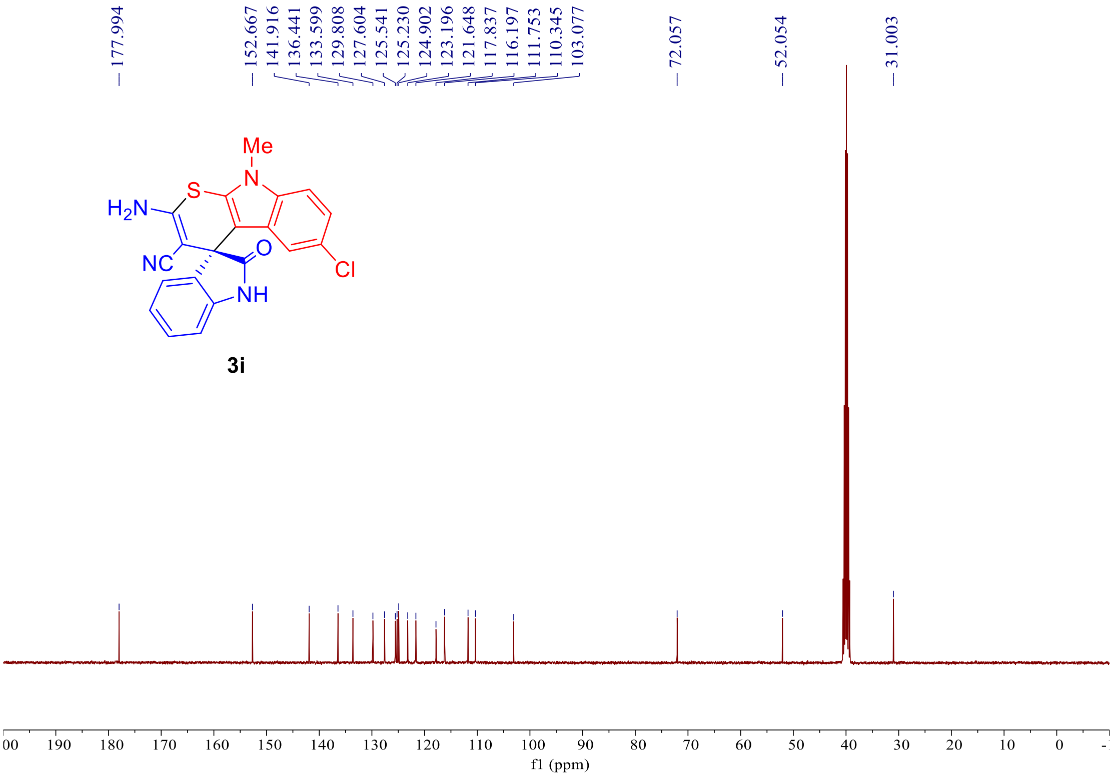

3i

^{1}H NMR (400 MHz, DMSO-d$_6$)

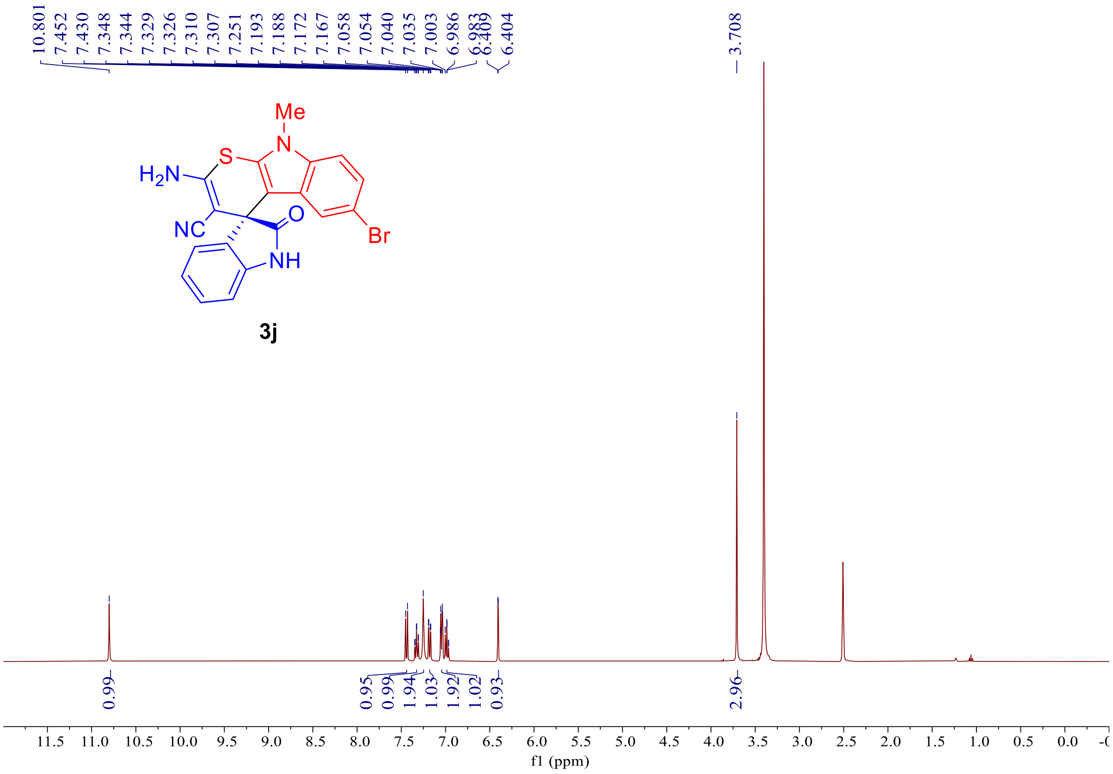

3j

^{13}C NMR (101 MHz, DMSO-d$_6$)

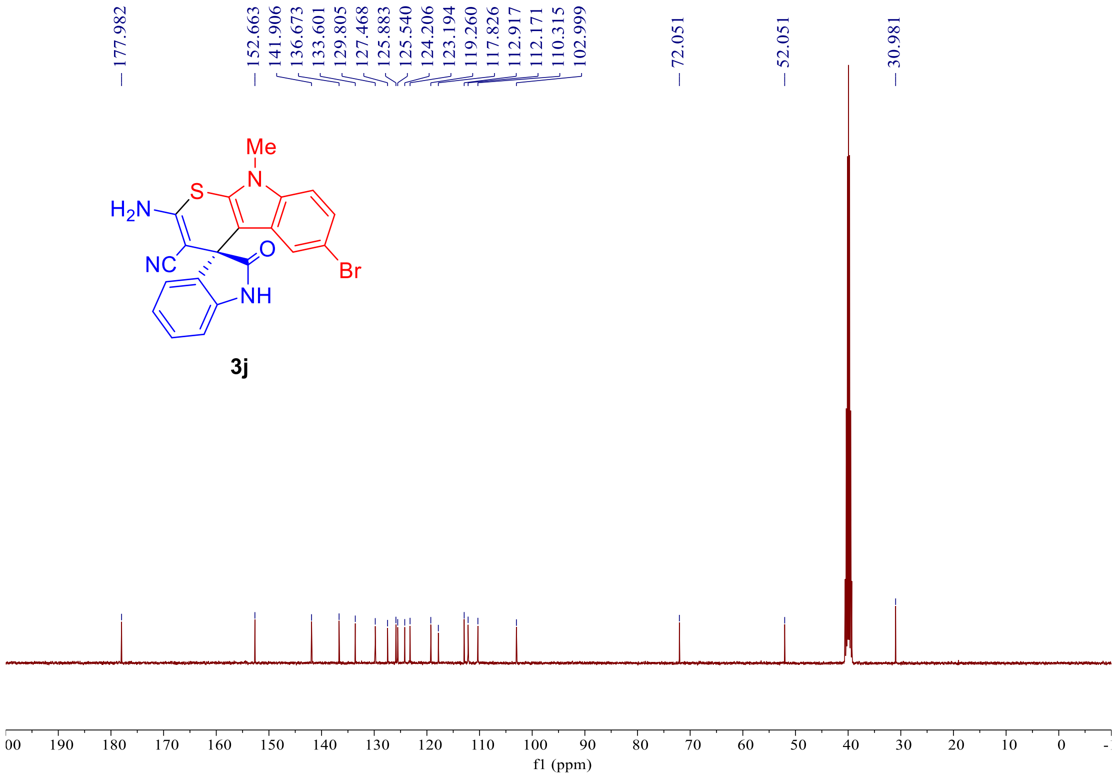

^{1}H NMR (400 MHz, DMSO-d$_6$)

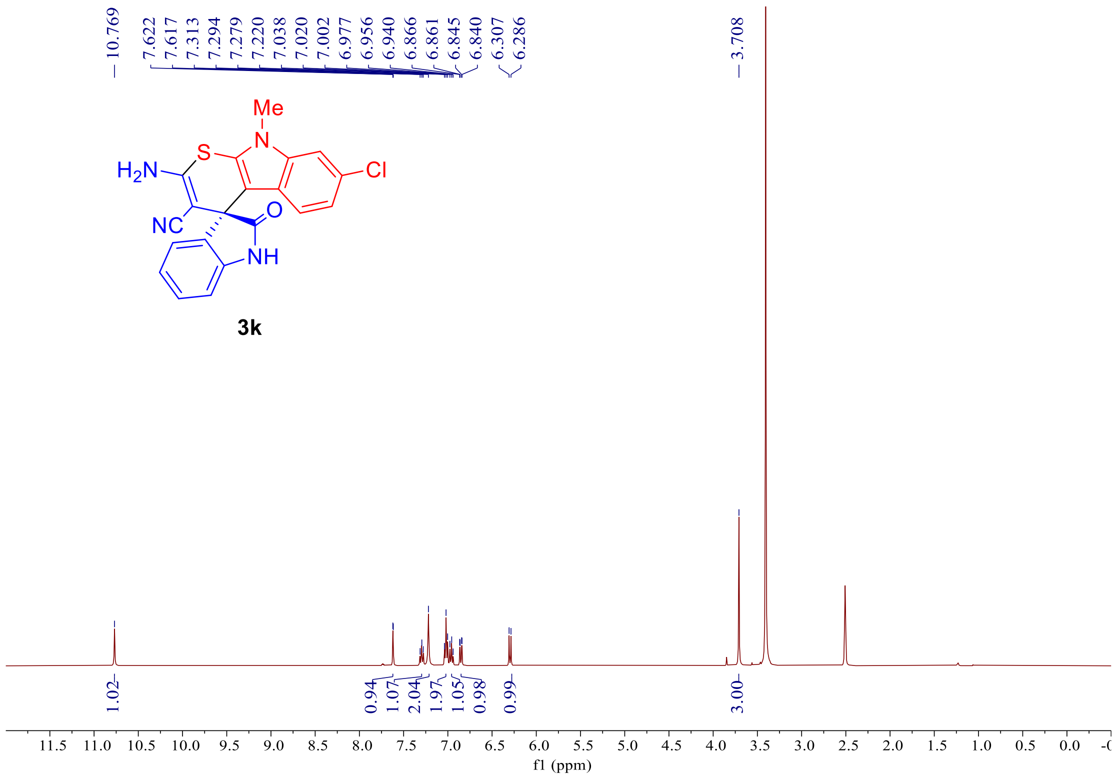

^{13}C NMR (101 MHz, DMSO-d$_6$)

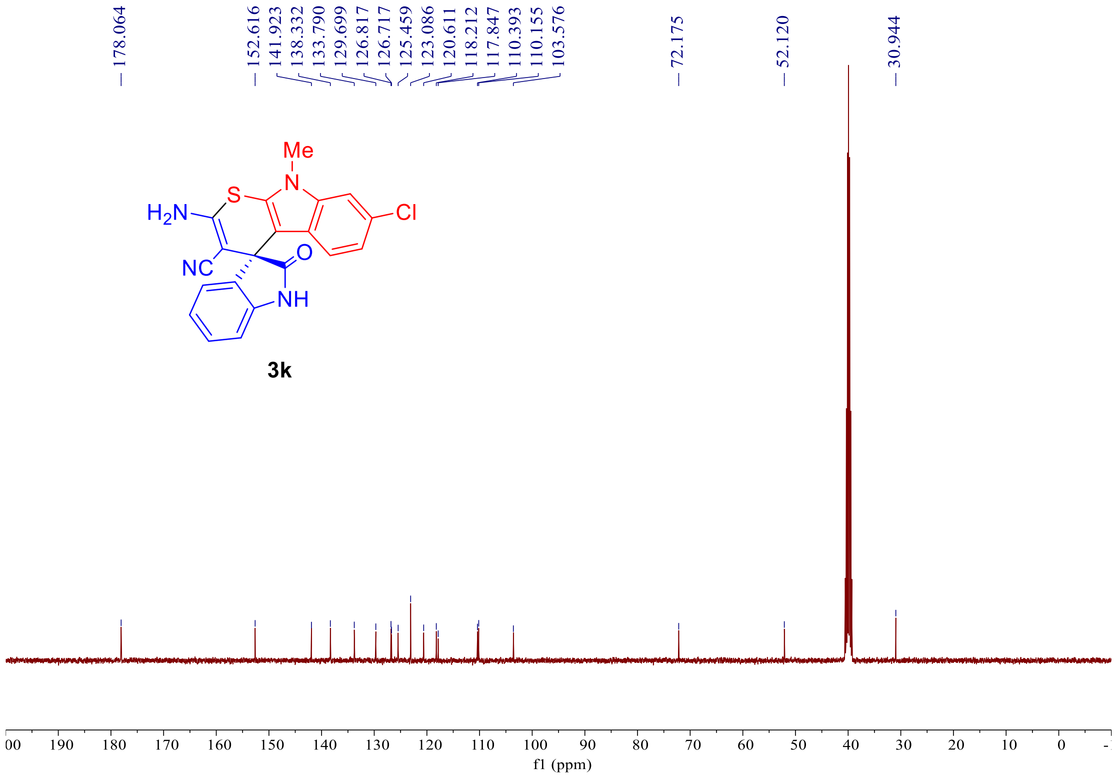

^{1}H NMR (400 MHz, DMSO-d$_6$)

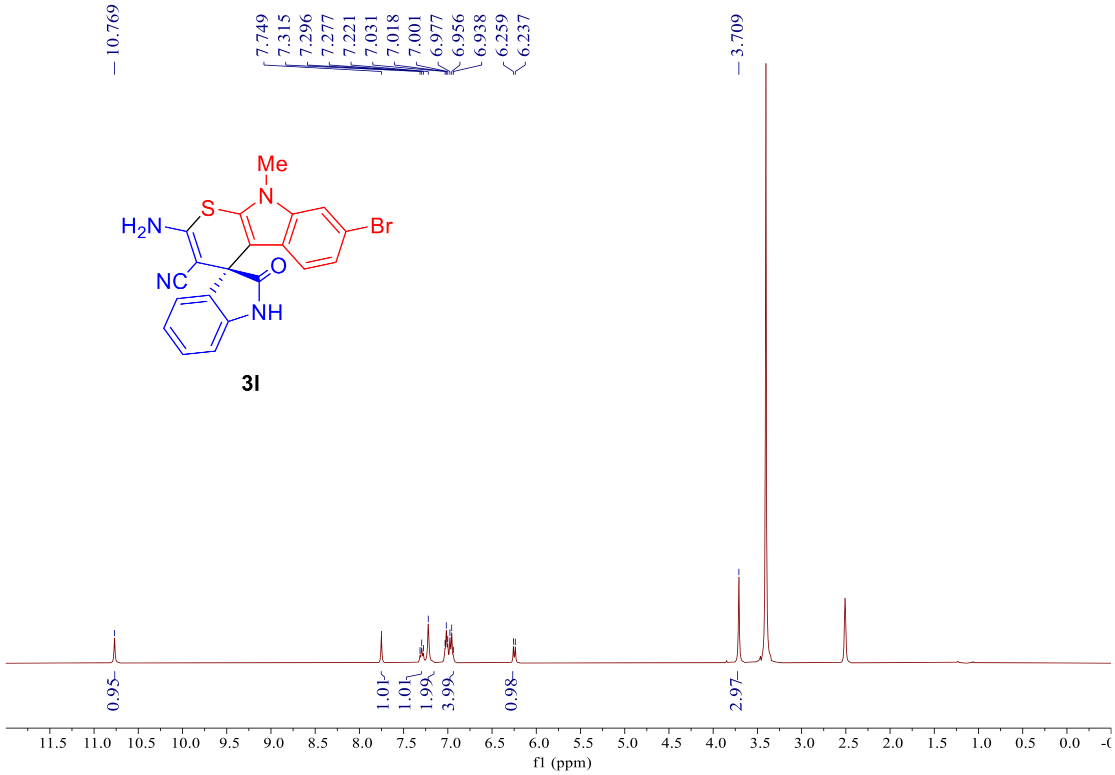

^{13}C NMR (101 MHz, DMSO-d$_6$)

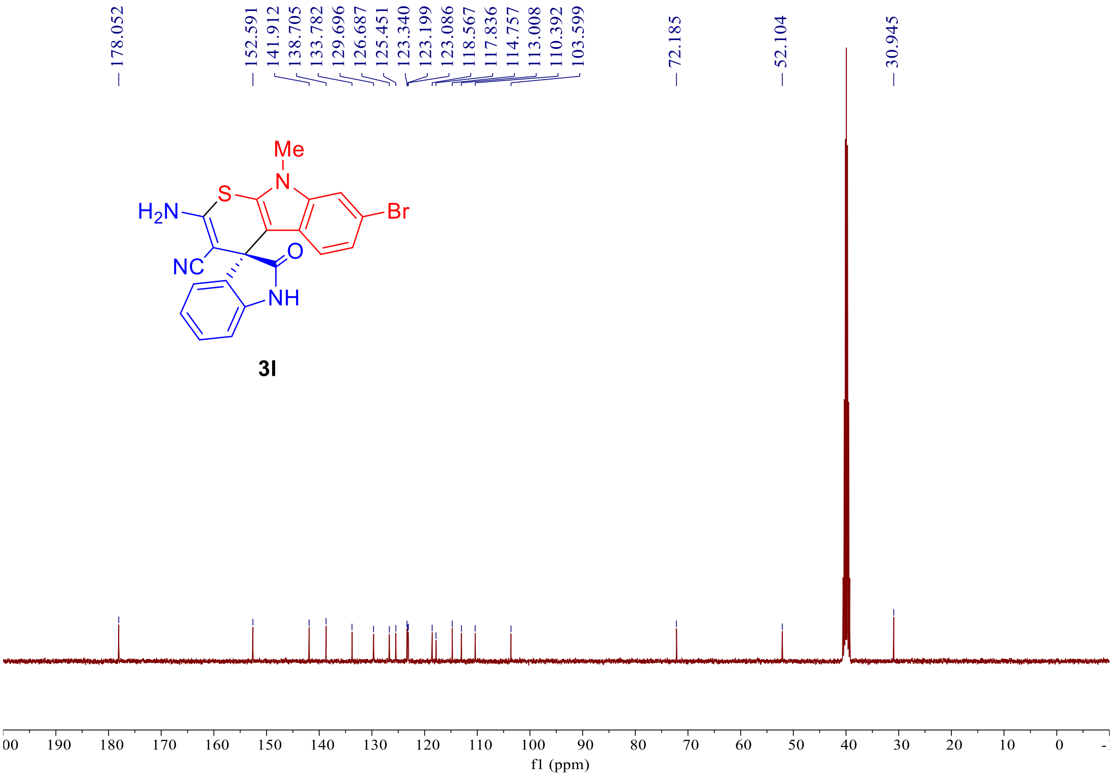

^{1}H NMR (400 MHz, DMSO-d$_6$)

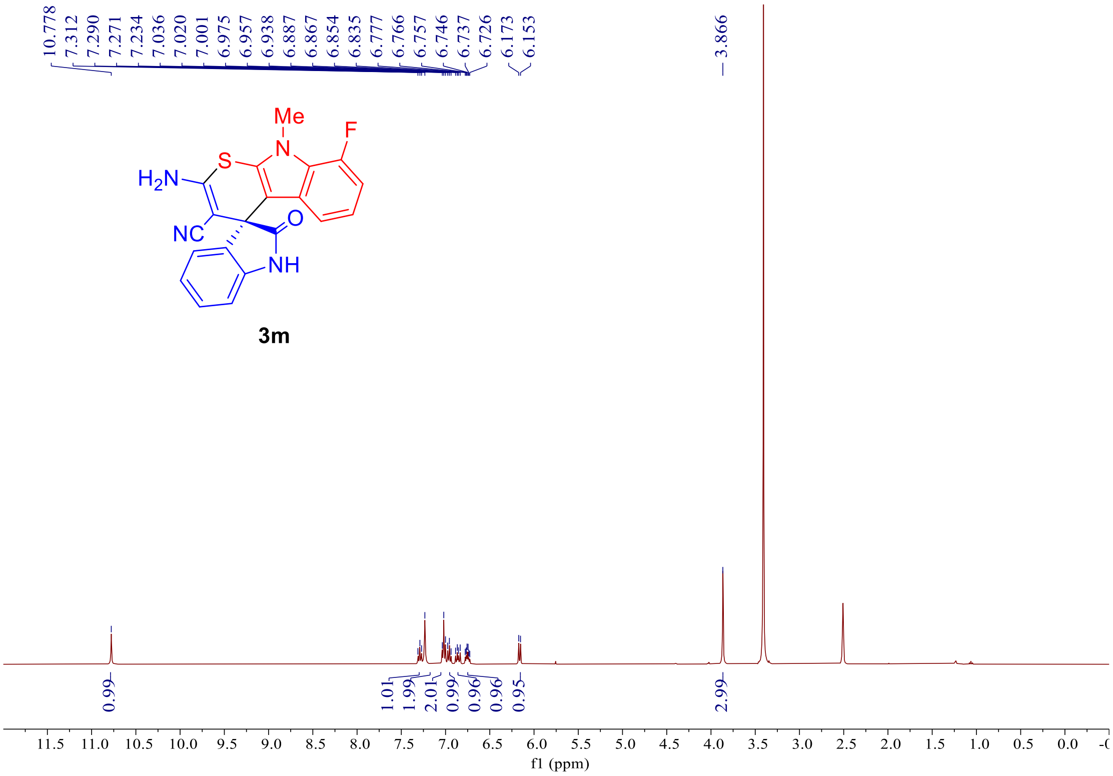

^{13}**C NMR** (101 MHz, DMSO-d$_6$)

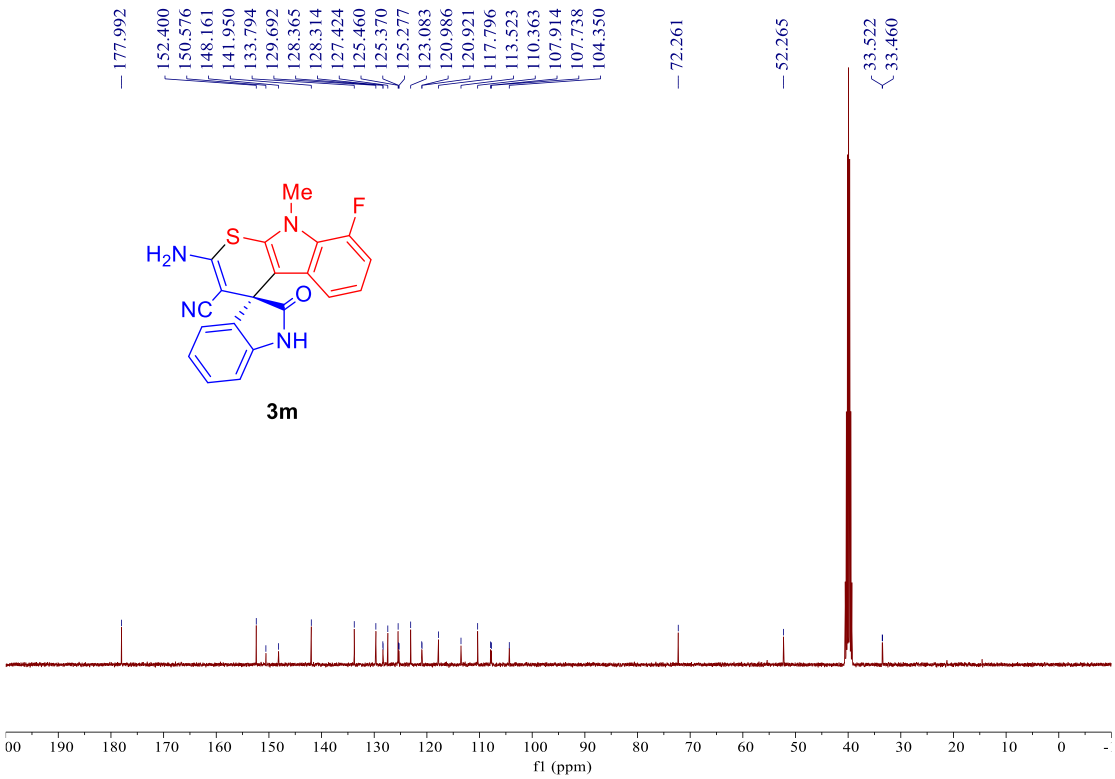

^{19}**F NMR** (376 MHz, DMSO-d$_6$)

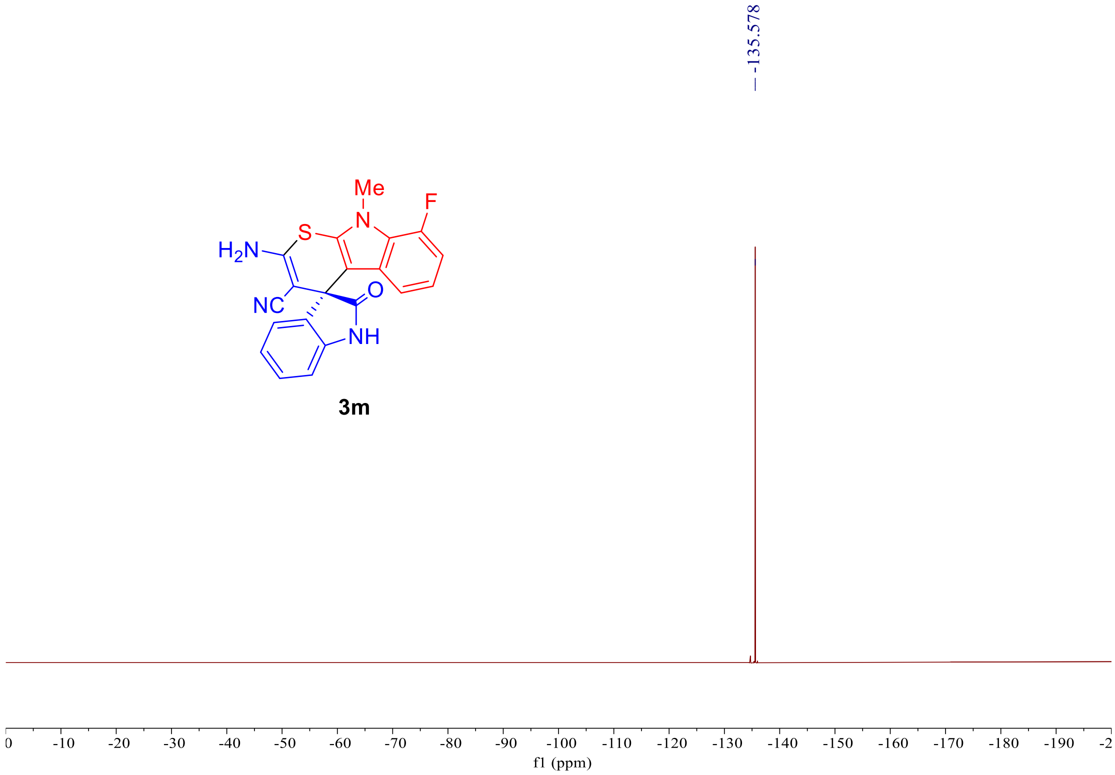

^{1}H NMR (400 MHz, DMSO-d$_6$)

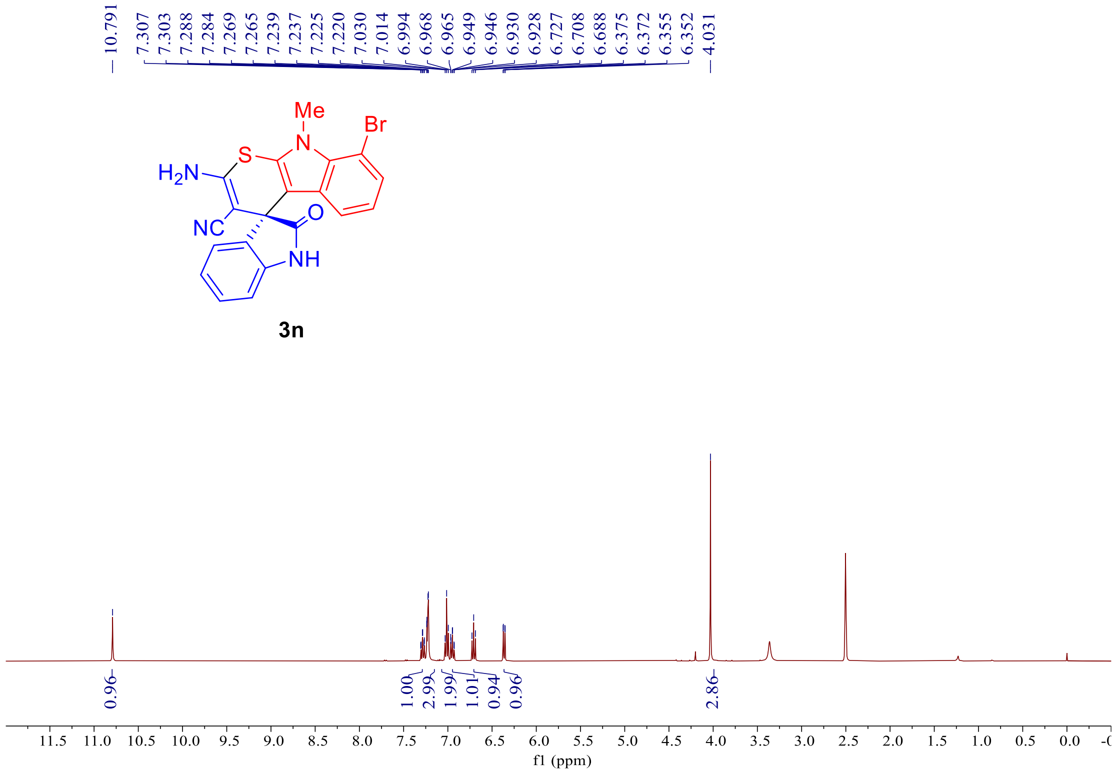

^{13}C NMR (101 MHz, DMSO-d$_6$)

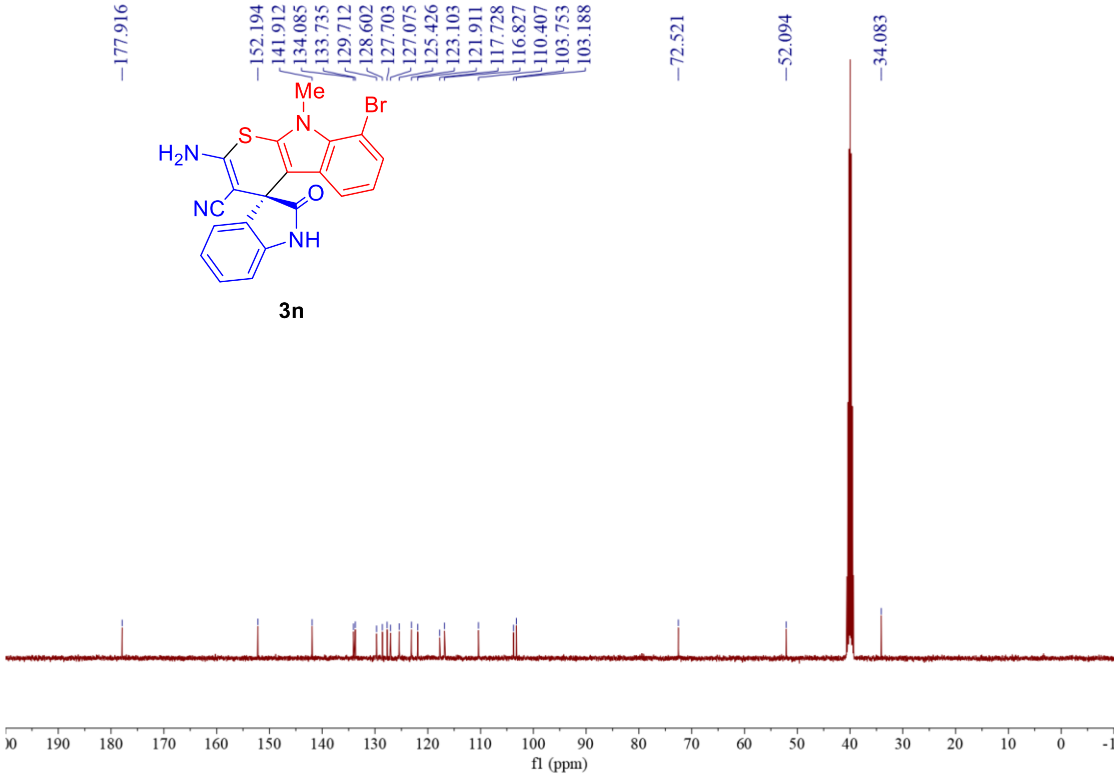

^{1}H NMR (400 MHz, DMSO-d$_6$)

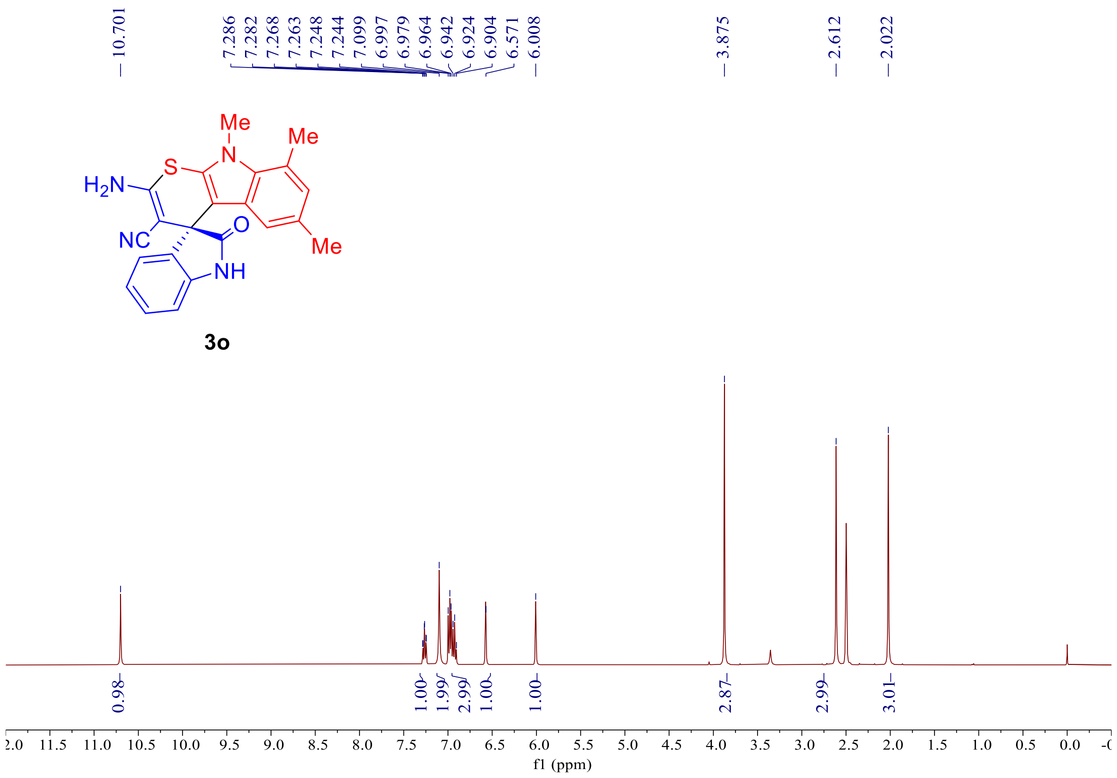

^{13}C NMR (101 MHz, DMSO-d$_6$)

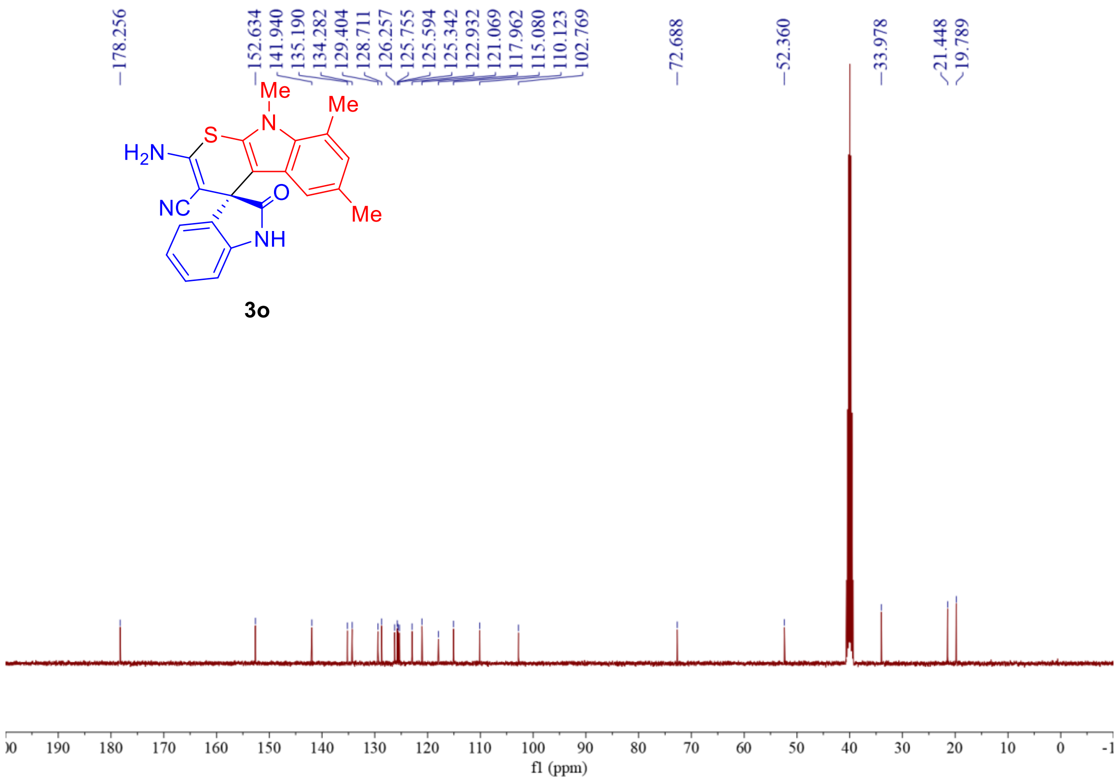

^{1}H NMR (400 MHz, DMSO-d$_6$)

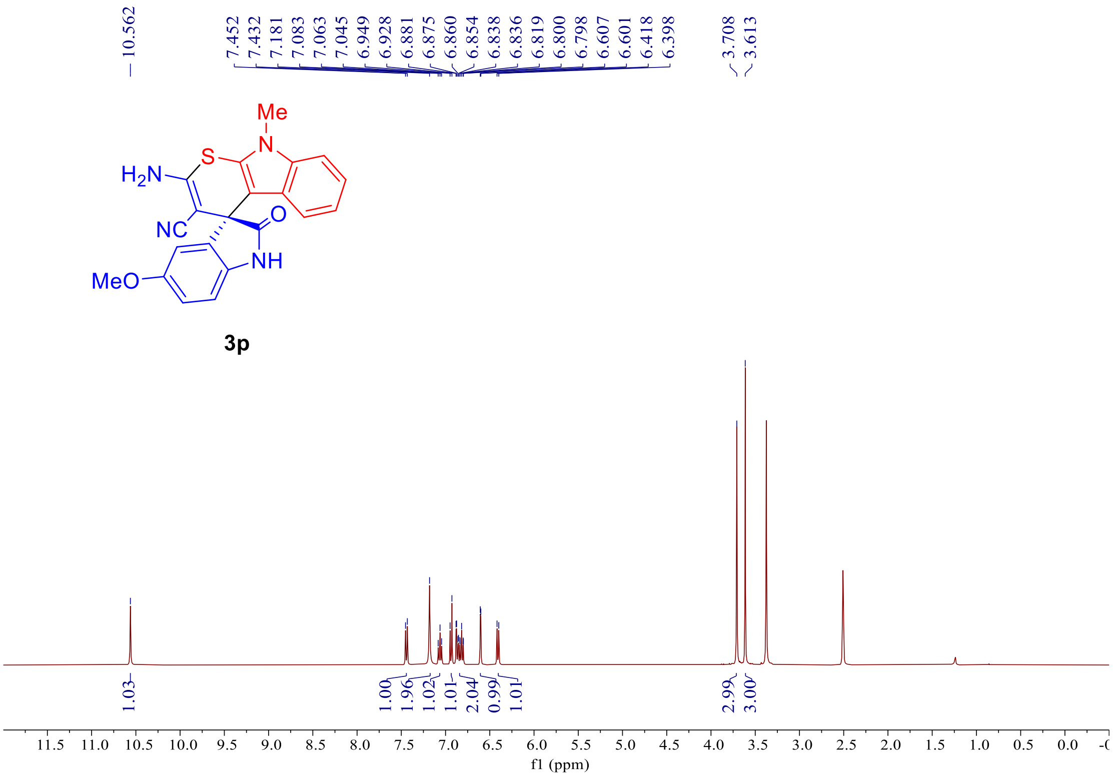

^{13}C NMR (101 MHz, DMSO-d$_6$)

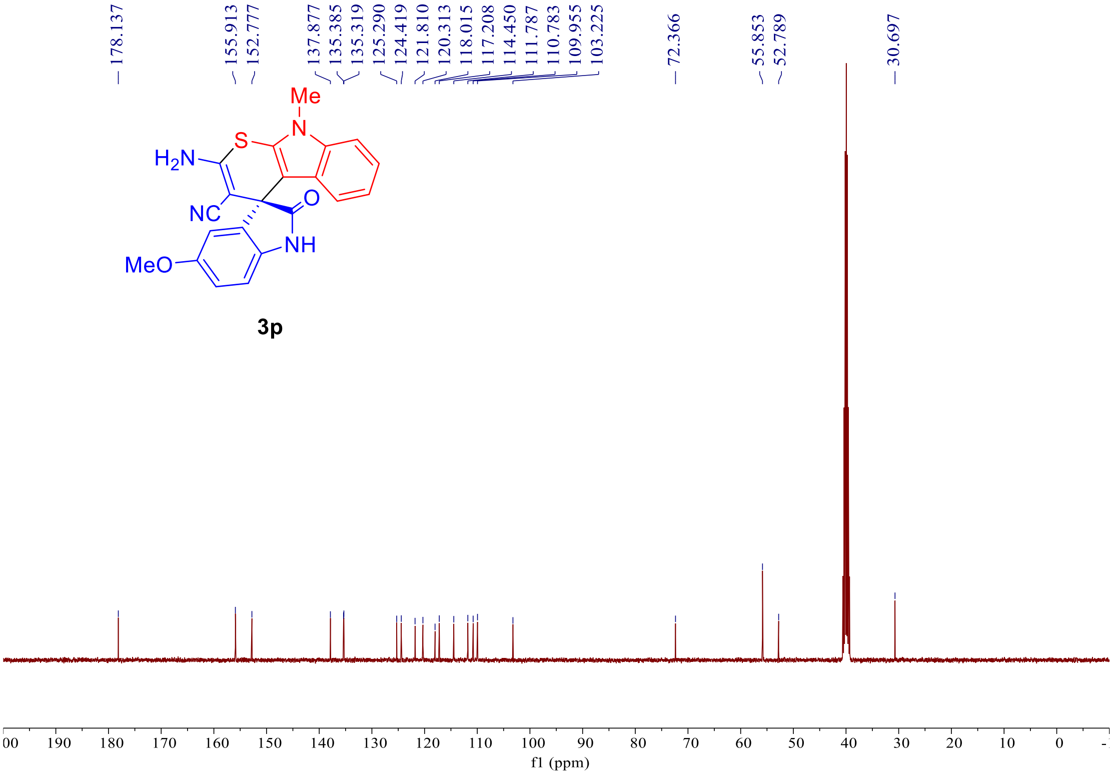

^{1}H NMR (400 MHz, DMSO-d$_6$)

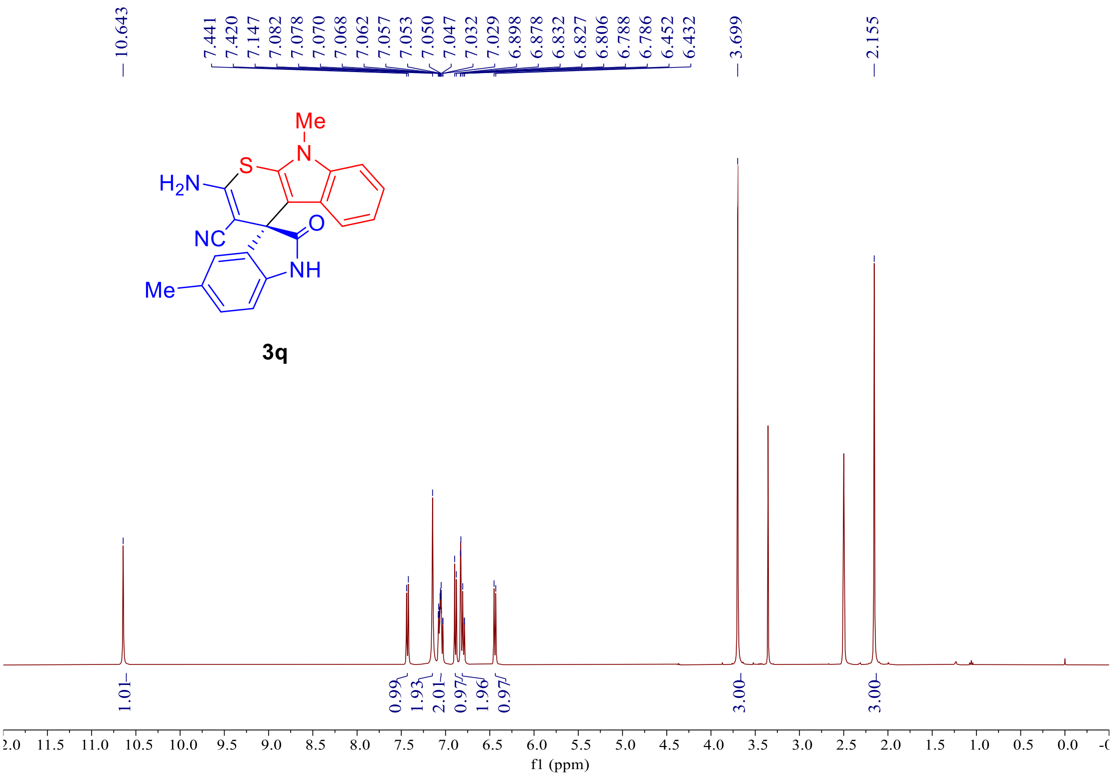

^{13}C NMR (101 MHz, DMSO-d$_6$)

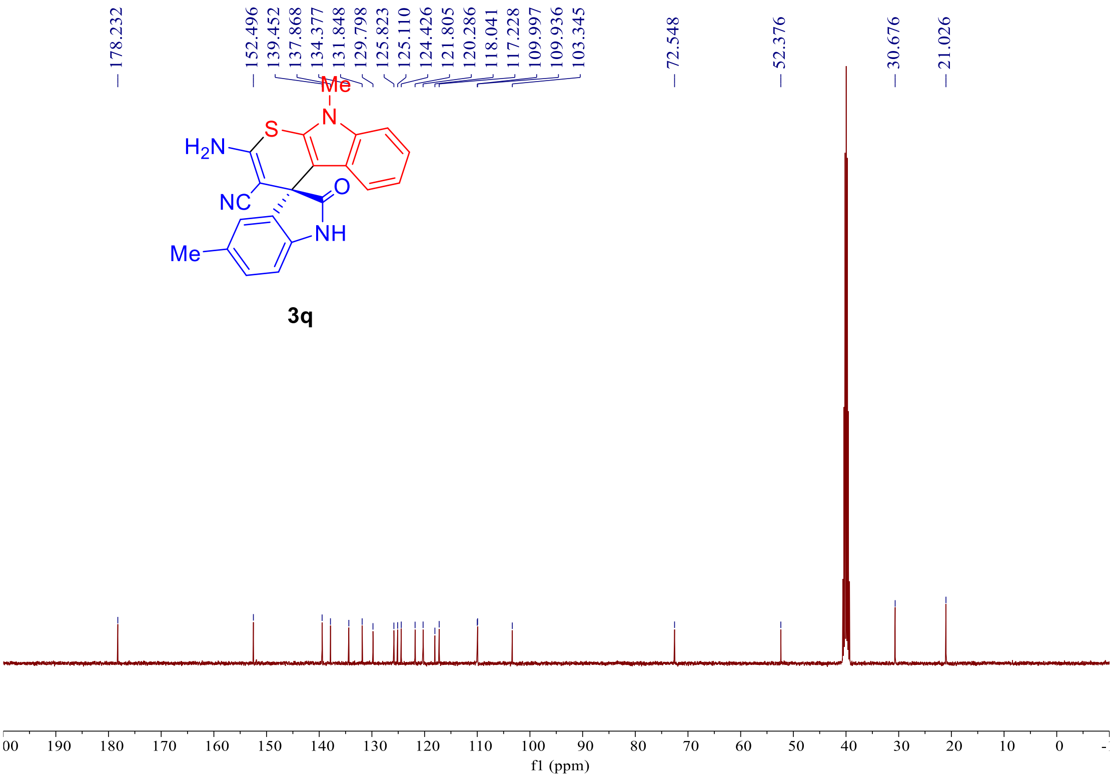

^{1}H NMR (400 MHz, DMSO-d$_6$)

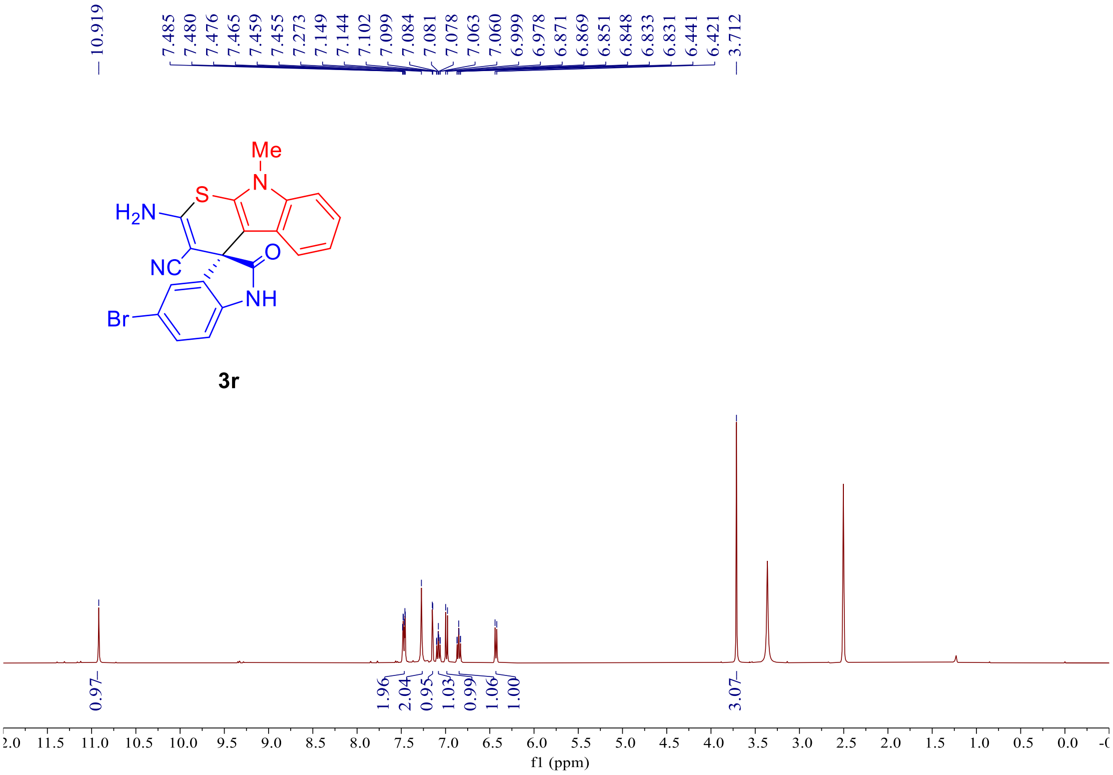

^{13}C NMR (101 MHz, DMSO-d$_6$)

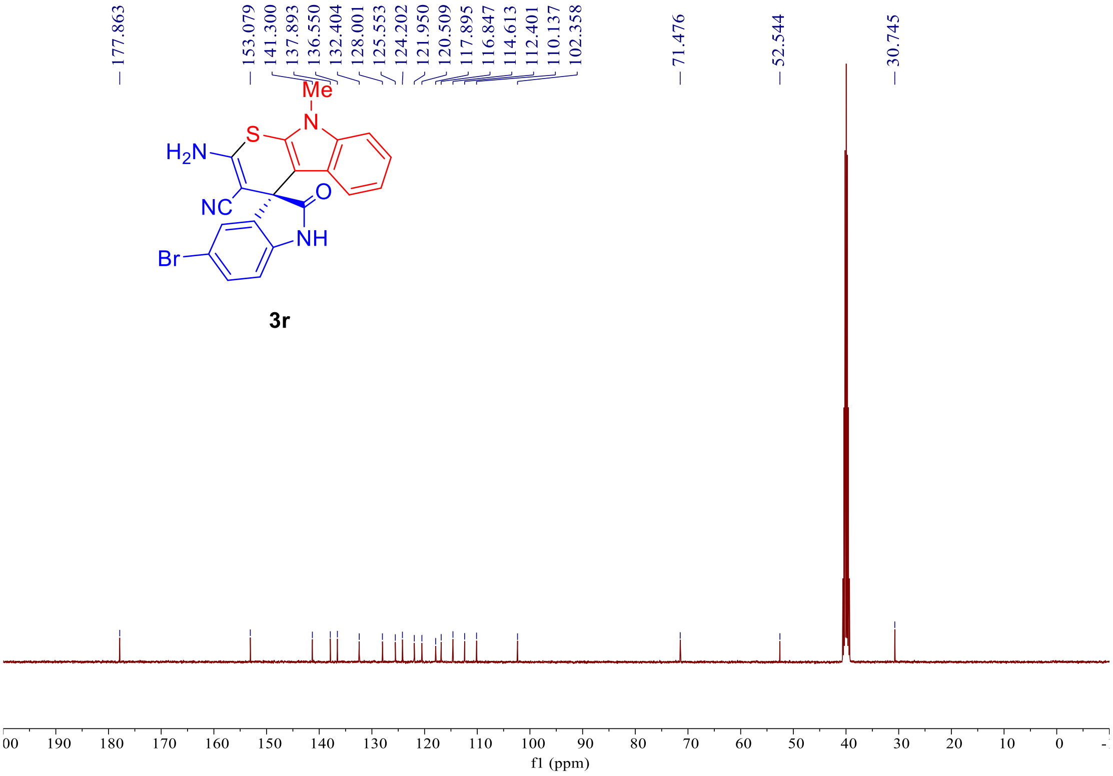

^{1}H NMR (400 MHz, DMSO-d$_6$)

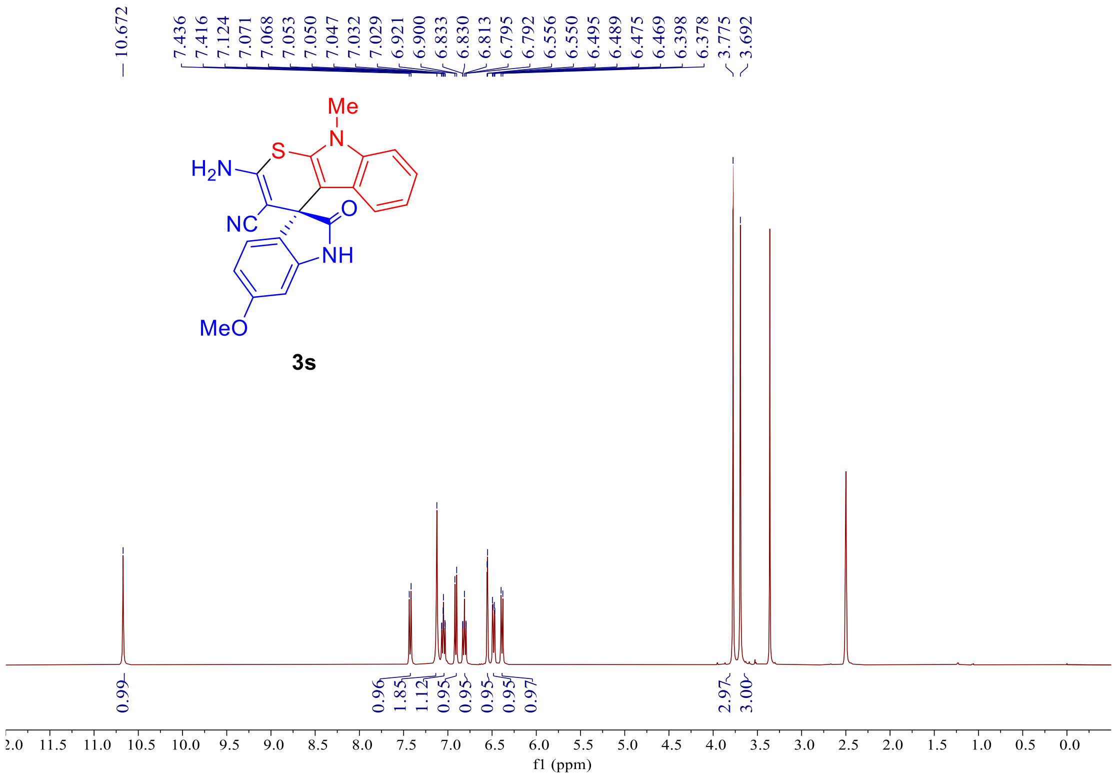

^{13}C NMR (101 MHz, DMSO-d$_6$)

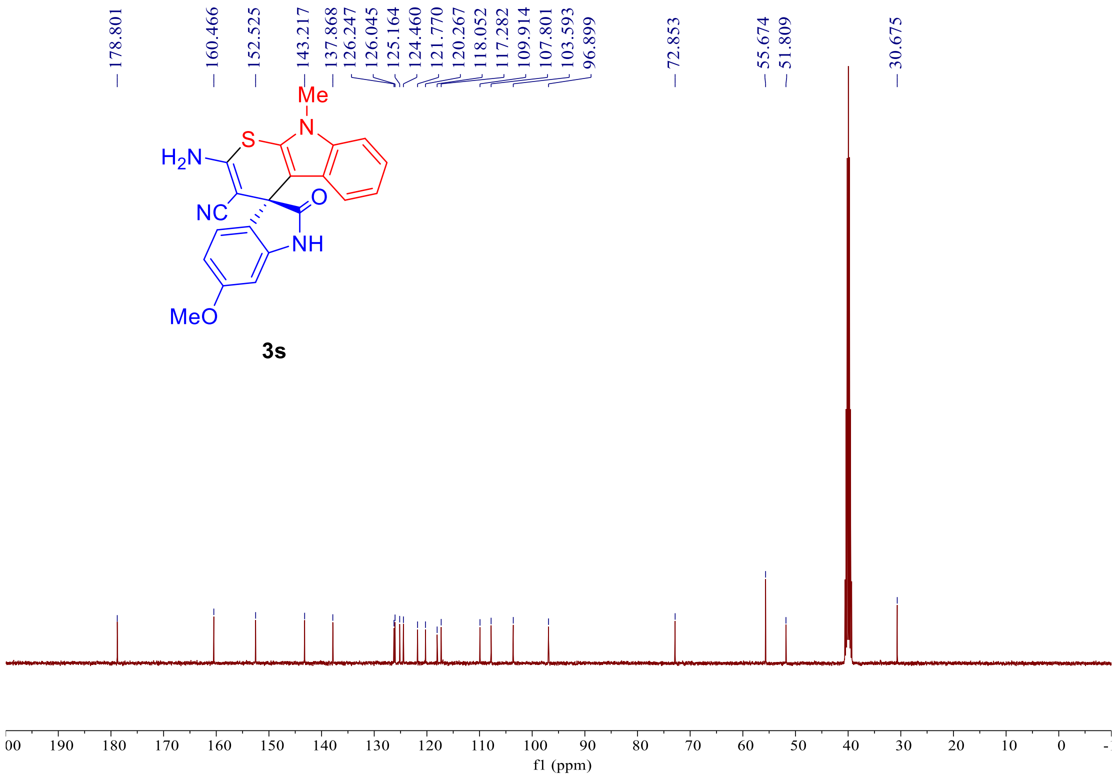

^{1}H NMR (400 MHz, DMSO-d$_6$)

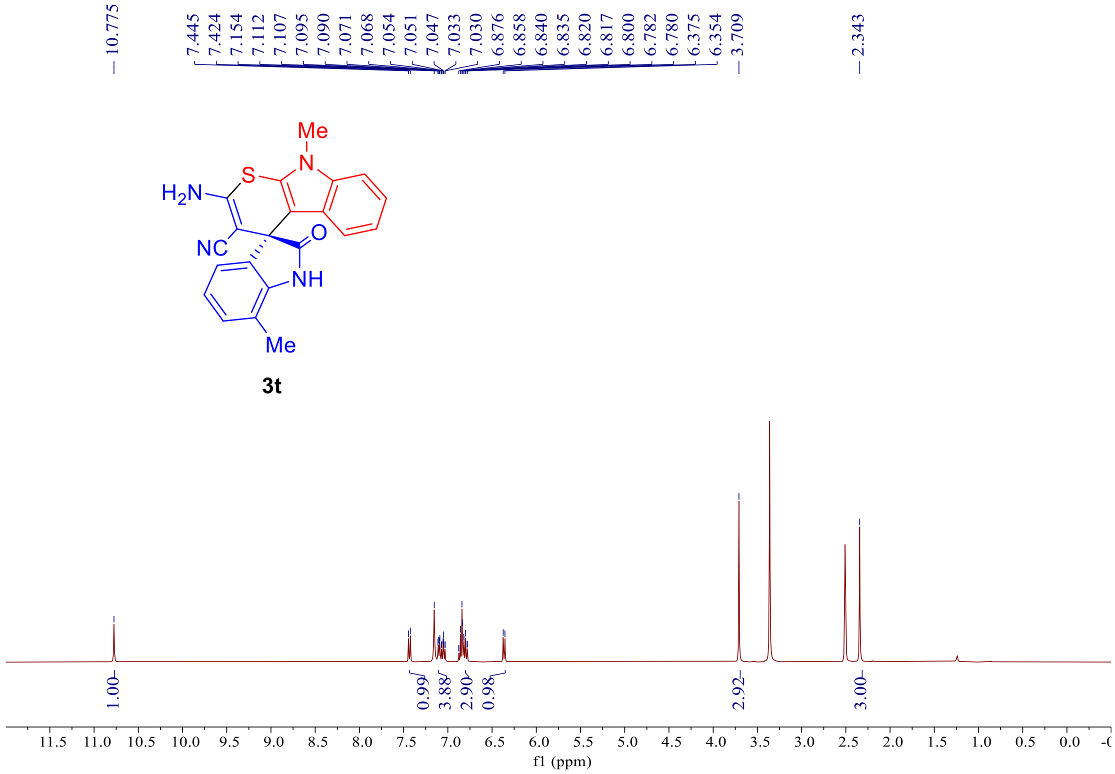

^{13}C NMR (101 MHz, DMSO-d$_6$)

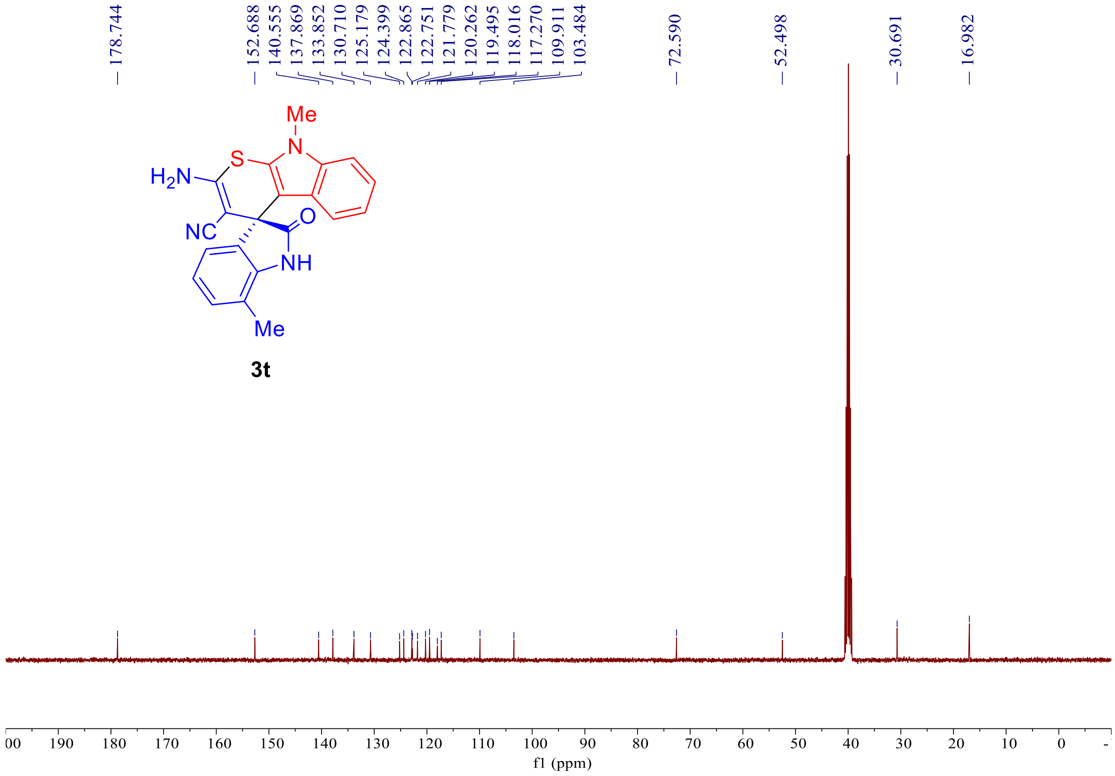

^{1}H NMR (400 MHz, DMSO-d$_6$)

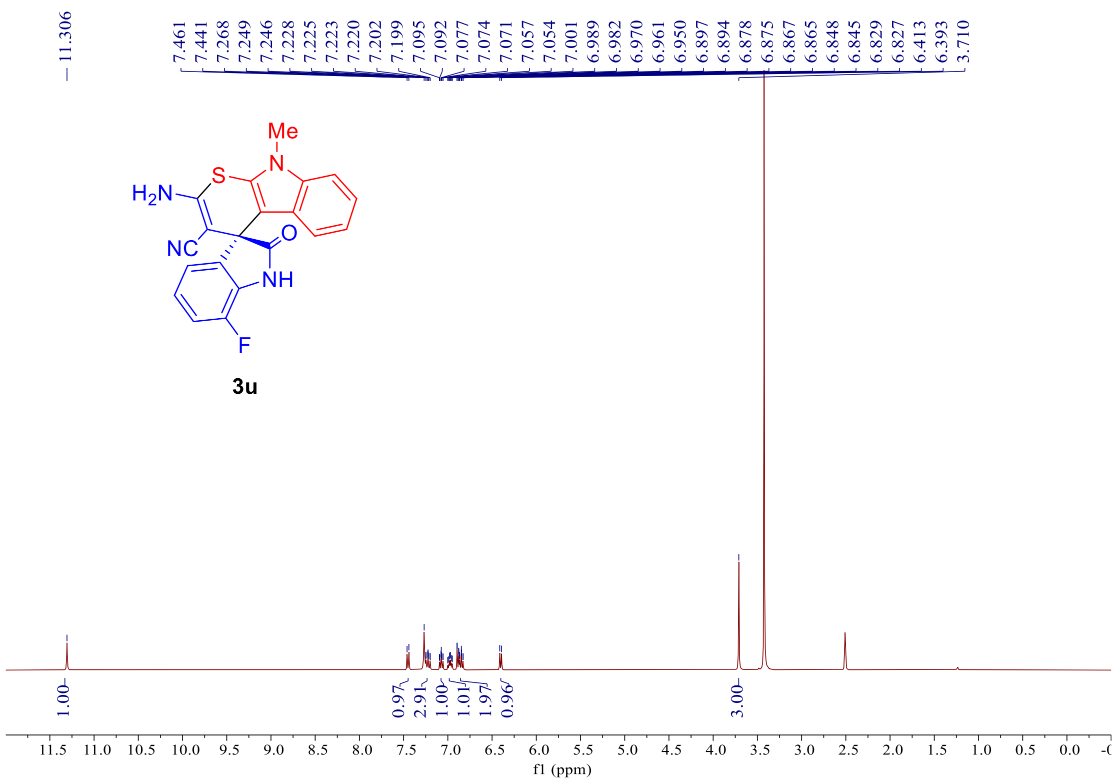

3u

^{13}C NMR (101 MHz, DMSO-d$_6$)

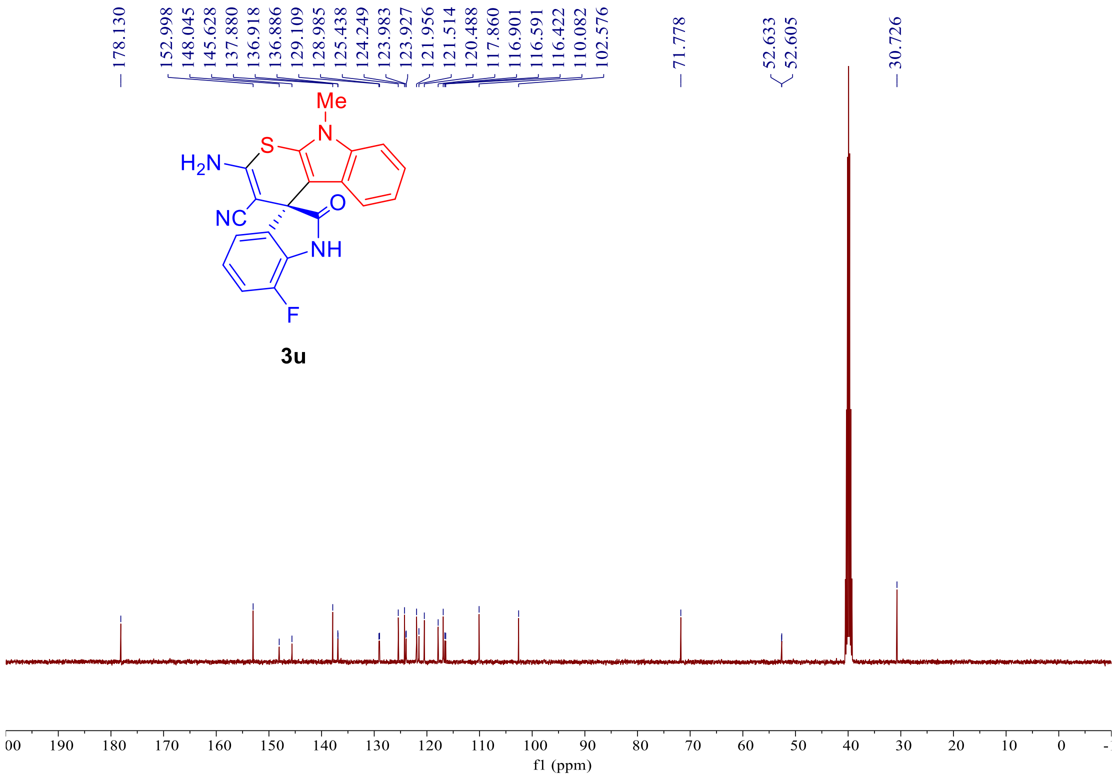

3u

^{19}F NMR (376 MHz, DMSO-d$_6$)

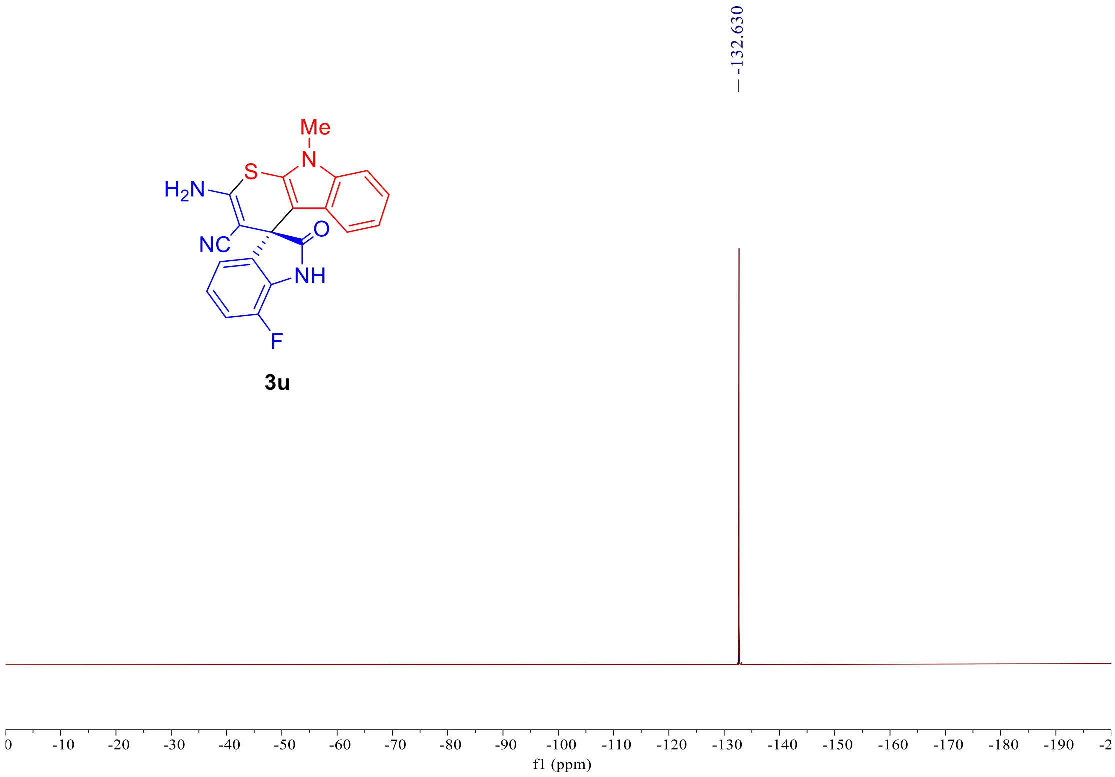

^{1}H NMR (400 MHz, DMSO-d$_6$)

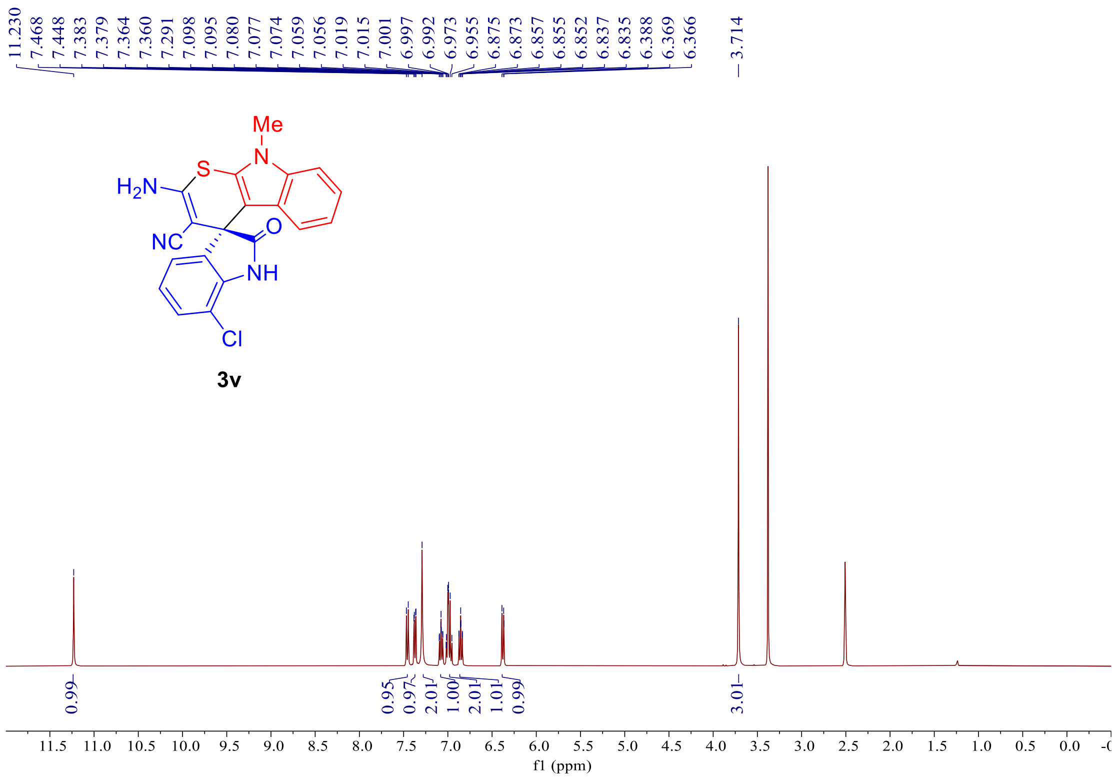

^{13}C NMR (101 MHz, DMSO-d$_6$)

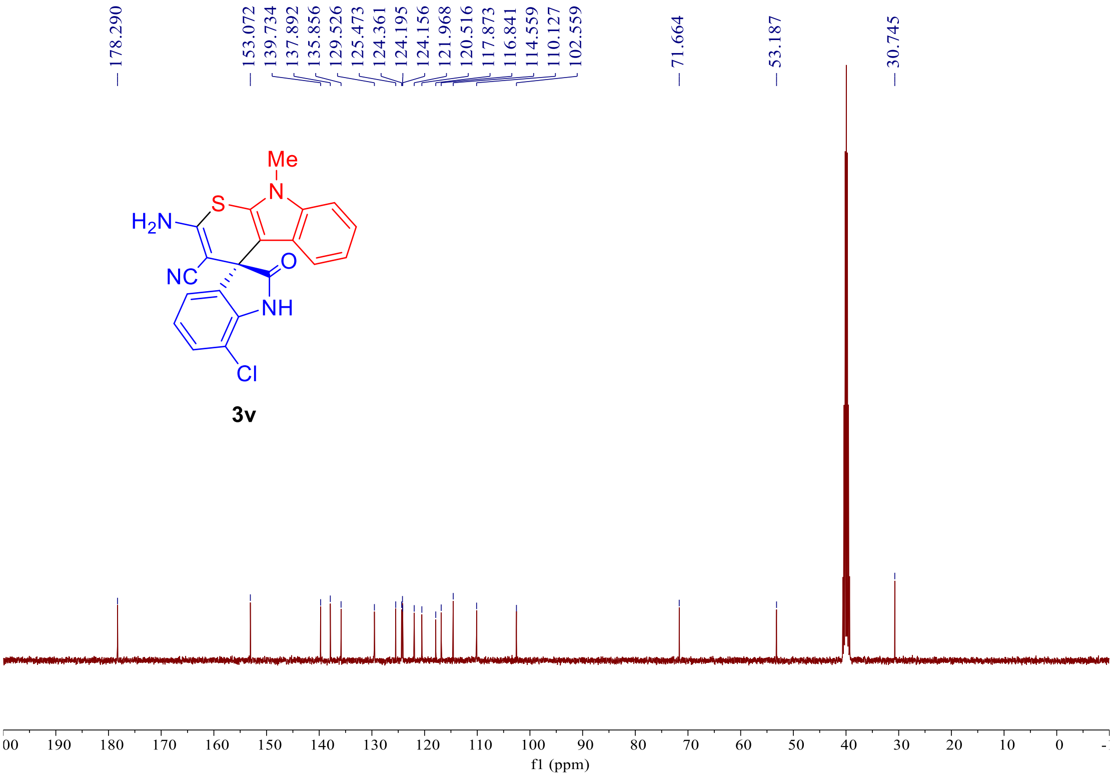

^{1}H NMR (400 MHz, DMSO-d$_6$)

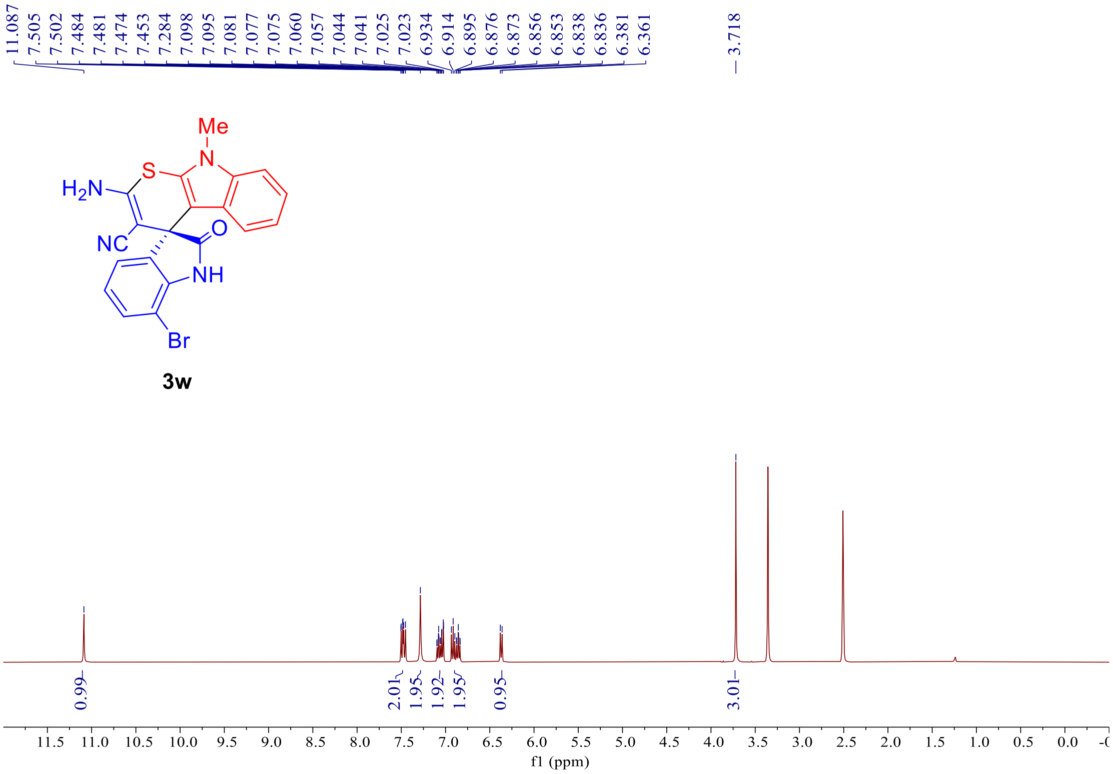

^{13}**C NMR** (101 MHz, DMSO-d_6)

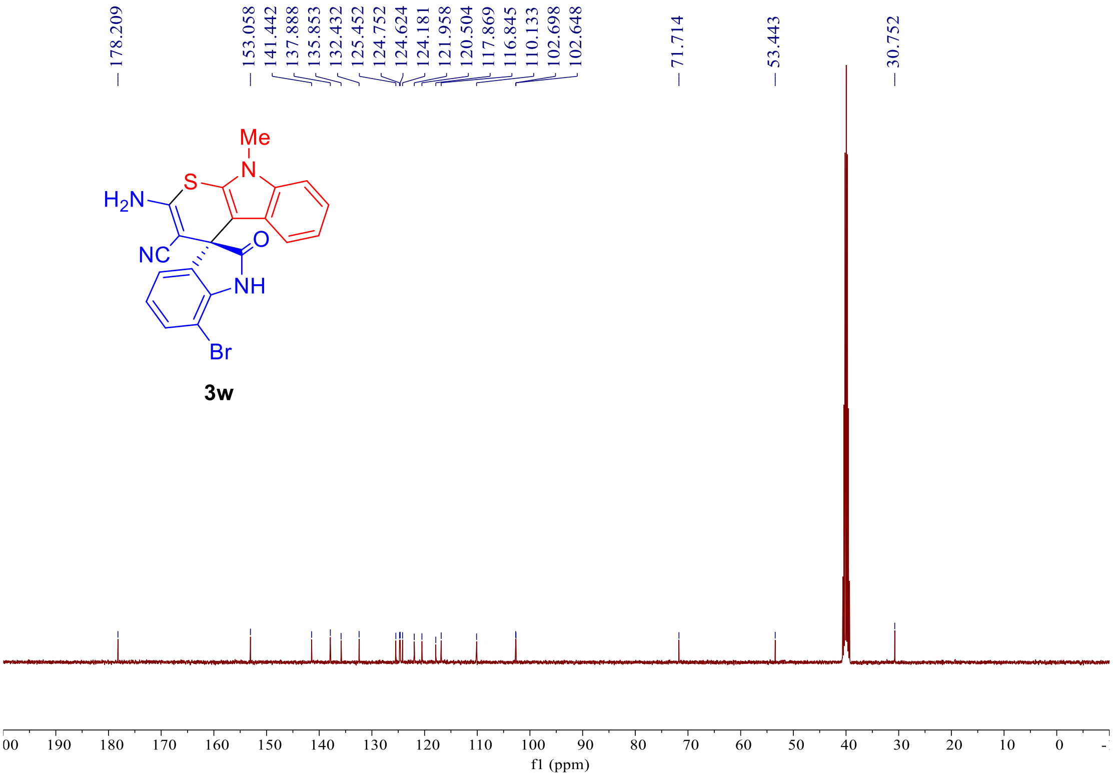

^{1}**H NMR** (400 MHz, DMSO-d_6)

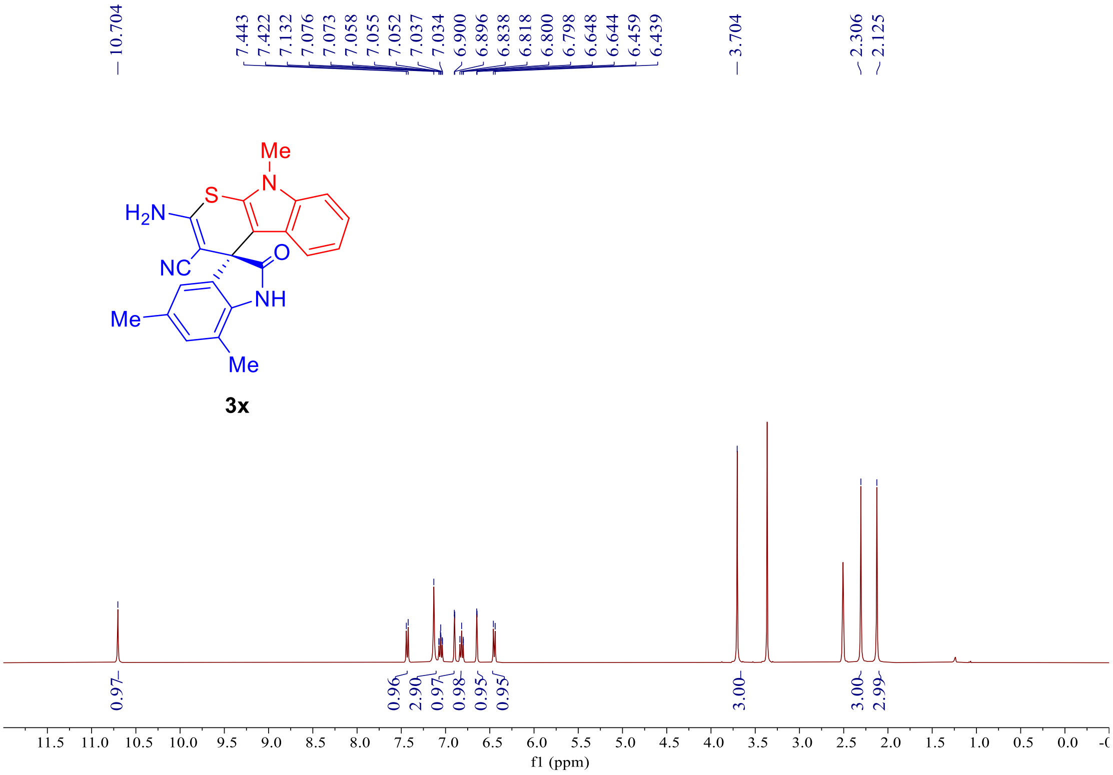

^{13}C NMR (101 MHz, DMSO-d$_6$)

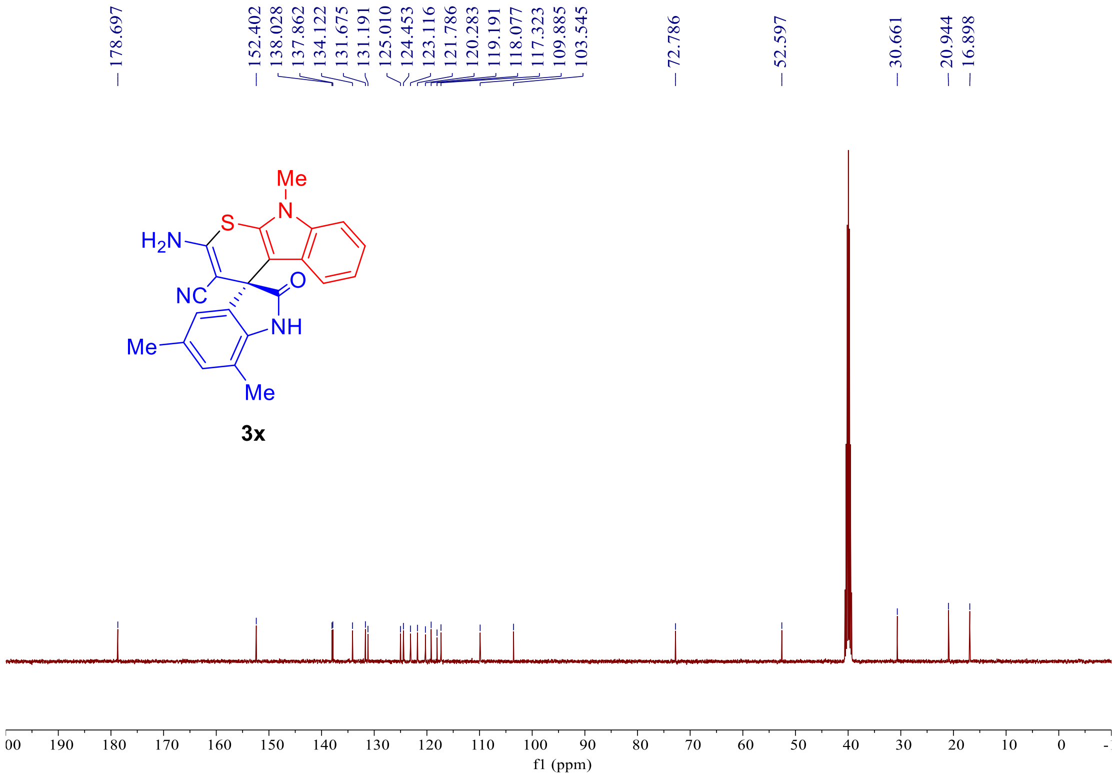

^{1}H NMR (400 MHz, DMSO-d$_6$)

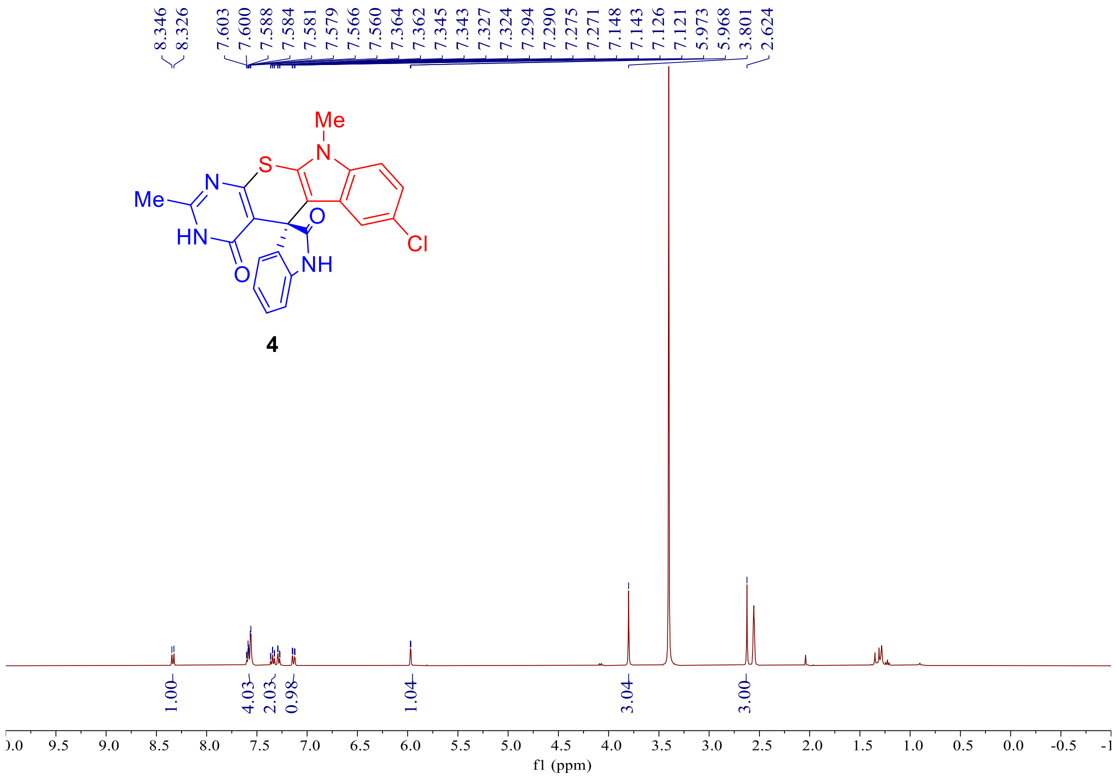

^{13}C NMR (101 MHz, DMSO-d_6)

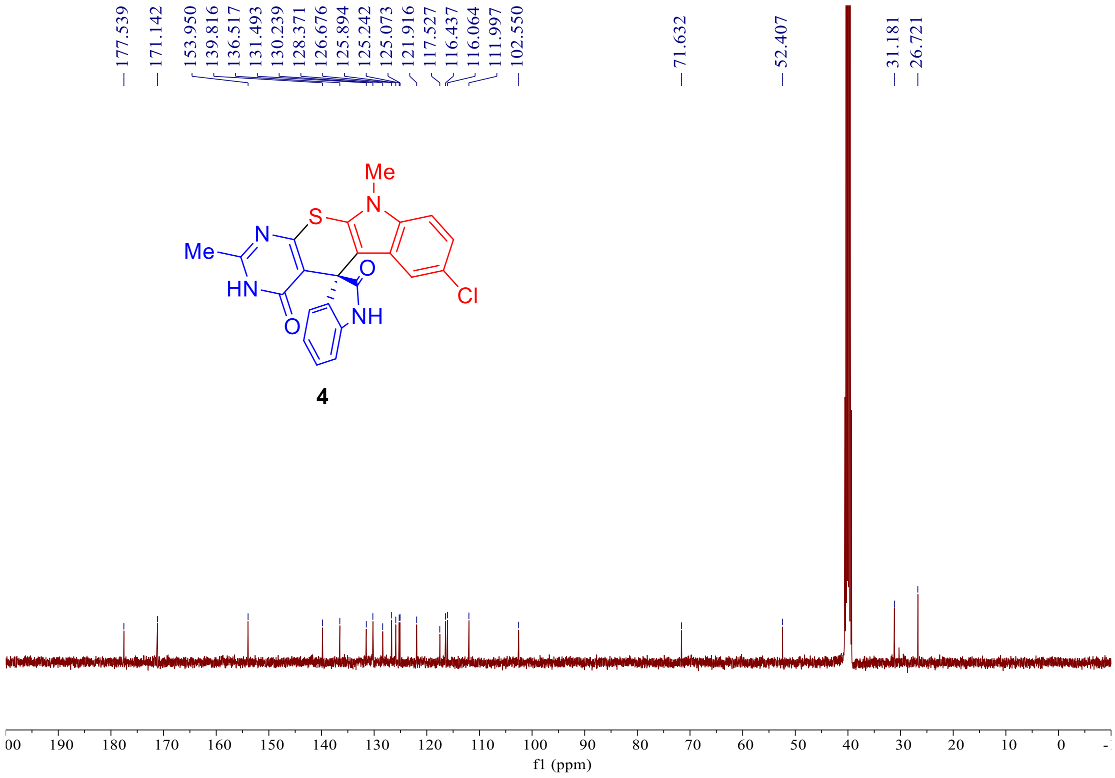

HPLC spectra of compounds

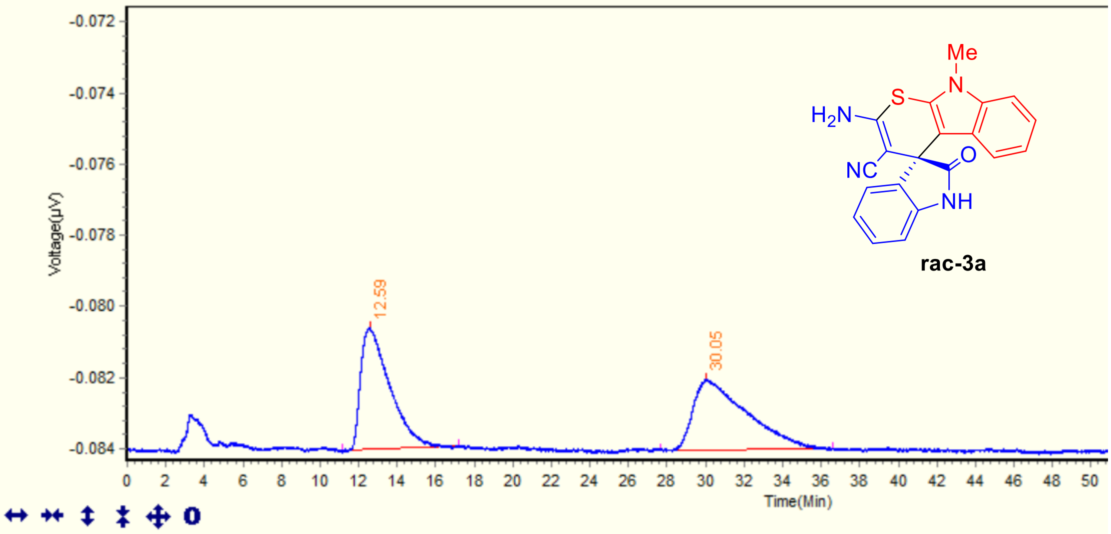

Integration Result | Calculation Result | TimeTable

No.	Retention Time	Peak Area	Peak Height	Peak Area(%)	Peak Width	
1	12.59	178500	1708	49.07%	6.036	BB
2	30.05	185239	978	50.93%	8.939	BB
Total		363,739	2,686	100.00%		

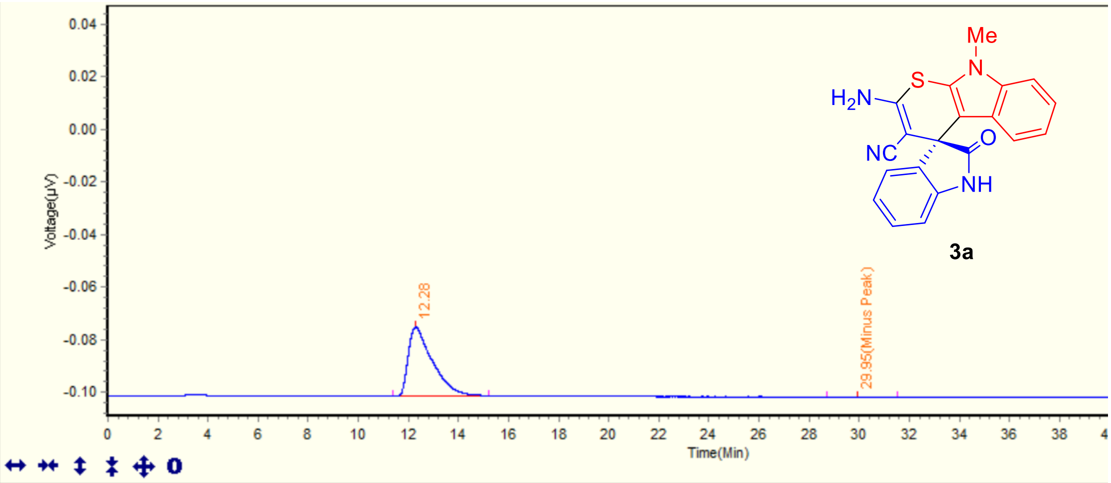

Integration Result | Calculation Result | TimeTable

No.	Retention Time	Peak Area	Peak Height	Peak Area(%)	Peak Width	
1	12.28	875437	13094	99.96%	3.827	BB
2	29.95	328	28	0.04%	2.817	BB
Total		875,765	13,122	100.00%		

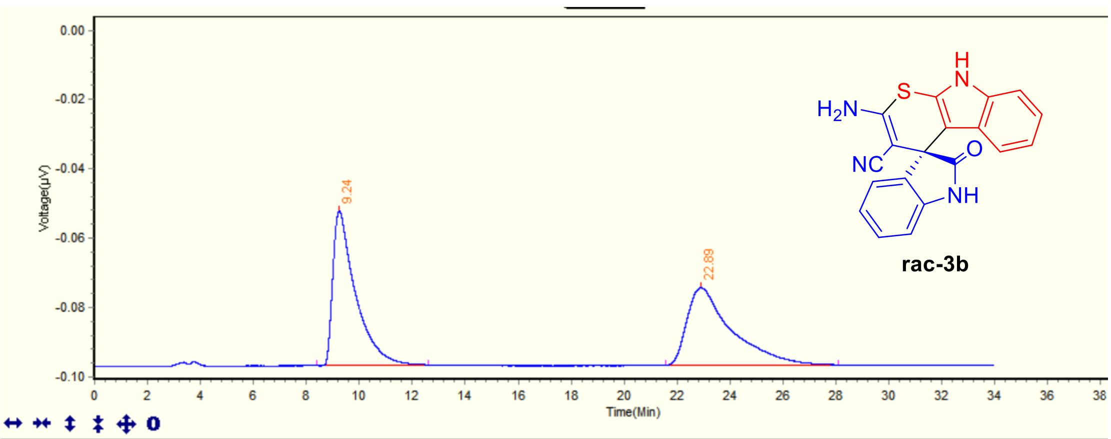

Integration Result | Calculation Result | TimeTable

No.	Retention Time	Peak Area	Peak Height	Peak Area(%)	Peak Width	Peak Type
1	9.24	1324179	22347	50.40%	4.216	BB
2	22.89	1302951	11187	49.60%	6.524	BB
Total		2,627,130	33,534	100.00%		

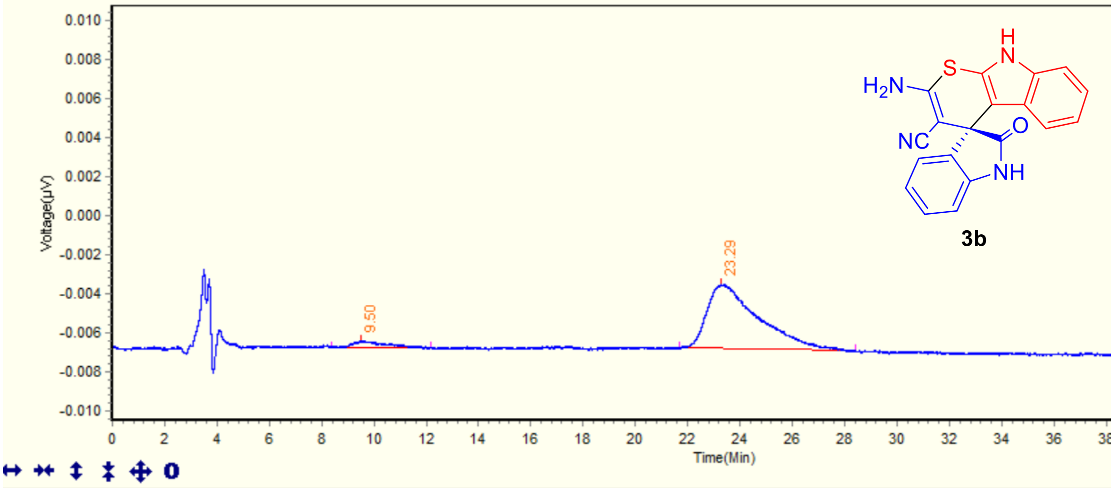

Integration Result | Calculation Result | TimeTable

No.	Retention Time	Peak Area	Peak Height	Peak Area(%)	Peak Width	
1	9.50	11710	158	4.96%	3.802	BB
2	23.29	224501	1647	95.04%	6.731	BB
Total		236,211	1,805	100.00%		

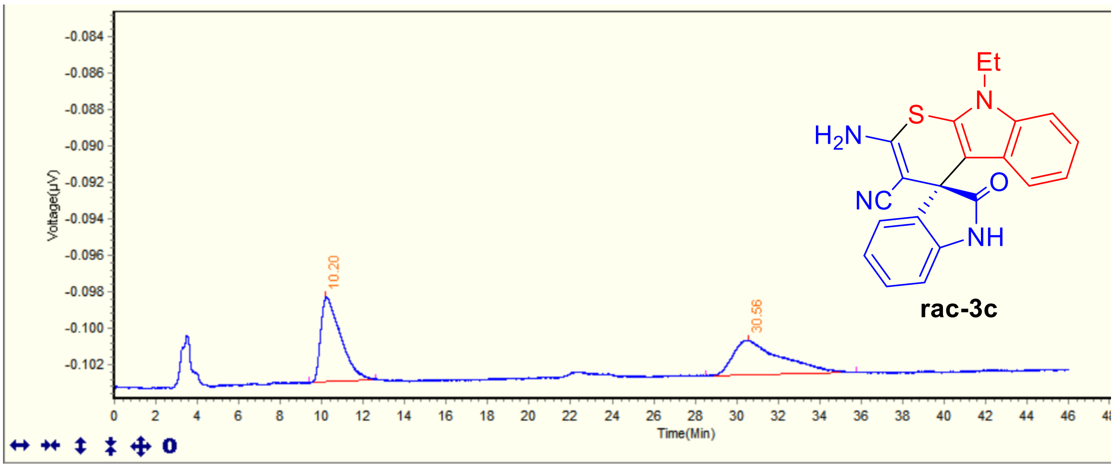

Integration Result | Calculation Result | TimeTable

No.	Retention Time	Peak Area	Peak Height	Peak Area(%)	Peak Width	Peak Ty
1	10.20	155339	2318	50.88%	3.208	BB
2	30.56	149974	951	49.12%	7.263	BB
Total		305,313	3,269	100.00%		

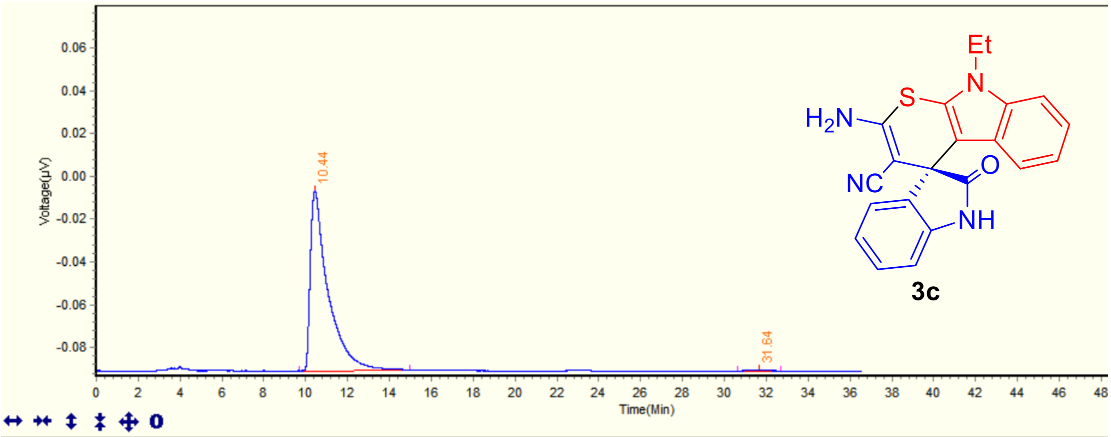

Integration Result | Calculation Result | TimeTable

No.	Retention Time	Peak Area	Peak Height	Peak Area(%)	Peak Width	Peak Type
1	10.44	2467861	41978	99.50%	5.258	BB
2	31.64	12491	177	0.50%	2.055	BB
Total		2,480,352	42,155	100.00%		

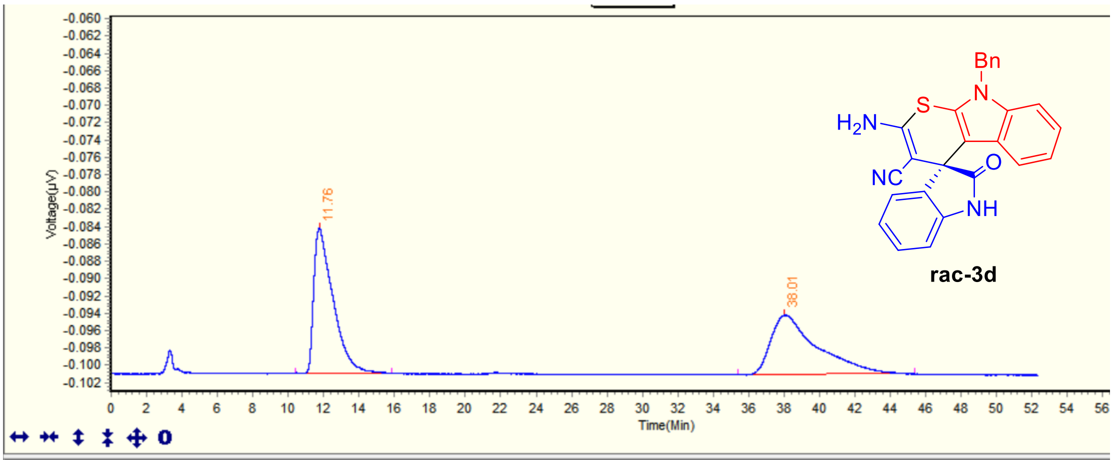

No.	Retention Time	Peak Area	Peak Height	Peak Area(%)	Peak Width	Peak Type
1	11.76	631808	8357	50.32%	5.441	BB
2	38.01	623748	3429	49.68%	9.986	BB
Total		1,255,556	11,786	100.00%		

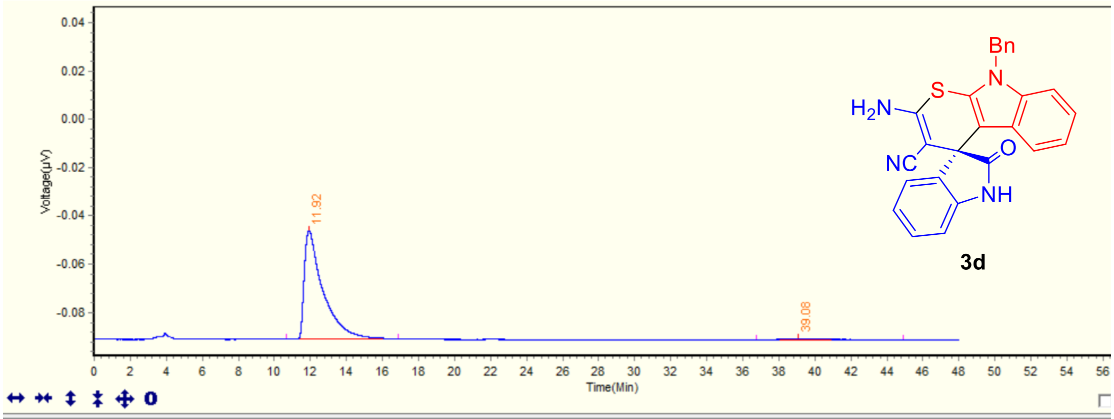

No.	Retention Time	Peak Area	Peak Height	Peak Area(%)	Peak Width	Peak Type
1	11.92	1625194	22446	97.18%	6.208	BB
2	39.08	47242	315	2.82%	8.153	BB
Total		1,672,436	22,761	100.00%		

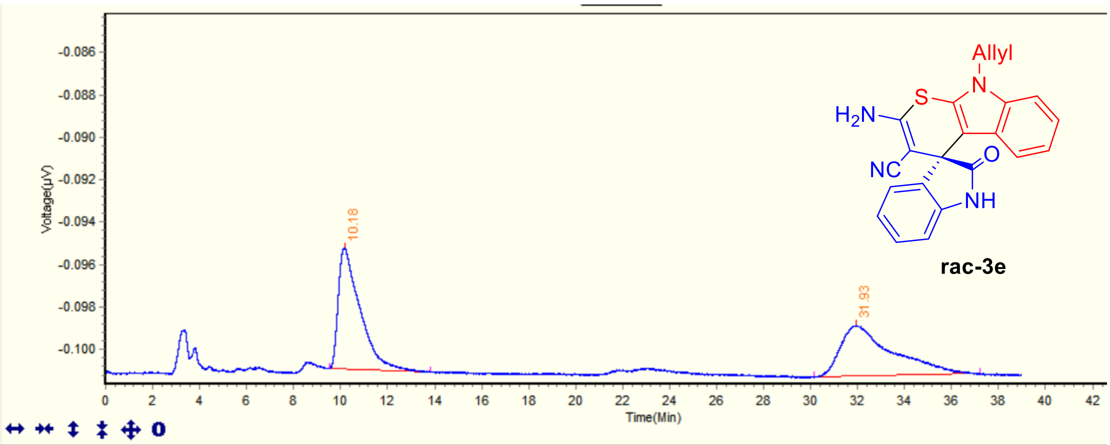

Integration Result | Calculation Result | TimeTable

No.	Retention Time	Peak Area	Peak Height	Peak Area(%)	Peak Width	Peak Type
1	10.18	182439	2842	50.30%	4.299	BB
2	31.93	180293	1189	49.70%	7.063	BB
Total		362,732	4,031	100.00%		

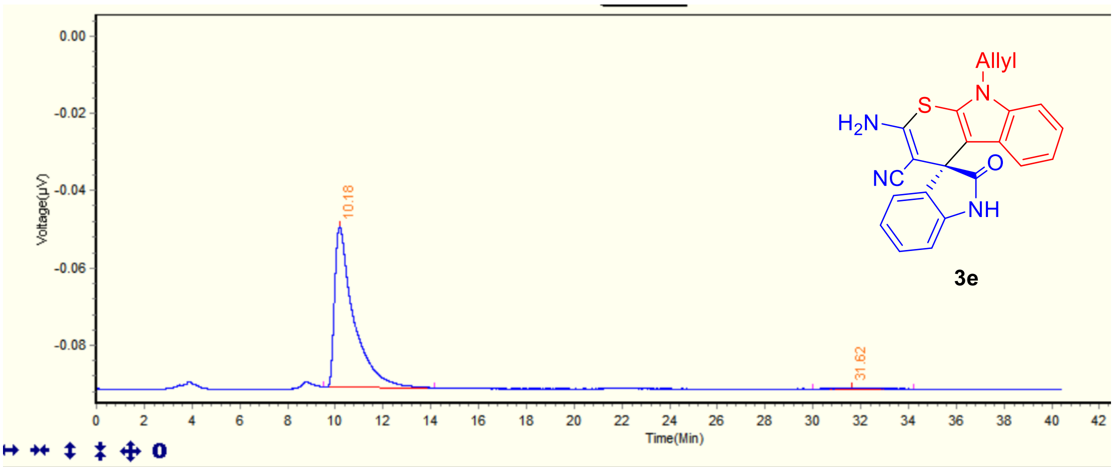

Integration Result | Calculation Result | TimeTable

No.	Retention Time	Peak Area	Peak Height	Peak Area(%)	Peak Width	Peak Typ
1	10.18	1145411	20716	99.14%	4.647	BB
2	31.62	9943	117	0.86%	4.224	BB
Total		1,155,354	20,833	100.00%		

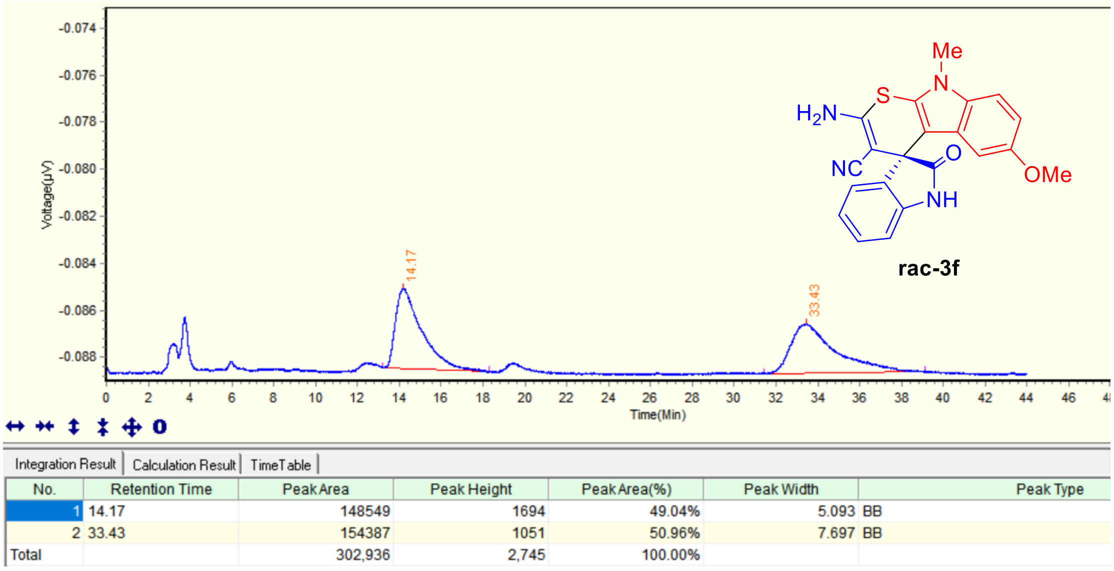

No.	Retention Time	Peak Area	Peak Height	Peak Area(%)	Peak Width	Peak Type
1	14.17	148549	1694	49.04%	5.093	BB
2	33.43	154387	1051	50.96%	7.697	BB
Total		302,936	2,745	100.00%		

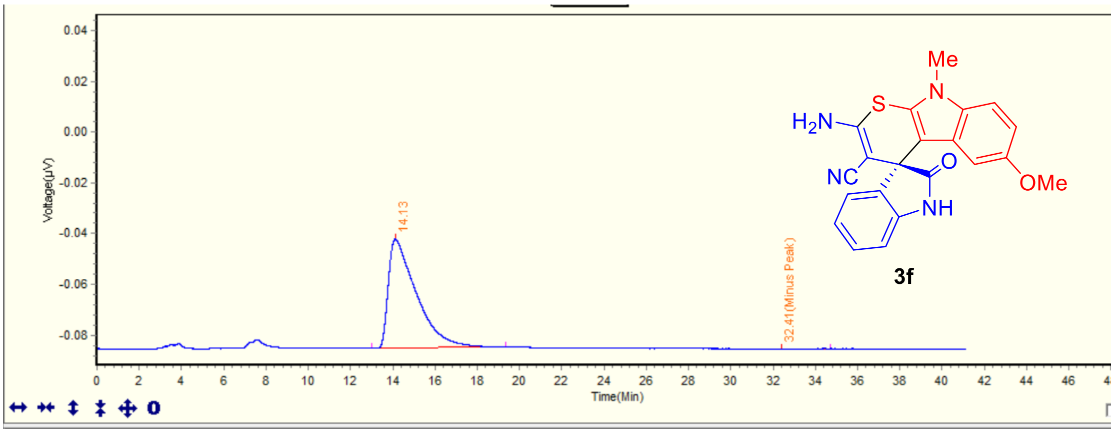

No.	Retention Time	Peak Area	Peak Height	Peak Area(%)	Peak Width	Peak Type
1	14.13	1962042	21410	99.99%	6.34	BB
2	32.41	186	23	0.01%	2.311	BB
Total		1,962,228	21,433	100.00%		

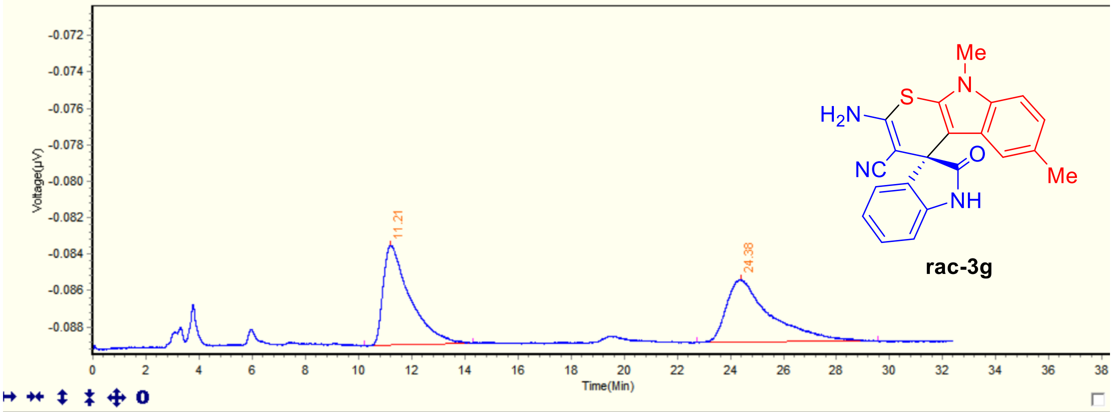

Integration Result	Calculation Result	TimeTable				
No.	Retention Time	Peak Area	Peak Height	Peak Area(%)	Peak Width	Peak Type
1	11.21	190280	2728	49.01%	4.088	BB
2	24.38	198000	1711	50.99%	6.814	BB
Total		388,280	4,439	100.00%		

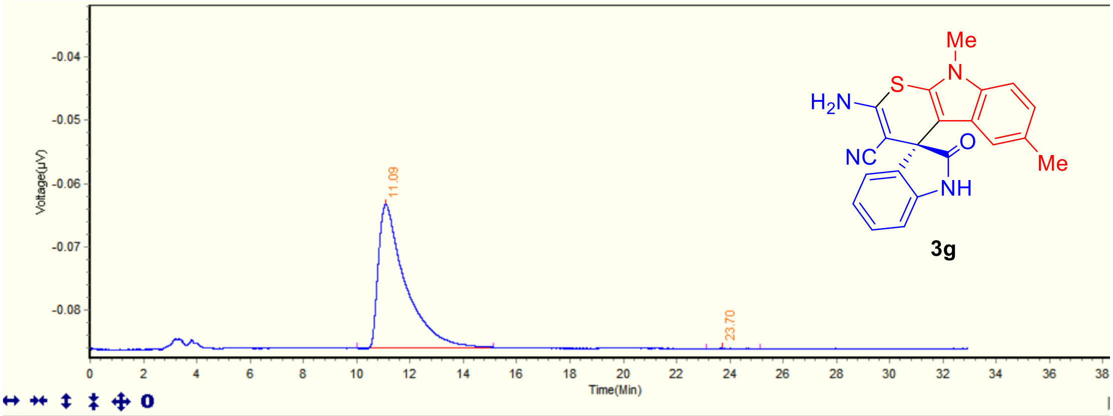

Integration Result	Calculation Result	TimeTable				
No.	Retention Time	Peak Area	Peak Height	Peak Area(%)	Peak Width	Peak Type
1	11.09	802161	11402	99.91%	5.112	BB
2	23.70	702	28	0.09%	2.019	BB
Total		802,863	11,430	100.00%		

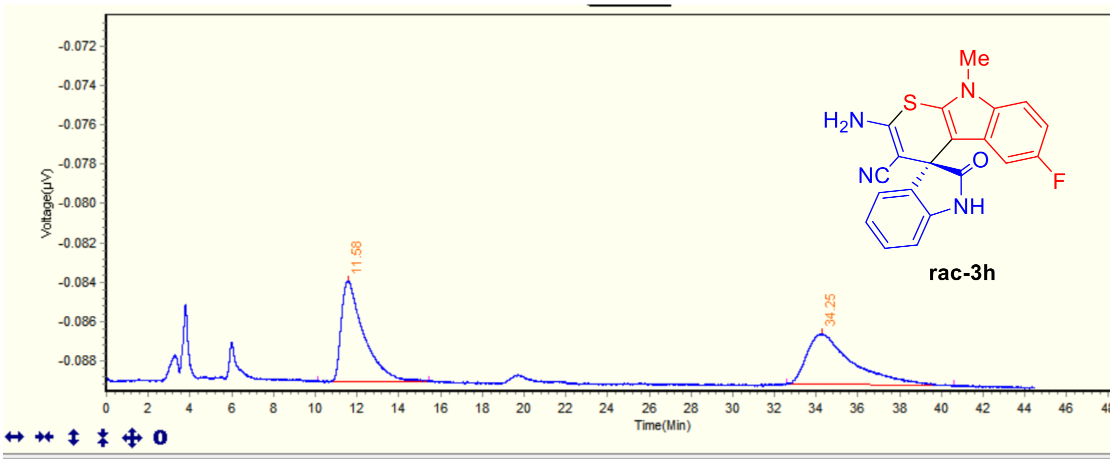

Integration Result | Calculation Result | TimeTable

No.	Retention Time	Peak Area	Peak Height	Peak Area(%)	Peak Width	Peak Type
1	11.58	190085	2557	49.63%	5.323	BB
2	34.25	192947	1279	50.37%	8.013	BB
Total		383,032	3,836	100.00%		

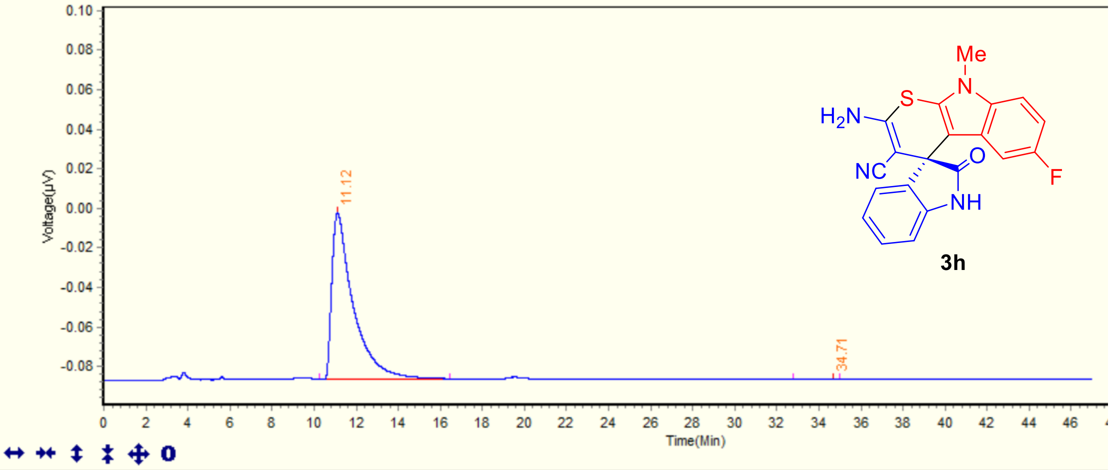

Integration Result | Calculation Result | TimeTable

No.	Retention Time	Peak Area	Peak Height	Peak Area(%)	Peak Width	Pea
1	11.12	2951274	41959	99.97%	6.199	BB
2	34.71	879	29	0.03%	2.21	BB
Total		2,952,153	41,988	100.00%		

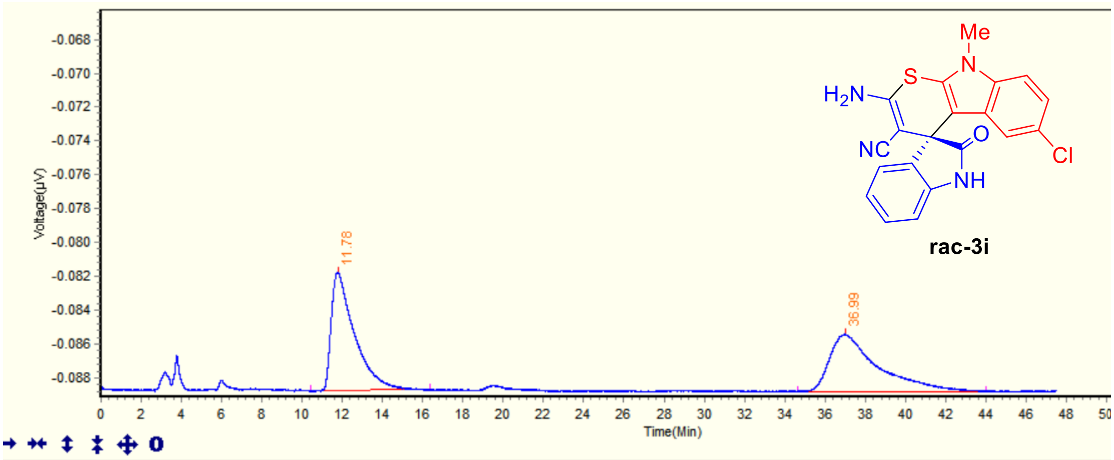

Integration Result	Calculation Result	TimeTable				
No.	Retention Time	Peak Area	Peak Height	Peak Area(%)	Peak Width	Peak Type
1	11.78	283554	3508	50.32%	5.93	BB
2	36.99	279952	1688	49.68%	9.364	BB
Total		563,506	5,196	100.00%		

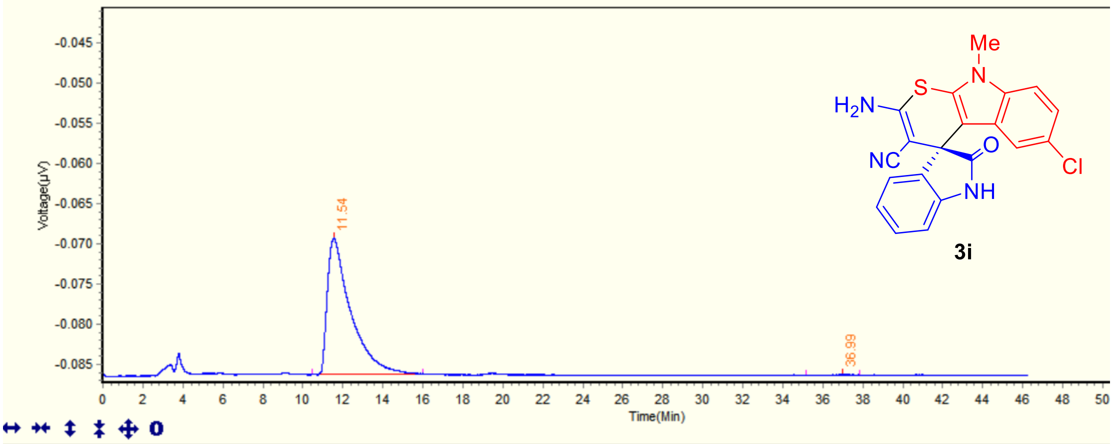

Integration Result	Calculation Result	TimeTable				
No.	Retention Time	Peak Area	Peak Height	Peak Area(%)	Peak Width	Peak Type
1	11.54	683520	8445	99.79%	5.537	BB
2	36.99	1444	35	0.21%	2.677	BB
Total		684,964	8,480	100.00%		

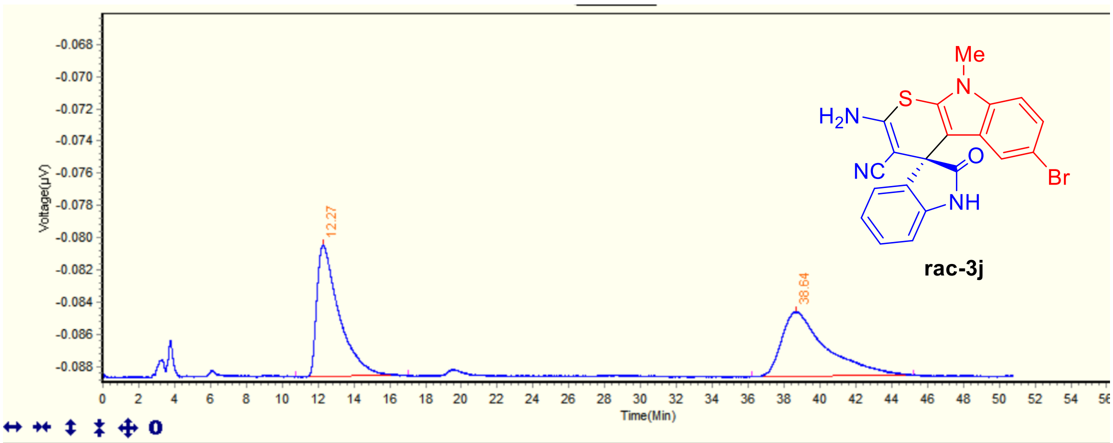

Integration Result | Calculation Result | TimeTable

No.	Retention Time	Peak Area	Peak Height	Peak Area(%)	Peak Width	Peak Type
1	12.27	351370	4066	50.54%	6.278	BB
2	38.64	343859	1992	49.46%	9.016	BB
Total		695,229	6,058	100.00%		

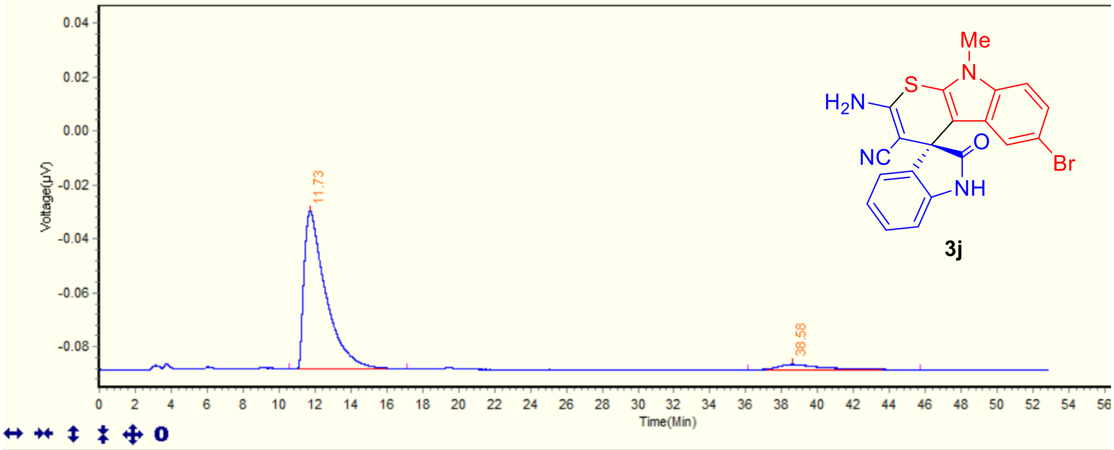

Integration Result | Calculation Result | TimeTable

No.	Retention Time	Peak Area	Peak Height	Peak Area(%)	Peak Width	Peak Type
1	11.73	2467362	29271	93.70%	6.559	BB
2	38.58	165974	982	6.30%	9.598	BB
Total		2,633,336	30,253	100.00%		

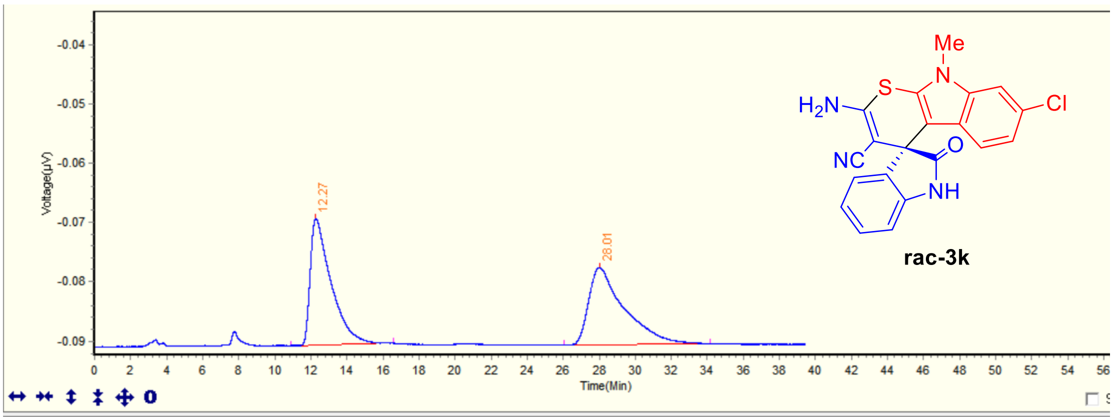

Integration Result | Calculation Result | TimeTable

No.	Retention Time	Peak Area	Peak Height	Peak Area(%)	Peak Width	Peak Type
1	12.27	852278	10651	49.08%	5.693	BB
2	28.01	884280	6497	50.92%	8.106	BB
Total		1,736,558	17,148	100.00%		

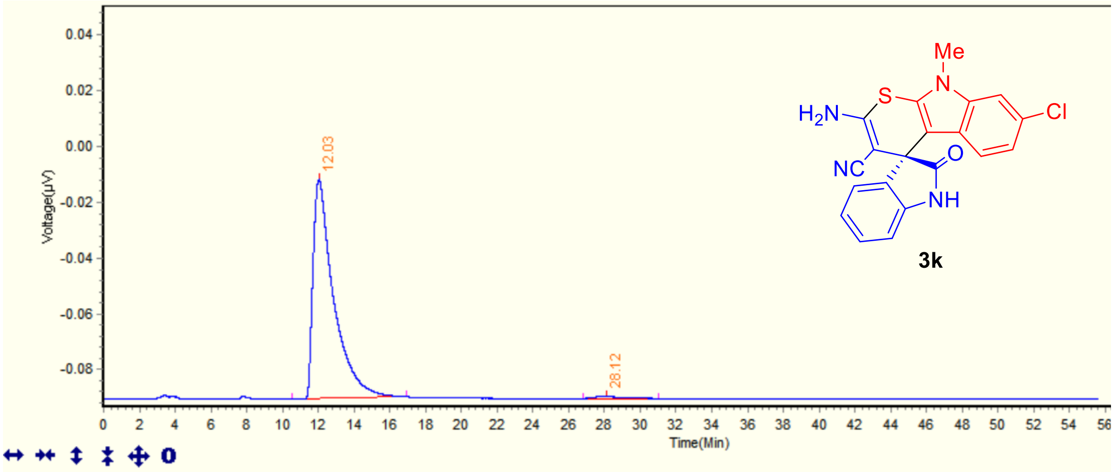

Integration Result | Calculation Result | TimeTable

No.	Retention Time	Peak Area	Peak Height	Peak Area(%)	Peak Width	Pea
1	12.03	3141571	39243	98.71%	6.393	BB
2	28.12	40947	381	1.29%	4.214	BB
Total		3,182,518	39,624	100.00%		

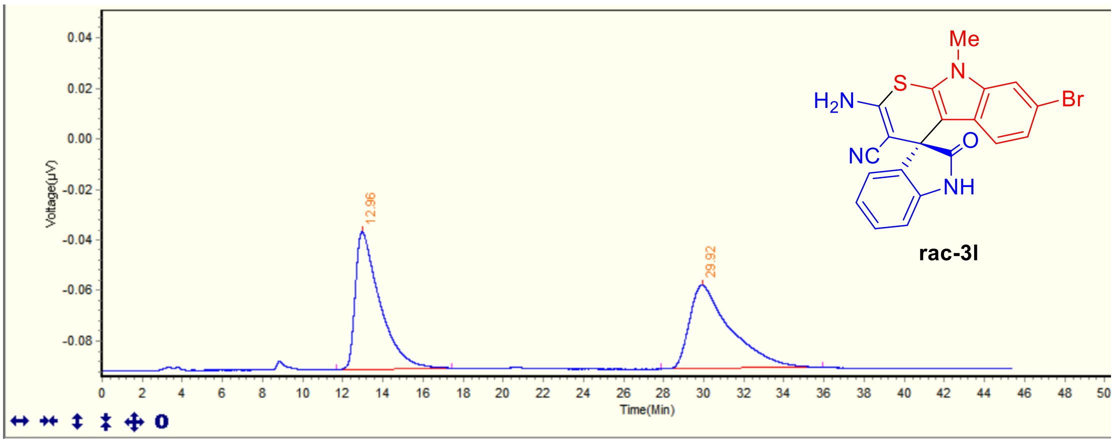

Integration Result | Calculation Result | TimeTable

No.	Retention Time	Peak Area	Peak Height	Peak Area(%)	Peak Width	Peak Type
1	12.96	2309816	27240	49.43%	5.752	BB
2	29.92	2362839	16479	50.57%	8.063	BB
Total		4,672,655	43,719	100.00%		

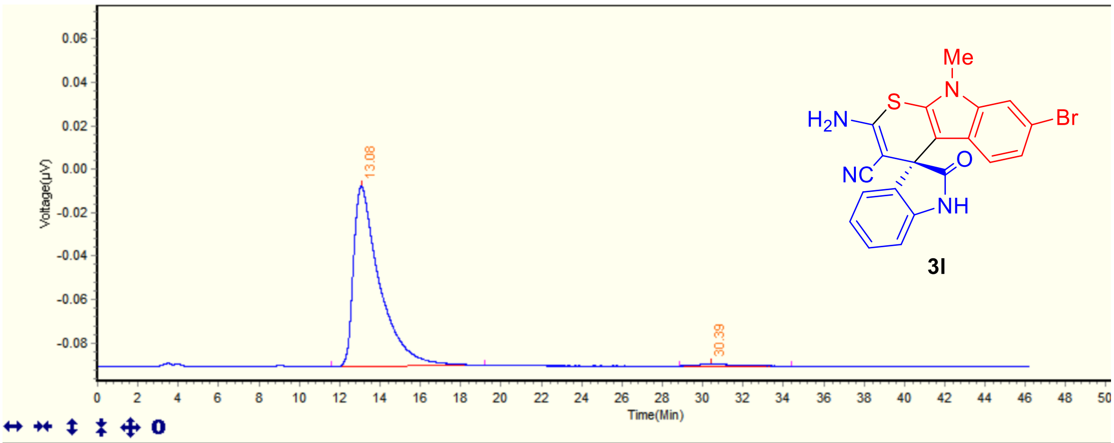

Integration Result | Calculation Result | TimeTable

No.	Retention Time	Peak Area	Peak Height	Peak Area(%)	Peak Width	Peak Type
1	13.08	3738379	41435	98.43%	7.604	BB
2	30.39	59574	465	1.57%	5.553	BB
Total		3,797,953	41,900	100.00%		

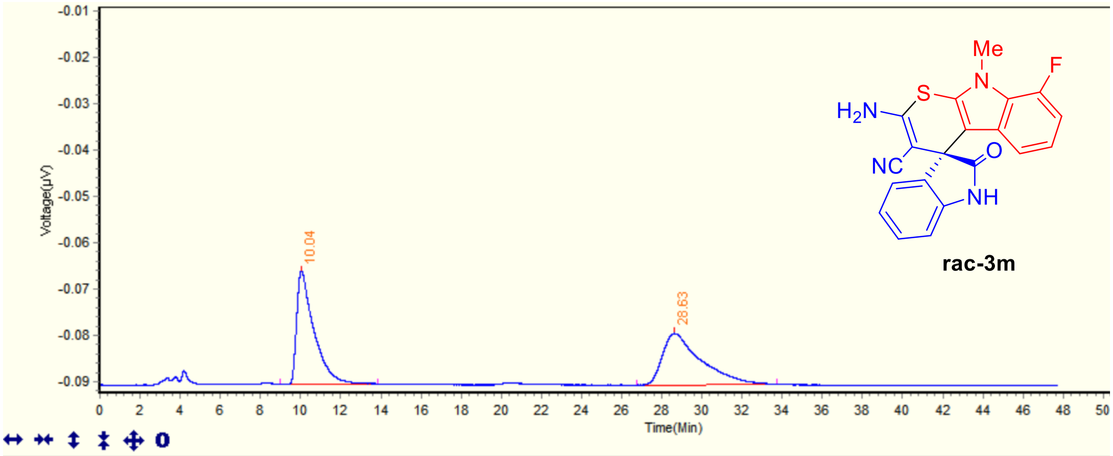

Integration Result	Calculation Result	TimeTable				
No.	Retention Time	Peak Area	Peak Height	Peak Area(%)	Peak Width	Peak Typ
1	10.04	727894	12219	49.74%	4.863	BB
2	28.63	735359	5542	50.26%	6.984	BB
Total		1,463,253	17,761	100.00%		

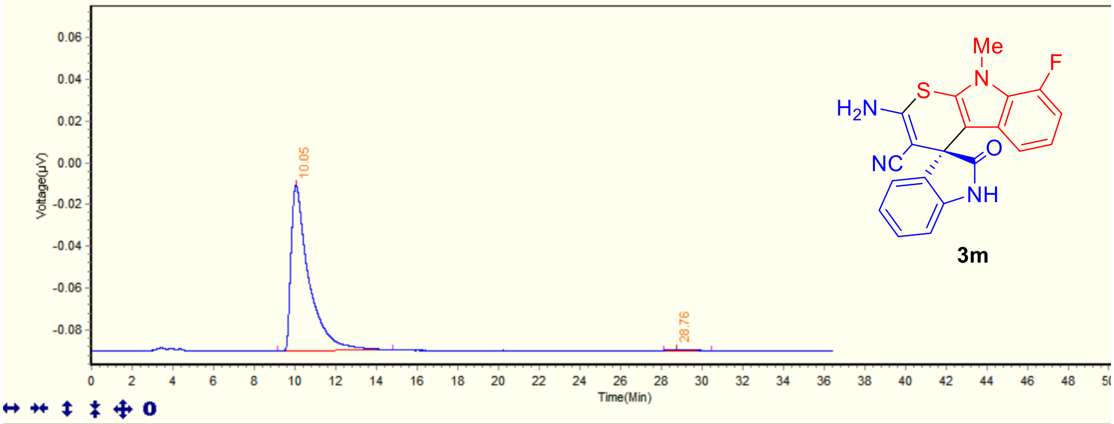

Integration Result	Calculation Result	TimeTable				
No.	Retention Time	Peak Area	Peak Height	Peak Area(%)	Peak Width	Peak Type
1	10.05	2401207	39730	99.51%	5.658	BB
2	28.76	11795	175	0.49%	2.343	BB
Total		2,413,002	39,905	100.00%		

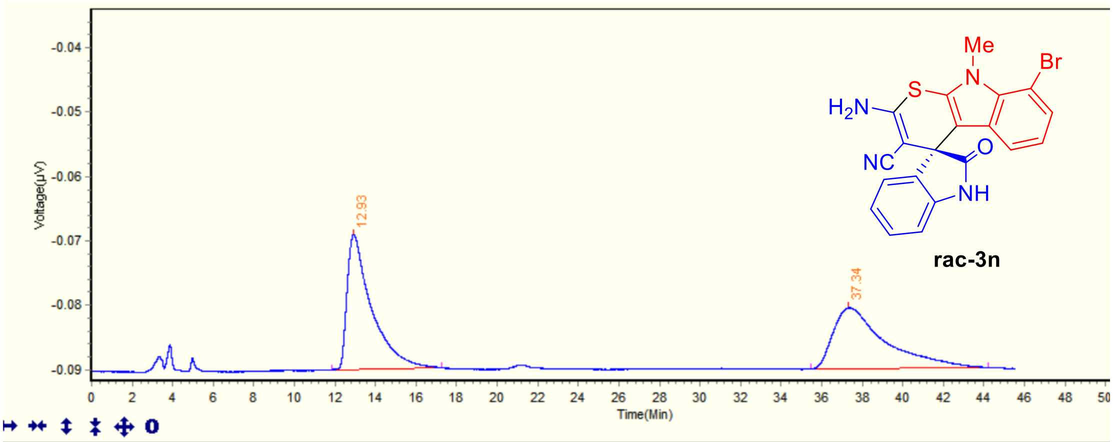

No.	Retention Time	Peak Area	Peak Height	Peak Area(%)	Peak Width	Peak Type
1	12.93	866514	10443	50.88%	5.415	BB
2	37.34	836513	4712	49.12%	8.748	BB
Total		1,703,027	15,155	100.00%		

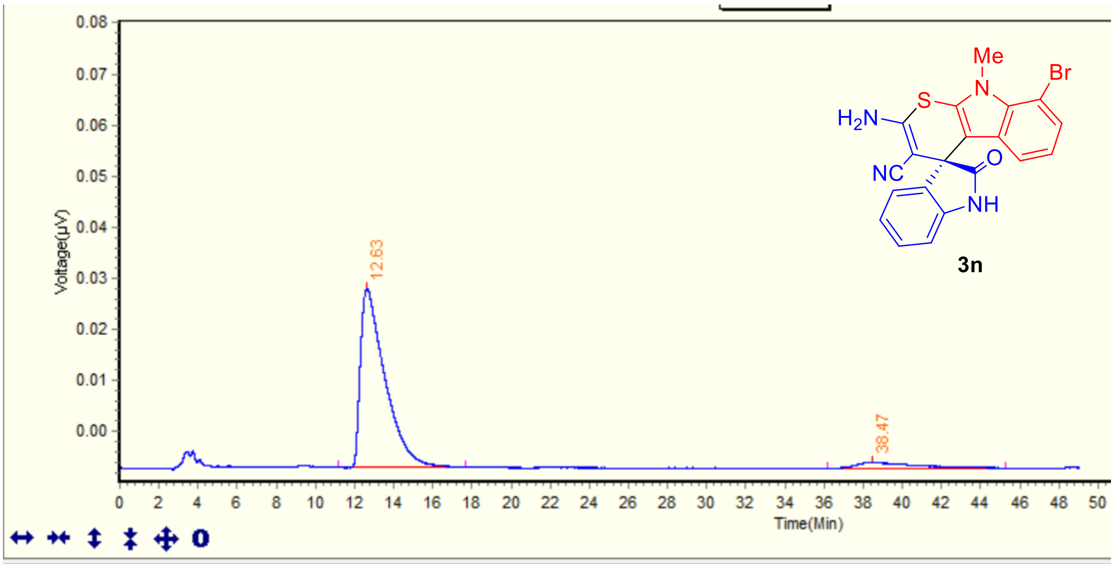

No.	Retention Time	Peak Area	Peak Height	Peak Area(%)	Peak Width	
1	12.63	1522860	17546	92.67%	6.5	BB
2	38.47	120482	577	7.33%	9.112	BB
Total		1,643,342	18,123	100.00%		

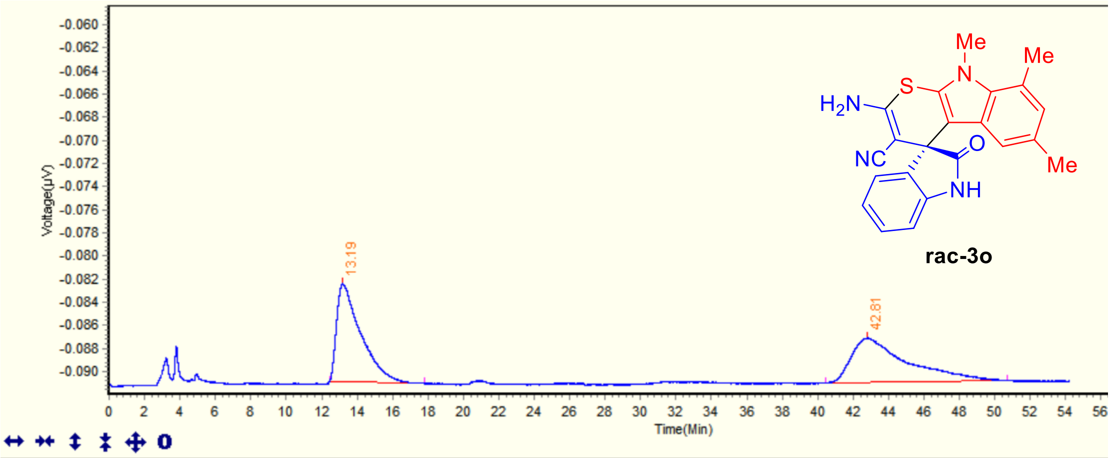

Integration Result | Calculation Result | TimeTable

No.	Retention Time	Peak Area	Peak Height	Peak Area(%)	Peak Width	Peak Ty
1	13.19	402191	4238	49.42%	5.341	BB
2	42.81	411649	1910	50.58%	10.255	BB
Total		813,840	6,148	100.00%		

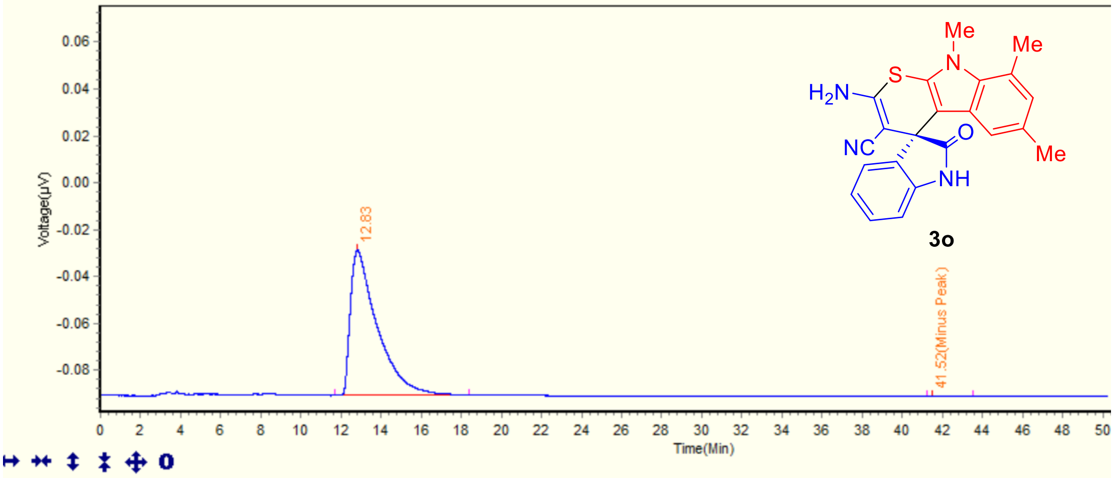

Integration Result | Calculation Result | TimeTable

No.	Retention Time	Peak Area	Peak Height	Peak Area(%)	Peak Width	P
1	12.83	2882257	30944	99.91%	6.697	BB
2	41.52	2493	41	0.09%	2.298	BB
Total		2.884.750	30.985	100.00%		

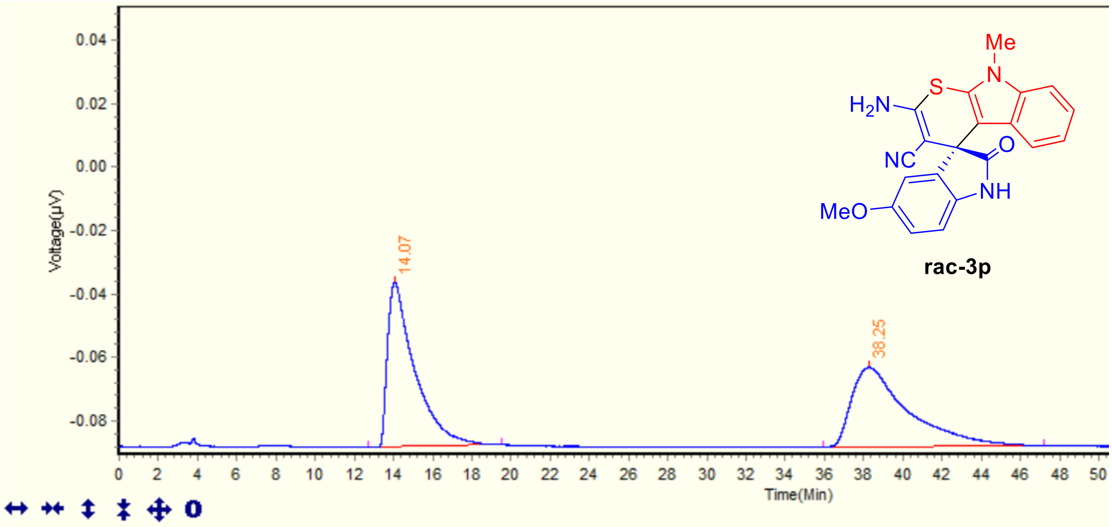

Integration Result | Calculation Result | TimeTable

No.	Retention Time	Peak Area	Peak Height	Peak Area(%)	Peak Width	
1	14.07	2494171	25842	50.10%	6.754	BB
2	38.25	2483732	12468	49.90%	11.22	BB
Total		4,977,903	38,310	100.00%		

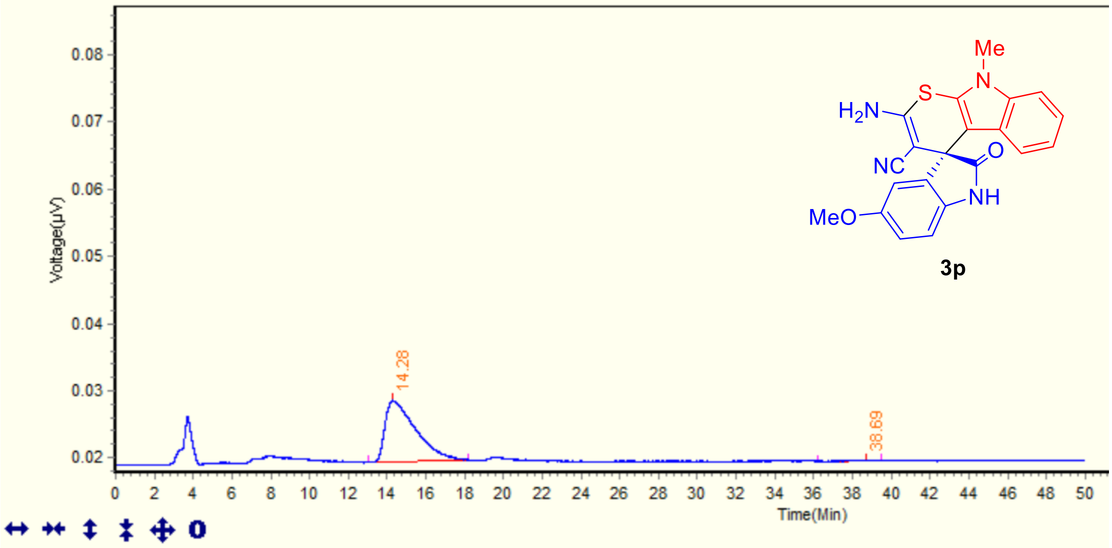

Integration Result | Calculation Result | TimeTable

No.	Retention Time	Peak Area	Peak Height	Peak Area(%)	Peak Width	
1	14.28	494823	4555	99.71%	5.15	BB
2	38.69	1463	60	0.29%	3.278	BB
Total		496,286	4,615	100.00%		

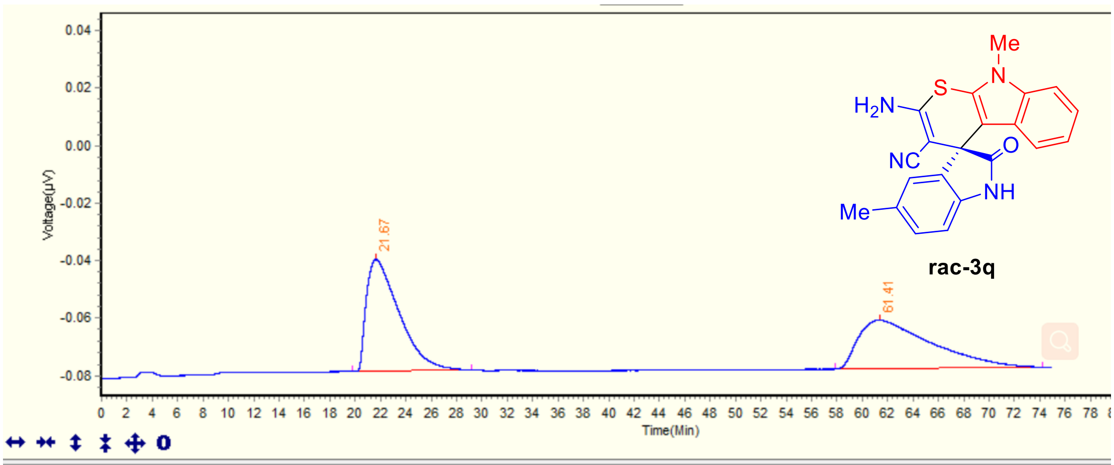

Integration Result | Calculation Result | TimeTable

No.	Retention Time	Peak Area	Peak Height	Peak Area(%)	Peak Width	Peak Type
1	21.67	3492419	19378	50.93%	9.399	BB
2	61.41	3364759	8433	49.07%	16.352	BB
Total		6,857,178	27,811	100.00%		

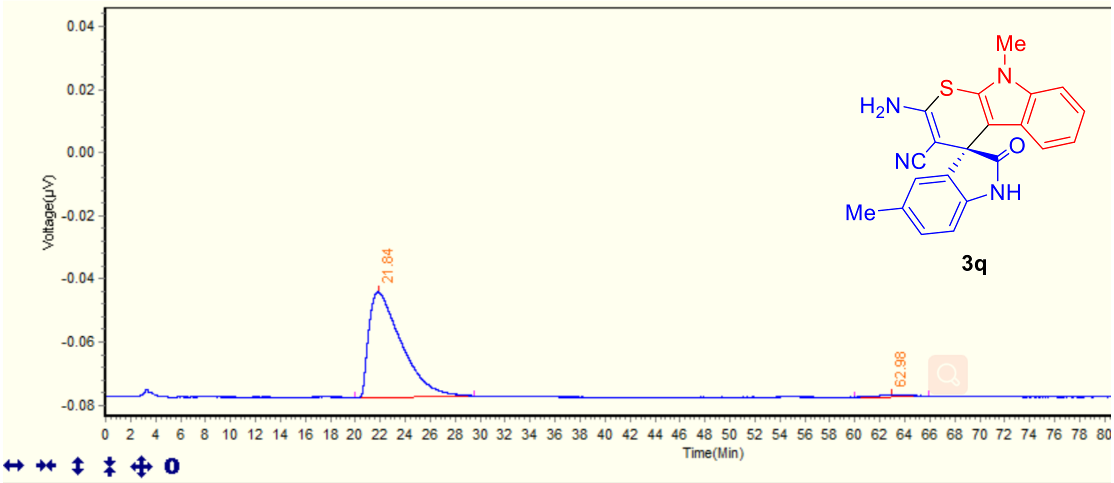

Integration Result | Calculation Result | TimeTable

No.	Retention Time	Peak Area	Peak Height	Peak Area(%)	Peak Width	
1	21.84	2995574	16600	98.88%	9.553	BB
2	62.98	33841	213	1.12%	5.943	BB
Total		3,029,415	16,813	100.00%		

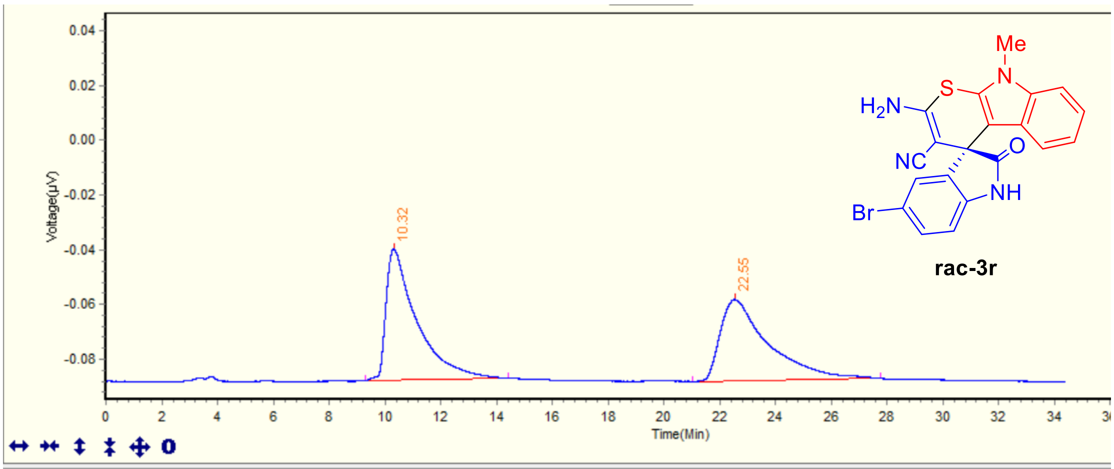

Integration Result | Calculation Result | TimeTable

No.	Retention Time	Peak Area	Peak Height	Peak Area(%)	Peak Width	Peak Ty[illegible]
1	10.32	1832128	23972	50.88%	5.122	BB
2	22.55	1769018	14799	49.12%	6.739	BB
Total		3,601,146	38,771	100.00%		

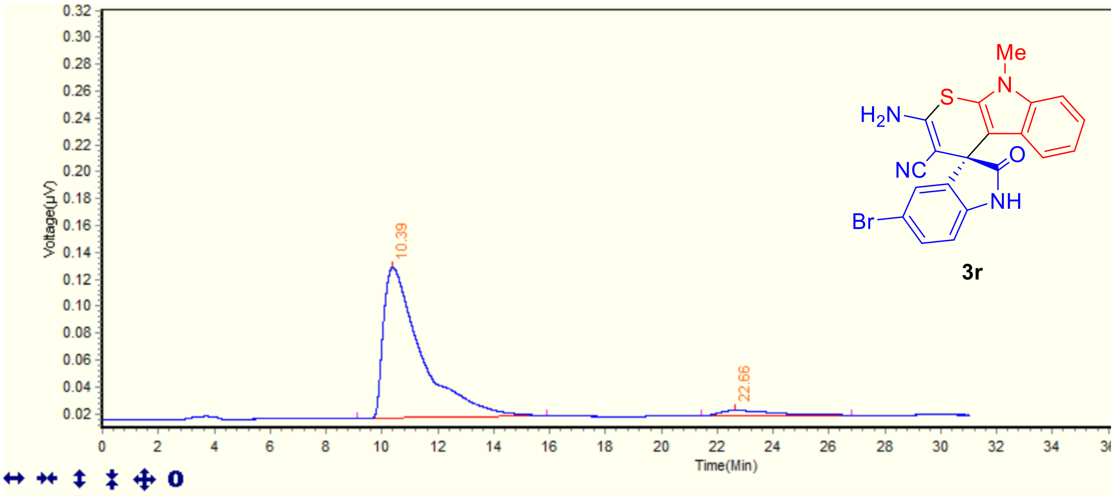

Integration Result | Calculation Result | TimeTable

No.	Retention Time	Peak Area	Peak Height	Peak Area(%)	Peak Width	
1	10.39	5254933	55912	94.89%	6.787	BB
2	22.66	282946	2058	5.11%	5.373	BB
Total		5,537,879	57,970	100.00%		

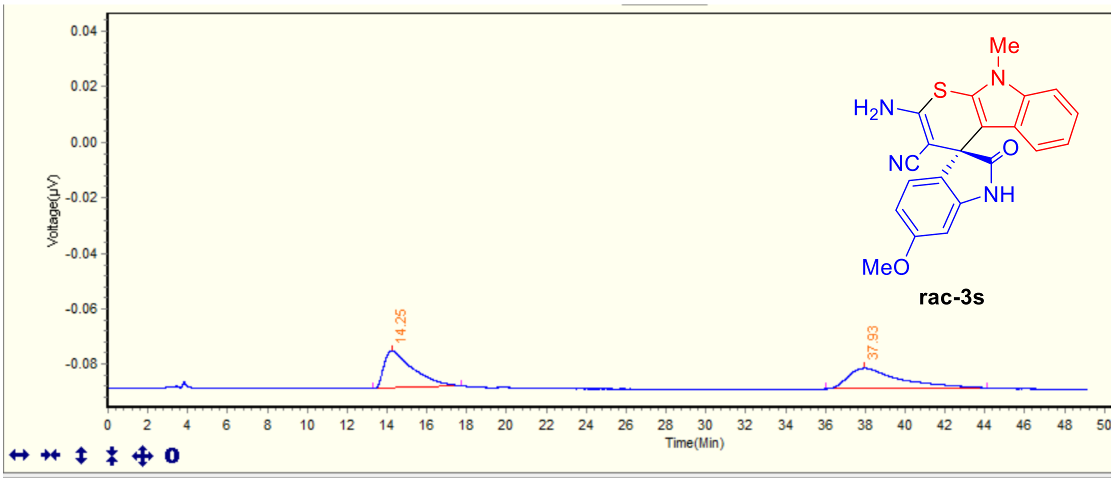

Integration Result | Calculation Result | TimeTable

No.	Retention Time	Peak Area	Peak Height	Peak Area(%)	Peak Width	Peak
1	14.25	681524	6753	50.74%	4.431	BB
2	37.93	661576	3664	49.26%	8.091	BB
Total		1,343,100	10,417	100.00%		

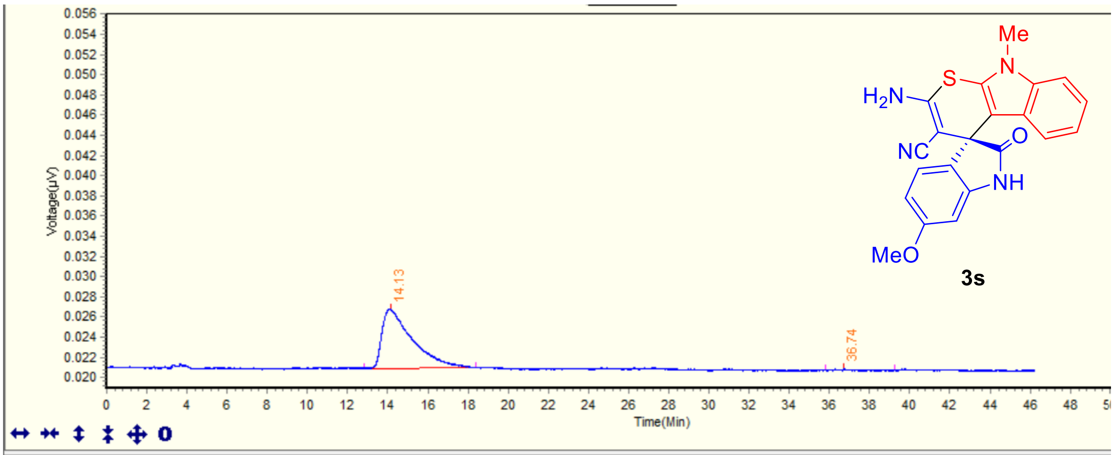

Integration Result | Calculation Result | TimeTable

No.	Retention Time	Peak Area	Peak Height	Peak Area(%)	Peak Width	Peak Type
1	14.13	306951	2927	99.74%	5.594	BB
2	36.74	791	55	0.26%	3.427	BB
Total		307,742	2,982	100.00%		

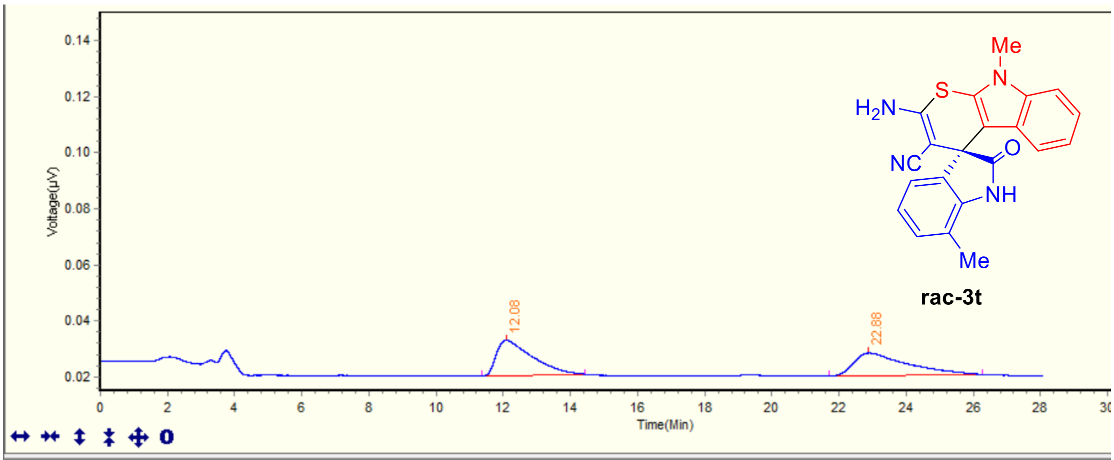

No.	Retention Time	Peak Area	Peak Height	Peak Area(%)	Peak Width	Peak Type
1	12.08	464554	6296	50.92%	3.069	BB
2	22.88	447824	4045	49.08%	4.567	BB
Total		912,378	10,341	100.00%		

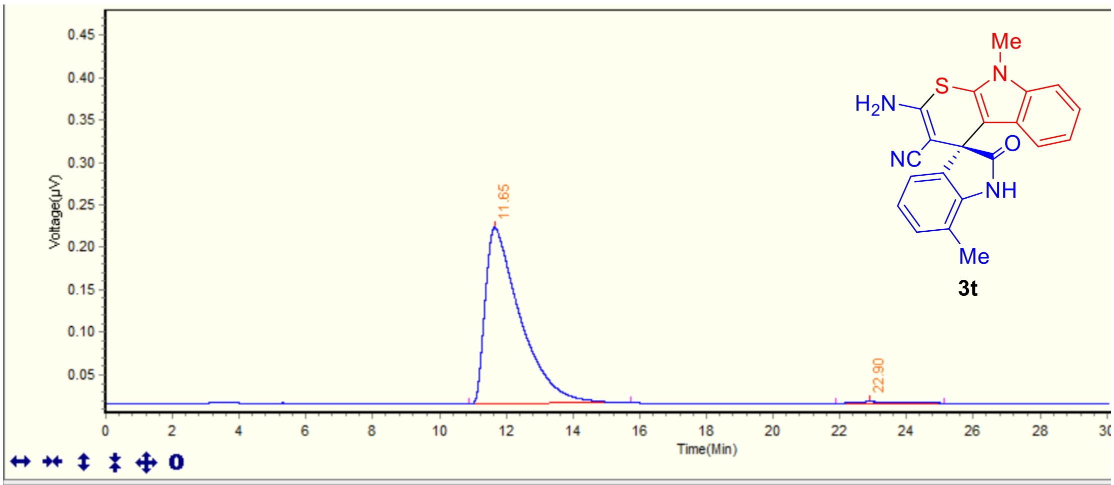

No.	Retention Time	Peak Area	Peak Height	Peak Area(%)	Peak Width	P
1	11.65	7611570	104005	98.66%	4.853	BB
2	22.90	103026	1063	1.34%	3.248	BB
Total		7,714,596	105,068	100.00%		

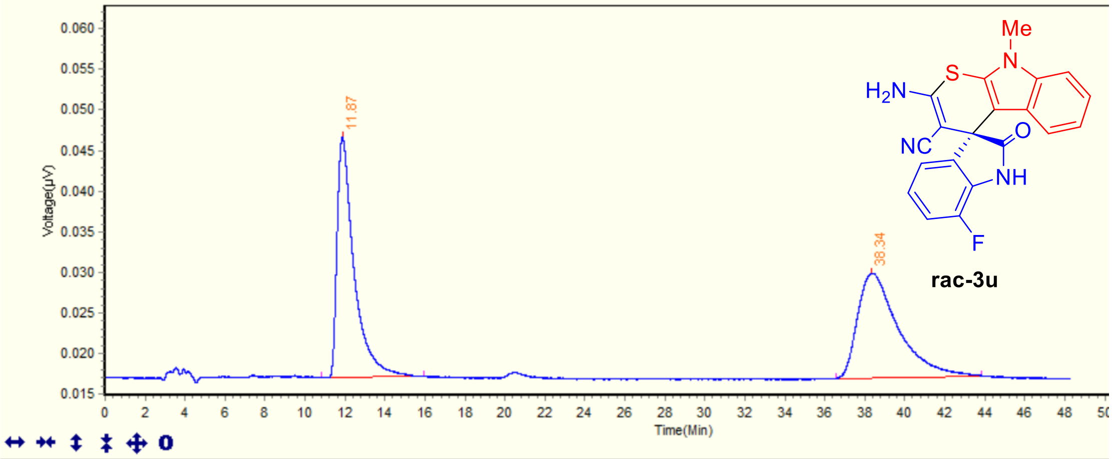

Integration Result | Calculation Result | TimeTable

No.	Retention Time	Peak Area	Peak Height	Peak Area(%)	Peak Width	Peak T
1	11.87	872320	14799	49.06%	5.123	BB
2	38.34	905847	6439	50.94%	7.273	BB
Total		1,778,167	21,238	100.00%		

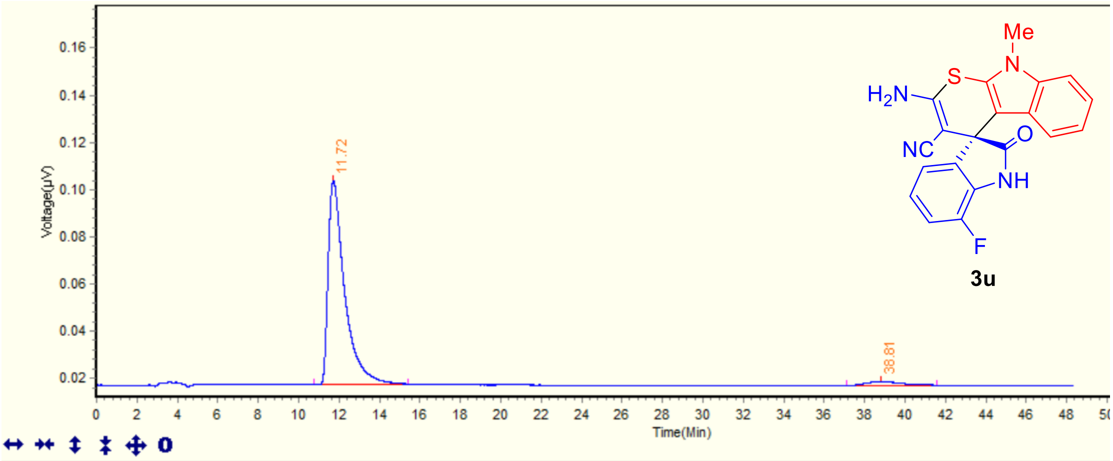

Integration Result | Calculation Result | TimeTable

No.	Retention Time	Peak Area	Peak Height	Peak Area(%)	Peak Width	Peak T
1	11.72	2445593	43258	96.06%	4.653	BB
2	38.81	100363	829	3.94%	4.466	BB
Total		2,545,956	44,087	100.00%		

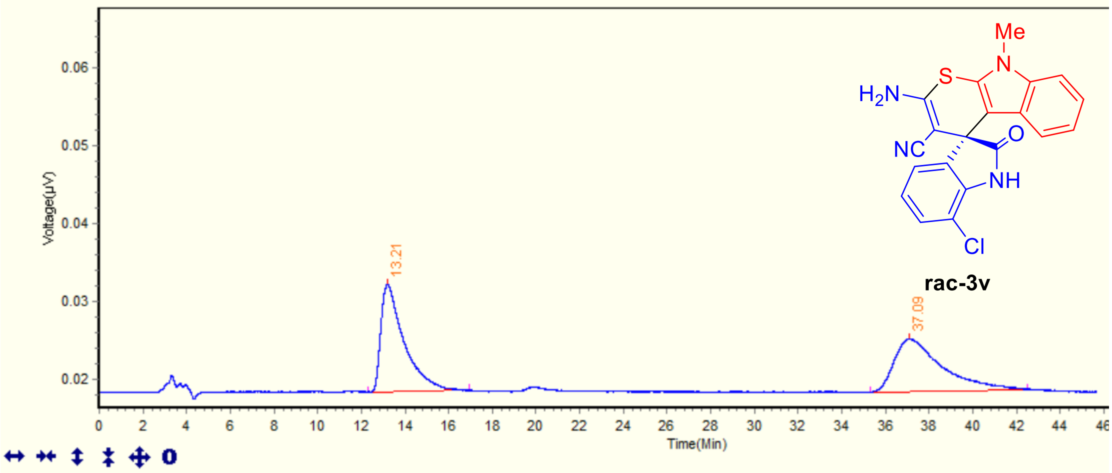

Integration Result	Calculation Result	TimeTable				
No.	Retention Time	Peak Area	Peak Height	Peak Area(%)	Peak Width	Pe
1	13.21	506047	6850	49.74%	4.607	BB
2	37.09	511394	3370	50.26%	7.193	BB
Total		1,017,441	10,220	100.00%		

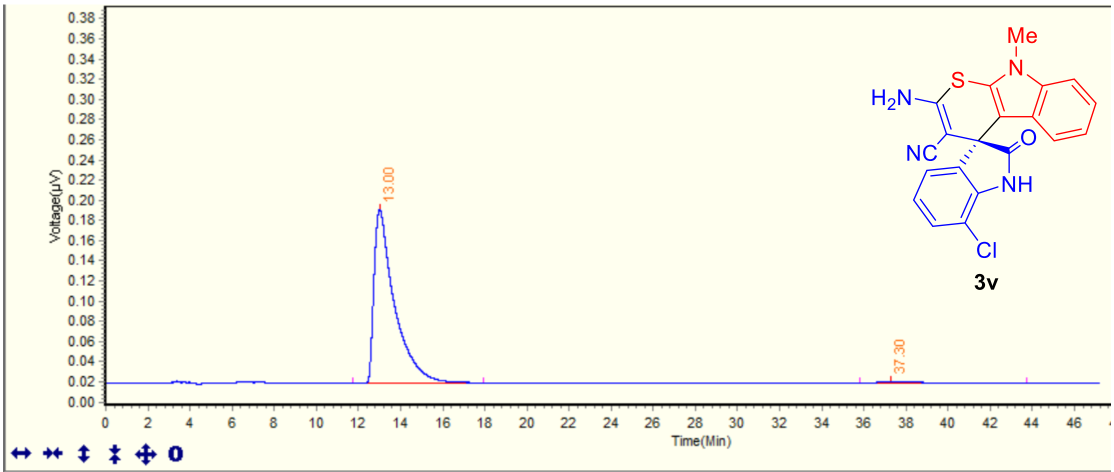

Integration Result	Calculation Result	TimeTable				
No.	Retention Time	Peak Area	Peak Height	Peak Area(%)	Peak Width	Pe
1	13.00	5772428	85793	97.10%	6.203	BB
2	37.30	172242	1104	2.90%	7.923	BB
Total		5,944,670	86,897	100.00%		

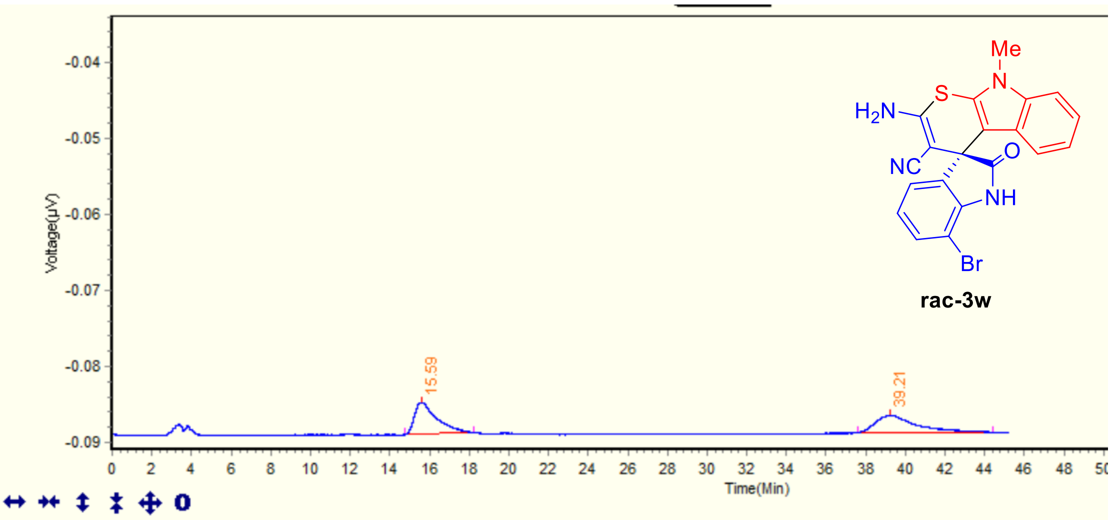

Integration Result | Calculation Result | TimeTable

No.	Retention Time	Peak Area	Peak Height	Peak Area(%)	Peak Width	
1	15.59	162598	2082	50.27%	3.428	BB
2	39.21	160848	1122	49.73%	6.798	BB
Total		323,446	3,204	100.00%		

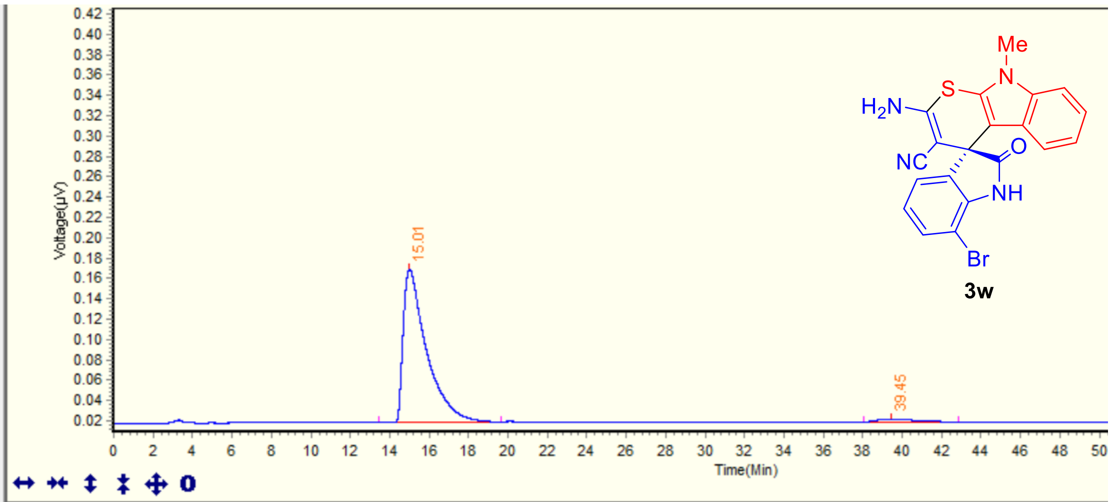

Integration Result | Calculation Result | TimeTable

No.	Retention Time	Peak Area	Peak Height	Peak Area(%)	Peak Width	
1	15.01	6177198	75240	96.91%	6.202	BB
2	39.45	197236	1534	3.09%	4.808	BB
Total		6,374,434	76,774	100.00%		

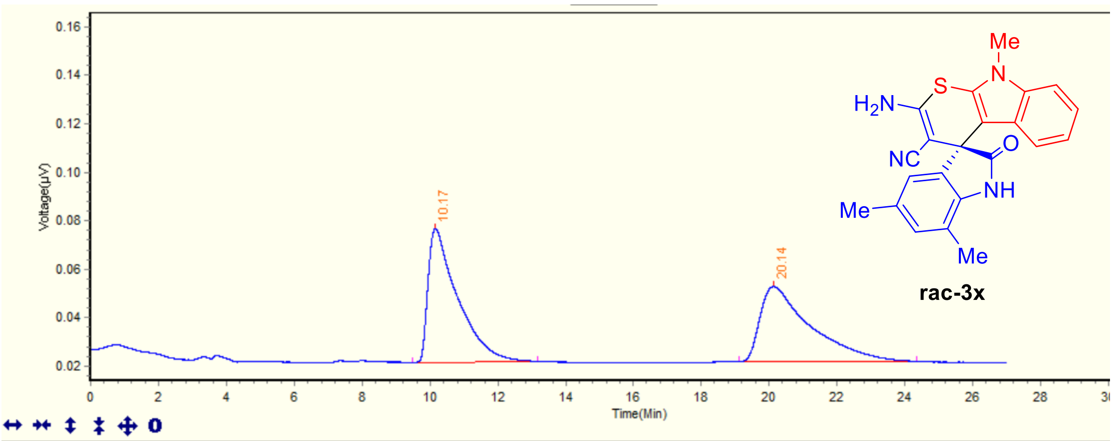

Integration Result | Calculation Result | TimeTable

No.	Retention Time	Peak Area	Peak Height	Peak Area(%)	Peak Width	Peak Type
1	10.17	1658695	27559	50.92%	3.689	BB
2	20.14	1598964	15510	49.08%	5.234	BB
Total		3,257,659	43,069	100.00%		

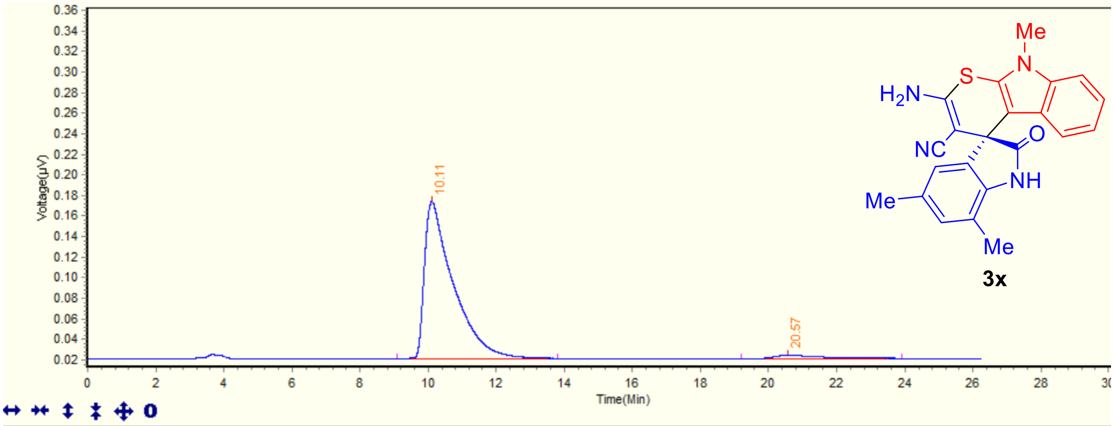

Integration Result | Calculation Result | TimeTable

No.	Retention Time	Peak Area	Peak Height	Peak Area(%)	Peak Width	Peak Type
1	10.11	4469850	76336	97.04%	4.715	BB
2	20.57	136541	1559	2.96%	4.714	BB
Total		4,606,391	77,895	100.00%		

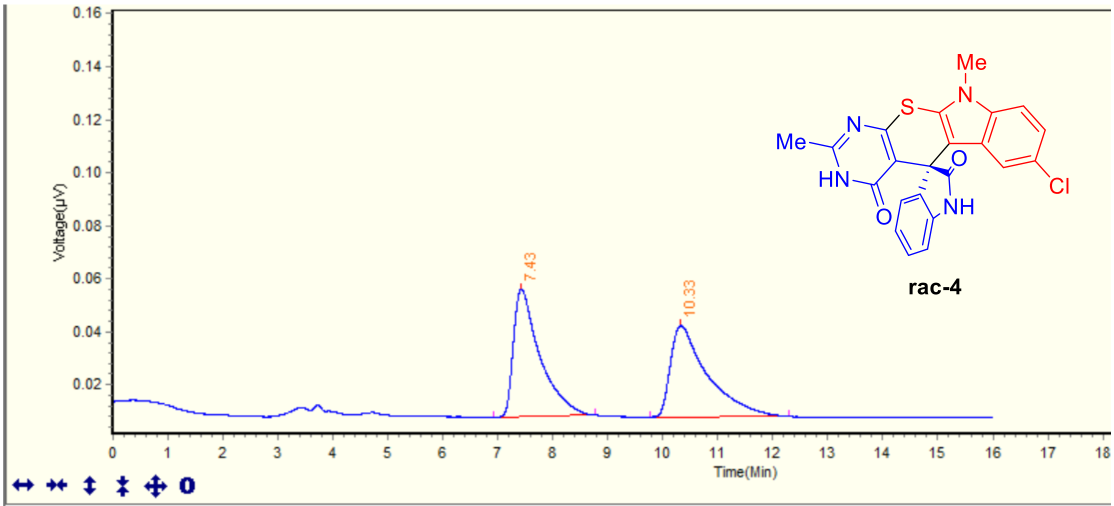

No.	Retention Time	Peak Area	Peak Height	Peak Area(%)	Peak Width
1	7.43	802188	24050	50.07%	1.853 BB
2	10.33	799966	17187	49.93%	2.539 BB
Total		1,602,154	41,237	100.00%	

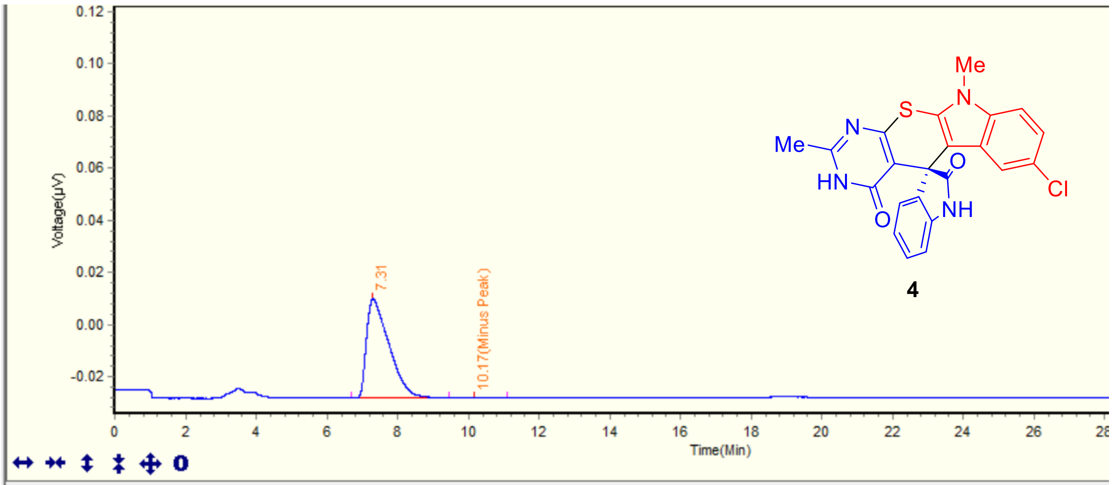

No.	Retention Time	Peak Area	Peak Height	Peak Area(%)	Peak Width
1	7.31	836818	19033	99.79%	2.766 BB
2	10.17	1786	50	0.21%	1.645 BB
Total		838,604	19,083	100.00%	

Single-crystal X-ray diffraction of 3g (CCDC 2177975)

3g with ellipsoid contour at 30% probability level

X-ray analysis was carried out using the single crystal which was grown in Hexane/Acetone.

The instrumentation used for the crystal measurement is Bruker APEX-II CCD.

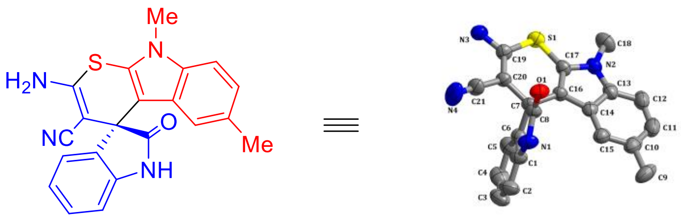

3g

checkCIF/PLATON report

You have not supplied any structure factors. As a result the full set of tests cannot be run.

THIS REPORT IS FOR GUIDANCE ONLY. IF USED AS PART OF A REVIEW PROCEDURE FOR PUBLICATION, IT SHOULD NOT REPLACE THE EXPERTISE OF AN EXPERIENCED CRYSTALLOGRAPHIC REFEREE.

No syntax errors found. CIF dictionary Interpreting this report

Datablock: mo_20220607c_0ma

Bond precision: C-C = 0.0058 A Wavelength=0.71073

Cell:	a=30.2442(9)	b=14.4397(4)	c=10.3922(2)
	alpha=90	beta=102.923(2)	gamma=90
Temperature:	296 K		

	Calculated	Reported
Volume	4423.5(2)	4423.5(2)
Space group	C 2	C 1 2 1
Hall group	C 2y	C 2y
Moiety formula	C21 H16 N4 O S [+ solvent]	C21 H16 N4 O S
Sum formula	C21 H16 N4 O S [+ solvent]	C21 H16 N4 O S
Mr	372.44	372.44
Dx,g cm-3	1.118	1.118
Z	8	8
Mu (mm-1)	0.162	0.162
F000	1552.0	1552.0
F000'	1553.51	
h,k,lmax	39,18,13	39,18,13
Nref	10261[5335]	10122
Tmin,Tmax	0.985,0.992	0.985,0.992
Tmin'	0.981	

Correction method= # Reported T Limits: Tmin=0.985 Tmax=0.992
AbsCorr = NONE

Data completeness= 1.90/0.99 Theta(max)= 27.583

R(reflections)= 0.0505(7757) wR2(reflections)= 0.1533(10122)

S = 1.025 Npar= 493

The following ALERTS were generated. Each ALERT has the format
test-name_ALERT_alert-type_alert-level.
Click on the hyperlinks for more details of the test.

Alert level C

```
PLAT230_ALERT_2_C Hirshfeld Test Diff for    C23      --C24      .        5.6 s.u.
PLAT334_ALERT_2_C Small <C-C> Benzene Dist.   C22      -C27      .       1.37 Ang.
PLAT340_ALERT_3_C Low Bond Precision on  C-C Bonds ...............    0.00576 Ang.
PLAT420_ALERT_2_C D-H Bond Without Acceptor  N7       --H7B      .     Please Check
```

Alert level G

```
PLAT003_ALERT_2_G Number of Uiso or Uij Restrained non-H Atoms ...          2 Report
PLAT007_ALERT_5_G Number of Unrefined Donor-H Atoms ..............          6 Report
PLAT066_ALERT_1_G Predicted and Reported Tmin&Tmax Range Identical          ? Check
PLAT128_ALERT_4_G Alternate Setting for Input Space Group  C2              I2 Note
PLAT178_ALERT_4_G The CIF-Embedded .res File Contains SIMU Records          1 Report
PLAT606_ALERT_4_G Solvent Accessible VOID(S) in Structure ........          ! Info
PLAT790_ALERT_4_G Centre of Gravity not Within Unit Cell: Resd.  #          2 Note
              C21 H16 N4 O S
PLAT791_ALERT_4_G Model has Chirality at C7          (Sohnke SpGr)          S Verify
PLAT791_ALERT_4_G Model has Chirality at C29         (Sohnke SpGr)          S Verify
PLAT860_ALERT_3_G Number of Least-Squares Restraints .............          7 Note
PLAT933_ALERT_2_G Number of HKL-OMIT Records in Embedded .res File         47 Note
```

```
   0 ALERT level A = Most likely a serious problem - resolve or explain
   0 ALERT level B = A potentially serious problem, consider carefully
   4 ALERT level C = Check. Ensure it is not caused by an omission or oversight
  11 ALERT level G = General information/check it is not something unexpected

   1 ALERT type 1 CIF construction/syntax error, inconsistent or missing data
   5 ALERT type 2 Indicator that the structure model may be wrong or deficient
   2 ALERT type 3 Indicator that the structure quality may be low
   6 ALERT type 4 Improvement, methodology, query or suggestion
   1 ALERT type 5 Informative message, check
```
